# Global burden and forecast of acute viral hepatitis B among women of childbearing age: a systematic analysis of the global burden of disease study 2021

**DOI:** 10.1186/s12879-026-13302-w

**Published:** 2026-05-16

**Authors:** Wei-dong Wu, Rui-xuan Li, Ding-qi Shi, Shah Dhruvi Chiragkumar, Yan-yan Gao, Jun-xiong Liu, Yao Xiao, Lei Si

**Affiliations:** 1 School of Health Management, Guangzhou Medical University, Guangzhou, China; 2 The Third School of Clinical Medicine, Guangzhou Medical University, Guangzhou, China; 3 School of Management, Beijing University of Traditional Chinese Medicine, Beijing, China; 4 Department of Hospital Administration, Gaoming District People’s Hospital, Foshan, Guangdong China; 5 School of Health Sciences, Western Sydney University, Campbelltown, Australia; 6 Translational Health Research Institute, Western Sydney University, Penrith, Australia

**Keywords:** Acute hepatitis B, Women of childbearing age, GBD, Joinpoint analysis, Age-period-cohort analysis, Bayesian age-period-cohort

## Abstract

**Background:**

Acute hepatitis B (AHB), an acute manifestation triggered by hepatitis B virus infection, has emerged as a significant international public health concern, particularly endangering women of childbearing age (WCBA) who are prone to persistent infection, adverse pregnancy outcomes, and mother-to-child transmission threats. However, research on acute hepatitis B lags, mainly focusing on chronic cases or clinical treatments. This study aims to quantify its global disease burden from 1990 to 2021 among women of childbearing age and predict epidemiological trends to inform targeted prevention and control strategies.

**Methods:**

Data of AHB burden from 1990 to 2021were obtained from the GBD 2021 via Global Health Data Exchange (GHDx). Frontier analysis was utilized to find the unrealized health potential. The age-period-cohort model was applied to analyze trends across different age groups, periods, and birth cohorts. The joinpoint regression model was used to identify significant changes in data trends over time. The Bayesian age-period-cohort model was utilized for forecasting future epidemiological trajectories.

**Results:**

A decreasing trend in age-standardized incidence rate (ASIR), age-standardized prevalence rate (ASPR), age-standardized mortality rate (ASMR), and disability-adjusted life-years rates (ASDR) was observed among the women of childbearing age worldwide from 1990 to 2021. The disease burden was disproportionately higher in low socio-demographic index (SDI) regions. The Bayesian age-period-cohort model revealed that by 2050, the ASIR and ASPR show similar downward trends, as do the ASMR and ASDR, but the latter decline less steeply than the former.

**Conclusions:**

The global disease burden of AHB in WCBA was declining, which is consistent with the vaccination and perinatal prevention, yet marked geographic disparities persist in low-resource regions. Modeling projections show that continued declines may contribute to progress toward the WHO viral hepatitis elimination goals, but vaccine coverage, safe injection and sufficient prevention of mother-to-child transmission remain essential.

**Clinical trial number:**

Not applicable.

**Supplementary information:**

The online version contains supplementary material available at 10.1186/s12879-026-13302-w.

## Introduction

Acute hepatitis B (AHB), a significant clinical manifestation of hepatitis B virus (HBV) infection, characterized by acute viral hepatitis leading to hepatic injury, is typically a self-limited condition in immunocompetent adults, with clinical resolution occurring within 6 months. If the initial infection lasts longer than 6 months, chronic HBV infection can develop, leading to liver cirrhosis, liver cancer, and even death [1]. Currently the World Health Organization (WHO) estimates that approximately 254 million individuals are living with chronic HBV infection in 2022, with associated mortality remaining high at 1.1 million annual deaths globally, constituting a major global public health challenge [2–4]. Previous studies have shown that approximately 1% of AHB cases present as severe or fulminant hepatitis, with a lethality rate of up to 80%, and that life-saving liver transplantation is often required [5–7]. While a minority of cases may present with severe or fulminant hepatitis, the majority of infections are asymptomatic (approximately 50–70%) or mildly symptomatic, resulting in many undiagnosed and unreported infections [8]. Unlike chronic hepatitis B, which represents cumulative historical infections, acute infections indicate recent transmission events and thus serve as a sentinel marker for ongoing transmission dynamics and missed prevention opportunities [9]. During the acute phase, patients typically exhibit high levels of viremia, rendering them highly infectious and posing substantial transmission risks to close contacts.[10]

Among women of childbearing age (WCBA), the impact of HBV warrants particular attention. Pregnant women infected with acute hepatitis B may be more prone to chronic HBV infection because pregnancy-associated immunological shift attenuates antiviral responses [11–13]. Compared with men, WCBA face persistent barriers to healthcare access and HBV screening in many settings [14]. Meta-analyses indicate that hepatitis B infection during pregnancy is associated with higher risks of gestational diabetes, preterm delivery, pre-eclampsia, and eclampsia, thereby increasing the incidence of adverse pregnancy outcomes [15, 16]. Additionally, mother-to-child transmission at birth is one of the most common transmission routes of hepatitis B worldwide. Among mothers infected with hepatitis B virus (HBsAg-positive), the risk of mother-to-child transmission ranges from 70% to 90% in those who are HBeAg-positive, reflecting high viral replication, whereas transmission risk among HBeAg-negative mothers is estimated at 10% to 40% [17]. The chronicity risk of the infection is 80–90% when the infection is acquired by perinatal transmission or in infancy (<1 years old) [18]. Therefore, detecting AHB among WCBA is not merely a measure of disease incidence, but an urgent trigger for interrupting MTCT through immediate initiation of antiviral prophylaxis and neonatal vaccination protocols [18, 19]. Additionally, WCBA represent a unique opportunity for population-based HBV surveillance. This is because their engagement with routine prenatal and reproductive care guarantees systematic screening and medical contact—a level of health system interaction that is typically absent in the general asymptomatic population [10]. Given its potential to harm both mothers and children by increasing the risk of adverse pregnancy outcomes and mother-to-child transmission, health issues such as AHB affecting WCBA demand serious attention and proactive measures. A survey among women in Senegal reported that only 1.6% had ever been tested for HBV, while the prevalence of hepatitis B infection reached 9.2% among women of reproductive age. Approximately 5% of women aged 15–49 years were identified as being at risk of mother-to-child transmission, highlighting substantial gaps in detection and prevention [20]. The well-being of WCBA has a bearing on the well-being of the next generation.

Countries have designated WCBA as a priority population for hepatitis B prevention and control. The WHO has proposed a plan for the elimination of viral hepatitis B, with the need to reduce the number of new cases of hepatitis B from 1.5 million to 170,000 and the number of deaths from 820,000 to 310,000 by 2030. In 2050, this will save nearly 23 million lives and prevent nearly 53 million new viral hepatitis infections and 15 million cases of cancer [2, 21–24].

Undoubtedly, exploring the epidemiological trends of AHB in WCBA is indispensable because it deepens our insight into the course of hepatitis B and rigorously appraises the impact of targeted prevention and treatment strategies for this vulnerable group. Notably, despite persistent surveillance of hepatitis B data, research has focused predominantly on chronic hepatitis B or comparative viral hepatitis studies, primarily because AHB poses distinct methodological challenges: (1) diagnostic ambiguity between acute infection and acute exacerbation of chronic infection, and (2) heterogeneity in clinical severity ranging from subclinical cases to fulminant liver failure, complicating standardized disease classification [22, 25–27]. Furthermore, both clinical research and population-level epidemiological analyses are essential for understanding acute hepatitis B. However, population-level projections remain relatively scarce, largely due to challenges in diagnosis and disease severity classification [5, 21, 28].

This study aims to quantify the global disease burden of acute hepatitis B among WCBA and project epidemiological trends through age-period-cohort analysis. The findings can inform targeted control strategies by highlighting underlying health inequities and guiding more efficient resource allocation for this vulnerable population.

## Methods

### Disease definition and study population

Disease causes are categorized into four levels, and AHB can be classified as a fourth-level cause under the infectious disease category [29]. In Global Burden of Disease (GBD) 2021, acute hepatitis B (AHB) was defined as the new hepatitis B virus (HBV) infection event and the individual in the initial acute phase of infection (typically ≤ 6 months), irrespective of clinical symptoms [30]. This is distinct from chronic HBV infection (long-term HBsAg carriage). HBV can be transmitted through medical procedures, unsafe injection practices, sexual contact, and mother-to-child transmission. The ICD-10 codes mapped to acute hepatitis B within the GBD framework primarily include codes corresponding to acute HBV infection (e.g., B16 series). Detailed cause mapping is described in the GBD 2021 methodology [31, 32].

This study focuses on women of childbearing age (WCBA) and AHB as the study population. According to the definition of the WHO, WCBA refer to the female development stage with reproductive ability from 15 to 49 years of age [33]. Additionally, the routine prenatal care received by women of childbearing age provides a unique channel for systematic HBV surveillance, which is otherwise uncommon in the general population.[10]

### Data sources

The 2021 GBD report, compiled by the Institute for Health Metrics and Evaluation (IHME), is an international collaborative research project that provides comprehensive epidemiological data on 371 diseases and injuries across 204 countries and regions, covering 811 subnational locations, along with 88 attributable risk factors [34].

The burden of AHB was estimated via Bayesian meta-regression models (DisMod-MR 2.1), accounting for location, age, sex, year, and pathogen. Detailed information on the estimated burden of AHB, including data input, processing, and modeling methods has been described in previous studies [31]. All data can be accessed through GBD 2021, including previously reported data, statistical modeling, and methodological information [30, 35]. Therefore, no ethical approval or informed consent is required for this study.

This study utilized the Global Health Data Exchange (GHDx) query tool to collect annual data on acute hepatitis B incidence and prevalence among women of childbearing age (15–49 years) from 1990 to 2021 across 204 countries and regions [30, 31, 36]. These regions were categorized into 21 GBD areas based on epidemiological similarity and geographical location. Furthermore, these areas and countries were further divided into five distinct quintiles via the Socio-Demographic Index (SDI): Low SDI, Low to Medium SDI, Medium SDI, High to Medium SDI, and High SDI [30]. The study focused on women aged 15 to 49, divided into seven GBD age groups with a 5-year interval: 15–19 years, 20–24 years, 25–29 years, 30–34 years, 35–39 years, 40–44 years, and 45–49 years.

### Socio-demographic index

The SDI assesses a country’s or region’s development level by incorporating the fertility rate, educational level, and per capita income data, facilitating health status comparisons across different regions over time. With a range of 0 (lowest socioeconomic status) to 1 (highest socioeconomic status), a higher SDI signifies more advanced socioeconomic development.

### Statistical analyses

Both the number and the rate are used to assess the burden of AHB. The rate is expressed as estimates per 100,000 people, whereas the number of cases reflects the absolute burden in terms of total cases. Both metrics are presented with their 95% uncertainty intervals (UIs). All analyses were conducted via appropriate statistical models, and *p* < 0.05 was considered statistically significant.

The age-standardized rate (ASR) is primarily used to eliminate or reduce the impact of age structure differences when different populations are compared, thereby making comparisons of health events (such as disease incidence and mortality rates) more equitable and accurate [37]. The Estimated Annual Percentage Change (EAPC) is calculated via time series data of ASRs, using the formula: 100 × [exp(β) − 1] [38].

Frontier analysis was used to capture the nonlinear relationship between the SDI and the age-standardized rate of disability-adjusted life years (ASDR), evaluating regional performance. Smoothing spline models were employed to explore the relationships between AHB burdens among WCBA and SDI levels.

The Joinpoint regression model is a collection of linear statistical models that were used to identify significant changes in data trends over time, distinguishing real shifts in trends from random variability [39]. This model identifies points with significant changes in trends (i.e., joinpoints), divides the overall trend into multiple subsegments based on the observed joinpoints, and calculates the annual percentage change (APC), average APC (AAPC) and the corresponding 95% confidence intervals (CIs) to quantify trend changes from 1990 to 2021. The Joinpoint regression program software and computing formulas can be obtained on their website [40].

The Age-period-cohort model was used to evaluate the associations of age, period, and birth cohort with AHB incidence and DALYs for deeper decomposition analysis. APC model analyses were conducted via the age-period-cohort Web Tool provided by the National Cancer Institute [41]. The Wald χ2 test was adopted to test the significance of the estimable parameters and functions. The detailed methods can be found in the introduction section of the age-period-cohort Web Tool and the related book on the APC model.[42]

This study utilized the Bayesian age-period-cohort (BAPC) model incorporating integrated nested Laplace approximations (INLA) to project future trends in AHB burden from 2022 to 2050 [43].

The detailed analysis methods are presented in the supplemental methods (Additional file 1). All the statistical analyses and visualizations were completed with R Studio software (version 4.4.2) with the R packages BAPC (version 0.0.36), INLA (version 24.05.011), ggplot2 (version 3.4.2), the age-period-cohort Web Tool, and Joinpoint software (version 5.3.0; National Cancer Institute, Rockville, MD, USA).

## Results

### Global level

**Fig. 1:**
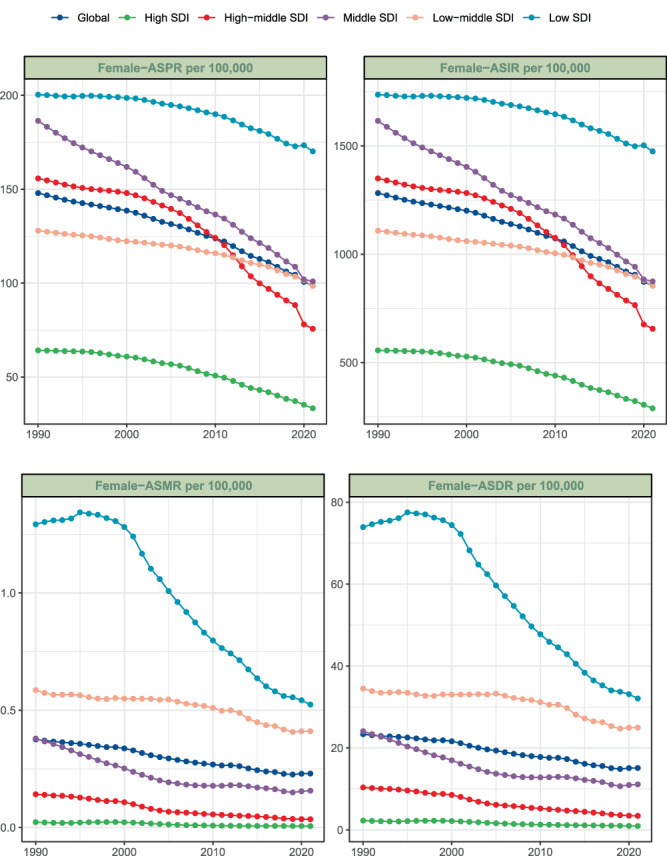
The ASR of AHB in global and the five SDI regions among WCBA (1990–2021). (AHB = acute hepatitis B, SDI = Socio-demographic Index, WCBA = women of childbearing age, ASR = age-standardized rate; ASPR = age-standardized rate of prevalence, ASIR = age-standardized rate of incidence, ASMR = age-standardized rate of mortality, ASDR = age-standardized rate of disability-adjusted life years)

In 2021, the global prevalence of acute hepatitis B in WCBA remained substantial, with a total of 1937055.6 cases (95% UI: 1021623.2-3585437.7), indicating a slight decrease from 2000240.7 cases (95% UI: 1045246-3614626.8) in 1990. Despite this inconspicuous decrease in the absolute number of cases, the age-standardized prevalence rate (ASPR) demonstrated a relatively significant decrease (by approximately 33%), from 147.94 per 100,000 persons (95% UI: 76.841–268.102) in 1990 to 98.743 per 100,000 persons (95% UI: 52.179–182.448) in 2021. The EAPC for ASPR was −1.23 (95% CI: −1.33 to −1.13) (Table 1, Fig. 1). The global incidence of acute hepatitis B reached 16787814.9 cases (95% UI: 8854068.2-31073793.3) in 2021, which has also slightly decreased from 17335419.7 cases (95% UI: 9058798.7-31326765.3) in 1990. The age-standardized incidence rate (ASIR) of acute hepatitis B exhibited a decline from 1282.146 per 100,000 persons (95% UI: 665.952–2323.554) in 1990 to 855.774 per 100,000 persons (95% UI: 452.222–1581.218) in 2021. During the study period, the EAPC in the ASIR was −1.23 (95% CI: −1.33 to −1.13), representing a consistent decrease in the incidence rate of acute hepatitis B (Table 1, Fig. 1). Mortality due to acute hepatitis B was estimated at 4485.2 deaths (95% UI: 3048.1–7017.2), with an age-standardized mortality rate (ASMR) of 0.23 per 100,000 persons (95% UI: 0.156–0.36) and an EAPC of −1.78 (95% CI: −1.85 to −1.71) (Table 1, Fig. 1), which was the most significant decrease in EAPC among the four measures. The global DALYs for acute hepatitis B in 2021 were 293132.1 (95% UI: 202783.1-443064.4), with an age-standardized DALYs rate (ASDR) of 15.114 per 100,000 persons (95% UI: 10.454–22.861) and an EAPC of −1.58 (95% CI: −1.66 to −1.5) (Table 1, Fig. 1).

**Table 1:** Global, SDI and regional trends of AHB burden in WCBA (1990–2021)

location	Number (95% UI) 1990	ASR (95% UI) 1990	Number (95% UI) 2021	ASR (95% UI) 2021	EAPC (95% CI) 1990–2021
**Prevalence**					
Global	2000240.7 (1045246-3614626.8)	147.94 (76.841–268.102)	1937055.6 (1021623.2-3585437.7)	98.743 (52.179–182.448)	−1.23 (−1.33 to −1.13)
Low SDI	225692 (117994.3-408147.6)	200.356 (103.354–365.38)	461209 (247954.8-834634.9)	170.17 (90.601–310.405)	−0.52 (−0.59 to −0.45)
Low-middle SDI	350377.8 (186489.8-627821)	127.93 (67.348–230.736)	496117 (267744.9-907458.6)	98.542 (53.072–180.608)	−0.73 (−0.81 to −0.65)
Middle SDI	839945.4 (435954.3-1524919.4)	186.407 (95.872–340.442)	640578 (330148.4-1198231.6)	100.945 (52.225–188.093)	−1.85 (−1.95 to −1.75)
High-middle SDI	437478.4 (225252.8-799178.4)	155.754 (79.991–284.799)	251133.3 (122926.6-481308.1)	75.684 (37.204–144.278)	−2.14 (−2.43 to −1.84)
High SDI	145592.8 (78673.6-256685.6)	64.195 (34.86–112.873)	86927.4 (46375.8-160883.8)	33.334 (17.847–61.394)	−2.01 (−2.24 to −1.78)
Andean Latin America	5700.1 (3123.4–10146.5)	60.472 (32.922–108.502)	8762.1 (4826.7–15871.4)	49.64 (27.346–89.922)	−0.58 (−0.66 to −0.49)
Australasia	3966.4 (1992.2–7220)	73.824 (37.143–134.006)	3525.8 (1718.3–6652.7)	45.397 (22.235–85.382)	−1.52 (−1.75 to −1.29)
Caribbean	2838.9 (1406.3–5276.9)	30.02 (14.742–56.026)	2809.8 (1410.2–5369.1)	23.25 (11.685–44.372)	−0.68 (−0.77 to −0.59)
Central Asia	20366 (10796.4–36079.4)	119.052 (62.819–212.131)	24451.6 (12860.9–45138)	97.305 (51.294–179.625)	−0.57 (−0.73 to −0.41)
Central Europe	20080.2 (10773.3–35833.3)	65.478 (35.221–116.594)	8139.7 (4224.9–15264.3)	28.248 (14.758–52.655)	−2.46 (−2.67 to −2.25)
Central Latin America	26250.3 (12148.8–51075.3)	61.434 (27.96–120.683)	22113.6 (9816.4–43228.6)	32.203 (14.31–62.903)	−1.96 (−2.2 to −1.72)
Central Sub-Saharan Africa	37674.4 (18317.3–69973.1)	303.271 (145.207–570.119)	90248.2 (45504-168151.7)	277.664 (138.016–520.998)	−0.29 (−0.36 to −0.23)
East Asia	893553.3 (455446.6-1625475.2)	265.713 (134.329–485.548)	451510.6 (215248.5-873589.8)	126.245 (60.616–242.482)	−2.2 (−2.39 to −2.02)
Eastern Europe	37647.4 (18252.9–69625.2)	67.683 (33.039–124.901)	21806.6 (10397.8–42124.3)	41.989 (20.2–80.275)	−1.09 (−1.49 to −0.68)
Eastern Sub-Saharan Africa	81579.6 (41711.3-148532.5)	187.897 (94.565–345.585)	146281.7 (75117.3-269686.9)	140.712 (71.525–261.78)	−0.8 (−0.9 to −0.69)
High-income Asia Pacific	50305 (27610.5–89563.9)	110.784 (60.846–197.114)	25511.4 (14161.1–46017.7)	63.875 (35.709–114.537)	−1.99 (−2.09 to −1.9)
High-income North America	15666.2 (8010.3–29029.9)	20.937 (10.767–38.666)	10550.4 (5279.9–19686)	11.955 (6.015–22.245)	−1.53 (−1.82 to −1.24)
North Africa and Middle East	98974.7 (54420.8-172732.7)	127.656 (69.404–224.706)	122804.4 (69112.5-219824.7)	76.123 (42.879–136.118)	−1.59 (−1.74 to −1.44)
Oceania	4151.3 (2031.2–7778.7)	263.293 (126.621–496.88)	6346.3 (3031.8–12076.1)	186.118 (88.516–355.247)	−1.22 (−1.39 to −1.05)
South Asia	250674.1 (131982.7-456270.7)	98.043 (51.07–179.472)	390131.3 (213001.3-706373.8)	78.825 (43.002–142.897)	−0.68 (−0.7 to −0.66)
Southeast Asia	224636.5 (119090-405618.1)	185.143 (97.348–335.937)	234904.2 (122832.1-432904.5)	126.314 (66.169–232.442)	−1.11 (−1.3 to −0.92)
Southern Latin America	2219.3 (1221.3–3948.5)	17.921 (9.846–31.933)	2631.5 (1421.7–4753.9)	14.662 (7.931–26.469)	−0.44 (−0.58 to −0.3)
Southern Sub-Saharan Africa	23887 (12291.4–43513.5)	177.078 (90.216–325.271)	24775.4 (12402.8–45837.9)	113.141 (56.637–209.252)	−1.43 (−1.56 to −1.31)
Tropical Latin America	26360.2 (13677.1–47190)	65.738 (33.873–118.165)	17815.4 (8302.5–35161.8)	28.037 (13.106–55.206)	−2.68 (−2.98 to −2.38)
Western Europe	36357 (19004.3–65924.7)	38.071 (19.996–68.86)	21607.3 (11174.8–40051.8)	21.841 (11.348–40.305)	−1.66 (−1.83 to −1.49)
Western Sub-Saharan Africa	137352.8 (73208.7-247504.8)	313.748 (164.406–570.619)	300328.5 (161804.9-542210.3)	256.486 (136.644–466.705)	−0.59 (−0.66 to −0.52)
**Incidence**					
Global	17335419.7 (9058798.7-31326765.3)	1282.146 (665.952–2323.554)	16787814.9 (8854068.2-31073793.3)	855.774 (452.222–1581.218)	−1.23 (−1.33 to −1.13)
Low SDI	1955997.5 (1022617.6-3537279)	1736.421 (895.733–3166.624)	3997144.5 (2148941.6-7233502.2)	1474.808 (785.205–2690.176)	−0.52 (−0.59 to −0.45)
Low-middle SDI	3036607.2 (1616244.8-5441115.3)	1108.725 (583.68–1999.711)	4299680.8 (2320456.1-7864640.8)	854.033 (459.958–1565.269)	−0.73 (−0.81 to −0.65)
Middle SDI	7279526.9 (3778270.9-13215968.2)	1615.53 (830.895–2950.494)	5551676.1 (2861285.9-10384673.6)	874.858 (452.62–1630.142)	−1.85 (−1.95 to −1.75)
High-middle SDI	3791479.1 (1952190.7-6926212.4)	1349.872 (693.255–2468.258)	2176488.7 (1065363.5-4171337)	655.926 (322.436–1250.411)	−2.14 (−2.43 to −1.84)
High SDI	1261804.5 (681838.1-2224608.7)	556.36 (302.121–978.229)	753370.7 (401923.5-1394326.2)	288.896 (154.676–532.083)	−2.01 (−2.24 to −1.78)
Andean Latin America	49401 (27069.7–87936.4)	524.093 (285.322–940.35)	75938.3 (41831.6-137551.9)	430.211 (236.998–779.324)	−0.58 (−0.66 to −0.49)
Australasia	34375.7 (17266–62573)	639.805 (321.905–1161.384)	30556.9 (14892–57657)	393.442 (192.704–739.978)	−1.52 (−1.75 to −1.29)
Caribbean	24603.7 (12188–45733.1)	260.172 (127.762–485.557)	24352 (12221.5–46532.2)	201.496 (101.27–384.554)	−0.68 (−0.77 to −0.59)
Central Asia	176505.2 (93568.9-312688.4)	1031.788 (544.431–1838.472)	211913.8 (111461.2-391196.3)	843.307 (444.547–1556.751)	−0.57 (−0.73 to −0.41)
Central Europe	174028 (93368.6-310555.2)	567.479 (305.248–1010.484)	70543.7 (36615.6-132290.9)	244.818 (127.906–456.344)	−2.46 (−2.67 to −2.25)
Central Latin America	227503 (105289.8-442652.5)	532.427 (242.318–1045.921)	191650.9 (85075.6-374647.6)	279.09 (124.018–545.161)	−1.96 (−2.2 to −1.72)
Central Sub-Saharan Africa	326511.3 (158750.2-606433.4)	2628.352 (1258.461–4941.033)	782150.7 (394367.8-1457314.7)	2406.419 (1196.137–4515.317)	−0.29 (−0.36 to −0.23)
East Asia	7744128.5 (3947204-14087451.9)	2302.847 (1164.184–4208.082)	3913092 (1865486.9-7571111.2)	1094.124 (525.336–2101.509)	−2.2 (−2.39 to −2.02)
Eastern Europe	326277.5 (158192-603418.5)	586.585 (286.34–1082.479)	188990.3 (90113.9-365077.3)	363.902 (175.062–695.719)	−1.09 (−1.49 to −0.68)
Eastern Sub-Saharan Africa	707023.2 (361497.8-1287281.4)	1628.438 (819.566–2995.067)	1267774.9 (651016.6-2337286.3)	1219.508 (619.887–2268.763)	−0.8 (−0.9 to −0.69)
High-income Asia Pacific	435976.7 (239291.1-776220.1)	960.128 (527.334–1708.322)	221098.7 (122729.3-398820.1)	553.583 (309.475–992.653)	−1.99 (−2.09 to −1.9)
High-income North America	135774 (69422.7-251592.6)	181.456 (93.31–335.103)	91436.5 (45759.4-170612.4)	103.609 (52.127–192.791)	−1.53 (−1.82 to −1.24)
North Africa and Middle East	857781 (471647.1-1497016.7)	1106.35 (601.505–1947.453)	1064304.9 (598975.2-1905147.6)	659.734 (371.62–1179.688)	−1.59 (−1.74 to −1.44)
Oceania	35977.5 (17603.3–67415)	2281.873 (1097.381–4306.293)	55001.1 (26275.3-104659.2)	1613.027 (767.141–3078.809)	−1.22 (−1.39 to −1.05)
South Asia	2172508.5 (1143849.7-3954346.3)	849.705 (442.608–1555.421)	3381138 (1846011.4-6121906)	683.152 (372.683–1238.445)	−0.68 (−0.7 to −0.66)
Southeast Asia	1946849.9 (1032113-3515356.8)	1604.571 (843.684–2911.454)	2035836 (1064545.3-3751839)	1094.723 (573.461–2014.499)	−1.11 (−1.3 to −0.92)
Southern Latin America	19234 (10584.9–34220)	155.311 (85.334–276.751)	22806.1 (12321.5–41200.8)	127.068 (68.738–229.397)	−0.44 (−0.58 to −0.3)
Southern Sub-Saharan Africa	207020.8 (106525.2-377116.7)	1534.679 (781.873–2819.013)	214719.9 (107491-397261.8)	980.553 (490.853–1813.515)	−1.43 (−1.56 to −1.31)
Tropical Latin America	228455.4 (118535.1-408979.9)	569.733 (293.564–1024.099)	154400.1 (71954.7-304735.8)	242.989 (113.588–478.45)	−2.68 (−2.98 to −2.38)
Western Europe	315093.8 (164704-571347.7)	329.95 (173.299–596.786)	187263.1 (96847.9-347115.3)	189.292 (98.352–349.311)	−1.66 (−1.83 to −1.49)
Western Sub-Saharan Africa	1190390.9 (634475.2-2145041.7)	2719.15 (1424.855–4945.365)	2602847 (1402309.2-4699156.2)	2222.876 (1184.247–4044.772)	−0.59 (−0.66 to −0.52)
**Deaths**					
Global	4823.7 (2722.2–7505.2)	0.376 (0.21–0.583)	4485.2 (3048.1–7017.2)	0.23 (0.156–0.36)	−1.78 (−1.85 to −1.71)
Low SDI	1308.2 (358.6–2393.3)	1.293 (0.345–2.362)	1352.4 (798.3–2227.8)	0.524 (0.31–0.867)	−3.41 (−3.72 to −3.1)
Low-middle SDI	1491.2 (802.5–2886.5)	0.586 (0.312–1.133)	2045.9 (1146.7–3653.9)	0.411 (0.23–0.734)	−1.12 (−1.28 to −0.96)
Middle SDI	1587.5 (1056.6–2251.5)	0.379 (0.249–0.538)	959.2 (539.8–1405.5)	0.157 (0.087–0.23)	−2.97 (−3.27 to −2.68)
High-middle SDI	383 (258.1–537.9)	0.142 (0.095–0.199)	109.6 (71.9–162.3)	0.035 (0.023–0.053)	−4.85 (−5.06 to −4.63)
High SDI	52.3 (33.7–80.9)	0.023 (0.015–0.035)	16.8 (12–23.9)	0.006 (0.004–0.009)	−5.21 (−5.83 to −4.58)
Andean Latin America	0.8 (0.3–1.6)	0.009 (0.004–0.019)	1 (0.5–1.8)	0.006 (0.003–0.01)	−1.81 (−2.24 to −1.38)
Australasia	0.2 (0.1–0.3)	0.004 (0.003–0.006)	0.2 (0.1–0.3)	0.002 (0.001–0.003)	−3.24 (−4.84 to −1.61)
Caribbean	2.9 (1.4–6.2)	0.033 (0.016–0.069)	5.7 (2.7–12)	0.046 (0.022–0.098)	0.84 (0.5 to 1.18)
Central Asia	216.5 (174.3–267.4)	1.199 (0.963–1.485)	37.6 (27.1–50.2)	0.154 (0.111–0.207)	−6.72 (−6.85 to −6.59)
Central Europe	5.2 (3.9–7)	0.017 (0.013–0.023)	2.3 (1.8–2.9)	0.009 (0.007–0.011)	−2.89 (−3.58 to −2.2)
Central Latin America	1 (0.7–1.3)	0.003 (0.002–0.004)	5.9 (4.5–7.6)	0.009 (0.007–0.011)	4.89 (3.8 to 5.99)
Central Sub-Saharan Africa	103.1 (19–258)	0.905 (0.165–2.242)	81.7 (27.8–168.4)	0.265 (0.09–0.539)	−3.84 (−4.24 to −3.44)
East Asia	1051.2 (523.8–1618.4)	0.35 (0.175–0.537)	81.9 (55.9–115.3)	0.022 (0.015–0.03)	−9.37 (−9.95 to −8.79)
Eastern Europe	52.3 (44.2–61.7)	0.101 (0.086–0.119)	9.4 (6.8–12.8)	0.021 (0.015–0.028)	−5.64 (−6.59 to −4.69)
Eastern Sub-Saharan Africa	390 (137.5–773.6)	0.992 (0.344–1.956)	436.9 (185.1–774.7)	0.433 (0.184–0.769)	−3.27 (−3.52 to −3.01)
High-income Asia Pacific	10.5 (5.4–19.6)	0.022 (0.011–0.042)	2.2 (1.8–2.6)	0.005 (0.004–0.006)	−7.53 (−9.32 to −5.7)
High-income North America	5.6 (4.6–6.9)	0.007 (0.006–0.009)	2.8 (2.1–3.5)	0.003 (0.002–0.004)	−4.08 (−4.8 to −3.35)
North Africa and Middle East	629.4 (205.5–1094.3)	0.982 (0.319–1.682)	426.3 (231.3–746.9)	0.269 (0.146–0.472)	−4.27 (−4.39 to −4.14)
Oceania	1.4 (0.6–3.2)	0.096 (0.041–0.224)	0.7 (0.2–1.8)	0.02 (0.007–0.052)	−5.32 (−5.5 to −5.14)
South Asia	1111.5 (415.1–2667.5)	0.447 (0.163–1.084)	2701.7 (1441.2–4846.1)	0.55 (0.292–0.987)	0.78 (0.52 to 1.03)
Southeast Asia	360.6 (201.1–661.1)	0.316 (0.178–0.575)	229.6 (103.1–497.2)	0.122 (0.055–0.265)	−3.43 (−3.64 to −3.21)
Southern Latin America	0.8 (0.3–1.9)	0.006 (0.002–0.016)	0.3 (0.2–0.5)	0.002 (0.001–0.003)	−5.05 (−5.8 to −4.3)
Southern Sub-Saharan Africa	29.3 (18–46.1)	0.255 (0.158–0.402)	18.6 (11.8–30)	0.088 (0.056–0.142)	−2.38 (−3.23 to −1.52)
Tropical Latin America	5.5 (4.1–7.4)	0.015 (0.011–0.02)	8.9 (7.4–10.7)	0.014 (0.012–0.017)	−1.16 (−2.11 to −0.2)
Western Europe	5.8 (4.2–8.1)	0.006 (0.004–0.008)	1.9 (1.5–2.3)	0.002 (0.002–0.002)	−4.76 (−5.65 to −3.85)
Western Sub-Saharan Africa	840.1 (274.6–1699.6)	2.106 (0.677–4.289)	429.9 (255.7–674.8)	0.378 (0.225–0.596)	−5.96 (−6.41 to −5.51)
**Disability-adjusted life years**					
Global	307355 (184424.1-469224.7)	23.366 (13.937–35.558)	293132.1 (202783.1-443064.4)	15.114 (10.454–22.861)	−1.58 (−1.66 to −1.5)
Low SDI	78261.9 (23706.3-140689)	73.922 (21.996–132.642)	85955.7 (52128-137098.2)	32.079 (19.522–51.265)	−3.19 (−3.49 to −2.89)
Low-middle SDI	91188.2 (51083.2-172023)	34.513 (19.182–64.834)	125983.8 (72944-217839.4)	24.982 (14.451–43.202)	−1.01 (−1.2 to −0.83)
Middle SDI	104088.7 (72662.7-143316.2)	24.093 (16.67–33.082)	67598.3 (41282.6–96166)	11.111 (6.694–15.819)	−2.56 (−2.78 to −2.33)
High-middle SDI	28479.6 (20130.9–39079.6)	10.37 (7.3–14.241)	10936.4 (6817.3–17547.9)	3.437 (2.126–5.48)	−3.8 (−3.95 to −3.64)
High SDI	5236.4 (3360.8–8049.4)	2.279 (1.467–3.497)	2563 (1531.3–4327)	0.947 (0.57–1.591)	−3.13 (−3.42 to −2.83)
Andean Latin America	131.1 (65–232.1)	1.494 (0.735–2.657)	210.4 (104.5–392.7)	1.201 (0.597–2.239)	−0.86 (−1.03 to −0.69)
Australasia	75.1 (31.7–149)	1.377 (0.586–2.723)	77.2 (29.4–159.1)	0.974 (0.377–1.997)	−1.22 (−1.34 to −1.11)
Caribbean	206.4 (111–396.3)	2.295 (1.236–4.36)	368.7 (190–733.3)	3.03 (1.558–6.041)	0.73 (0.44 to 1.03)
Central Asia	13913.1 (11259.4–17078.6)	75.927 (61.313–93.332)	2669 (1929.1–3598.2)	10.967 (7.925–14.792)	−6.36 (−6.5 to −6.22)
Central Europe	636 (418.1–994)	2.059 (1.366–3.186)	295.6 (184–492.3)	1.059 (0.681–1.711)	−2.44 (−2.84 to −2.04)
Central Latin America	422.2 (177.2–875.5)	1.084 (0.451–2.259)	721.1 (427.8–1240.7)	1.046 (0.621–1.8)	0.32 (−0.08 to 0.72)
Central Sub-Saharan Africa	6550.7 (1514.8–15806.3)	55.147 (12.707–131.546)	6094.1 (2592–11454.1)	19.242 (8.228–35.727)	−3.31 (−3.66 to −2.97)
East Asia	70906 (40125.1-103719)	22.864 (12.995–33.279)	12823.1 (7476.1–22624.8)	3.424 (2.013–5.993)	−6.42 (−6.84 to −6)
Eastern Europe	3931.5 (3235.9–4890.5)	7.573 (6.288–9.315)	967.3 (637.9–1491.2)	2.021 (1.364–3.011)	−4.74 (−5.5 to −3.97)
Eastern Sub-Saharan Africa	24122.3 (9106.7–46911.7)	58.213 (21.798–112.579)	28104.8 (12901.5–48440.5)	26.748 (12.435–45.962)	−3.06 (−3.3 to −2.81)
High-income Asia Pacific	1374.5 (771.6–2318.9)	2.951 (1.65–4.994)	588.7 (316.1–1060.4)	1.338 (0.72–2.416)	−3.77 (−4.63 to −2.9)
High-income North America	577.9 (408–875.5)	0.761 (0.541–1.142)	358.3 (221.1–607)	0.402 (0.25–0.675)	−2.43 (−2.75 to −2.1)
North Africa and Middle East	35039.6 (12566.7–60469.2)	52.119 (18.532–88.518)	24767.9 (14191.7–41931.9)	15.585 (8.903–26.46)	−3.98 (−4.1 to −3.85)
Oceania	137.1 (69.3–259.2)	9.33 (4.678–17.759)	145.1 (57.7–304.7)	4.331 (1.725–9.097)	−2.63 (−2.71 to −2.54)
South Asia	69954.8 (28608-161154.1)	27.247 (10.977–63.184)	165523.5 (90842.2-290751.4)	33.357 (18.278–58.662)	0.76 (0.48 to 1.04)
Southeast Asia	24393.6 (14507.7–42576.9)	20.784 (12.493–35.938)	16686 (8612.5–31749.5)	8.95 (4.616–17.061)	−3.02 (−3.21 to −2.83)
Southern Latin America	75.3 (37.7–145.1)	0.62 (0.311–1.193)	65.6 (35.4–117.1)	0.362 (0.195–0.647)	−2.13 (−2.5 to −1.77)
Southern Sub-Saharan Africa	1956.8 (1264.6–2918.9)	16.327 (10.602–24.327)	1389.1 (900.3–2134.8)	6.478 (4.206–9.945)	−2.18 (−2.87 to −1.48)
Tropical Latin America	698 (443.1–1123.9)	1.853 (1.177–2.984)	822.8 (578.6–1232.1)	1.295 (0.913–1.931)	−1.65 (−2.26 to −1.04)
Western Europe	924.6 (562.2–1562.3)	0.957 (0.586–1.61)	523.3 (276.7–971.4)	0.508 (0.274–0.933)	−2.4 (−2.78 to −2.02)
Western Sub-Saharan Africa	51328.3 (17897.5-101928.1)	122.77 (42.526–244.741)	29930.6 (18704.4–44825.2)	25.636 (16.101–38.311)	−5.52 (−5.92 to −5.11)

(SDI: Socio-Demographic Index, ASR: Age-standardized rate, EAPC: Estimated Annual Percentage Change, UI: uncertainty intervals, CI: confidence intervals, DALYs: disability-adjusted life years, AHB: Acute hepatitis B, WCBA: Women of childbearing age)

### Regional level

**Fig. 2:**
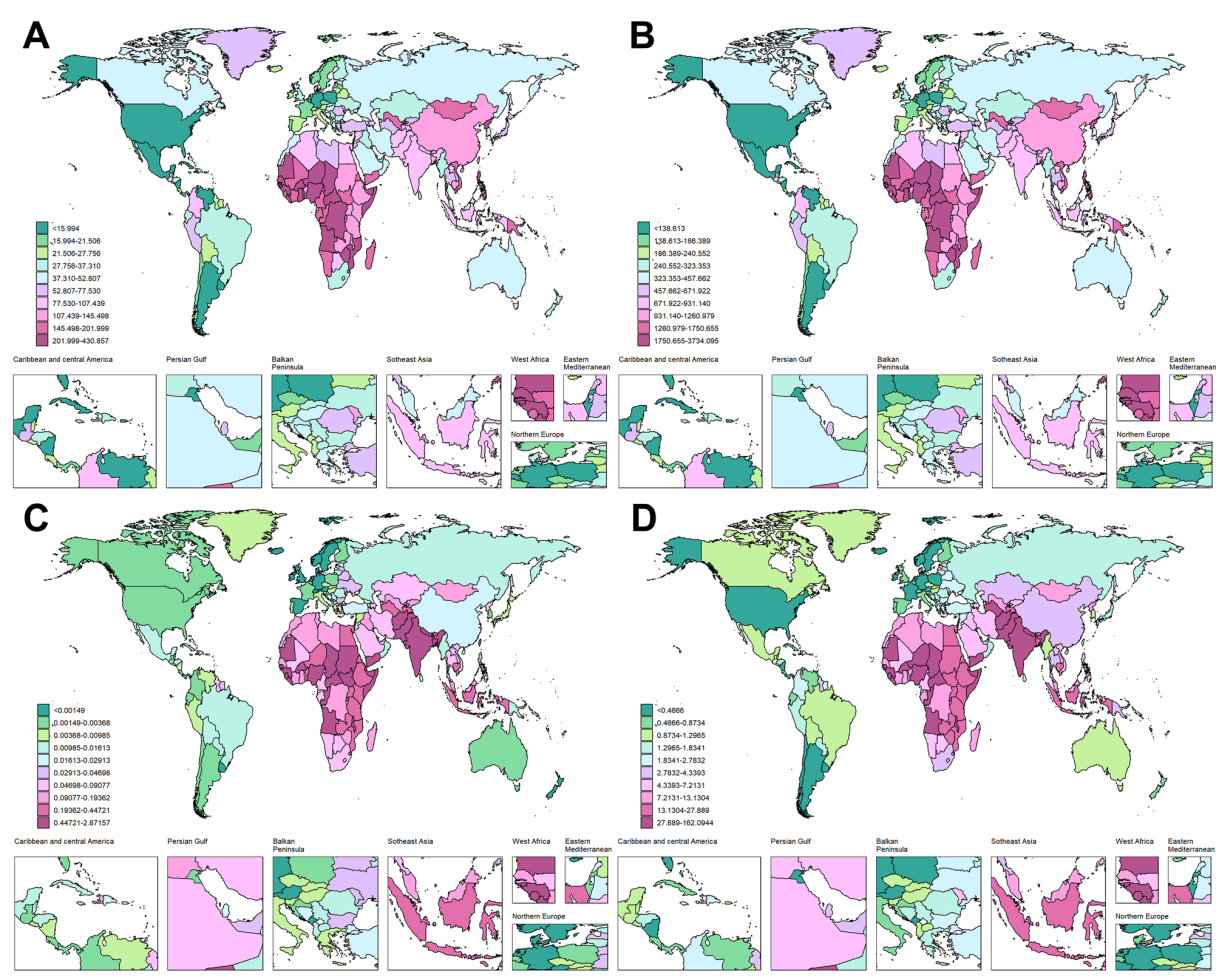
Map of age-standardized rates in 2021 for AHB in WCBA in 204 countries and territories. (**A**: Prevalence, **B**: Incidence, **C**: Mortality, **D**: DALYs; AHB = acute hepatitis B, WCBA = women of childbearing age, DALYs = disability-adjusted life years)

**Fig. 3:**
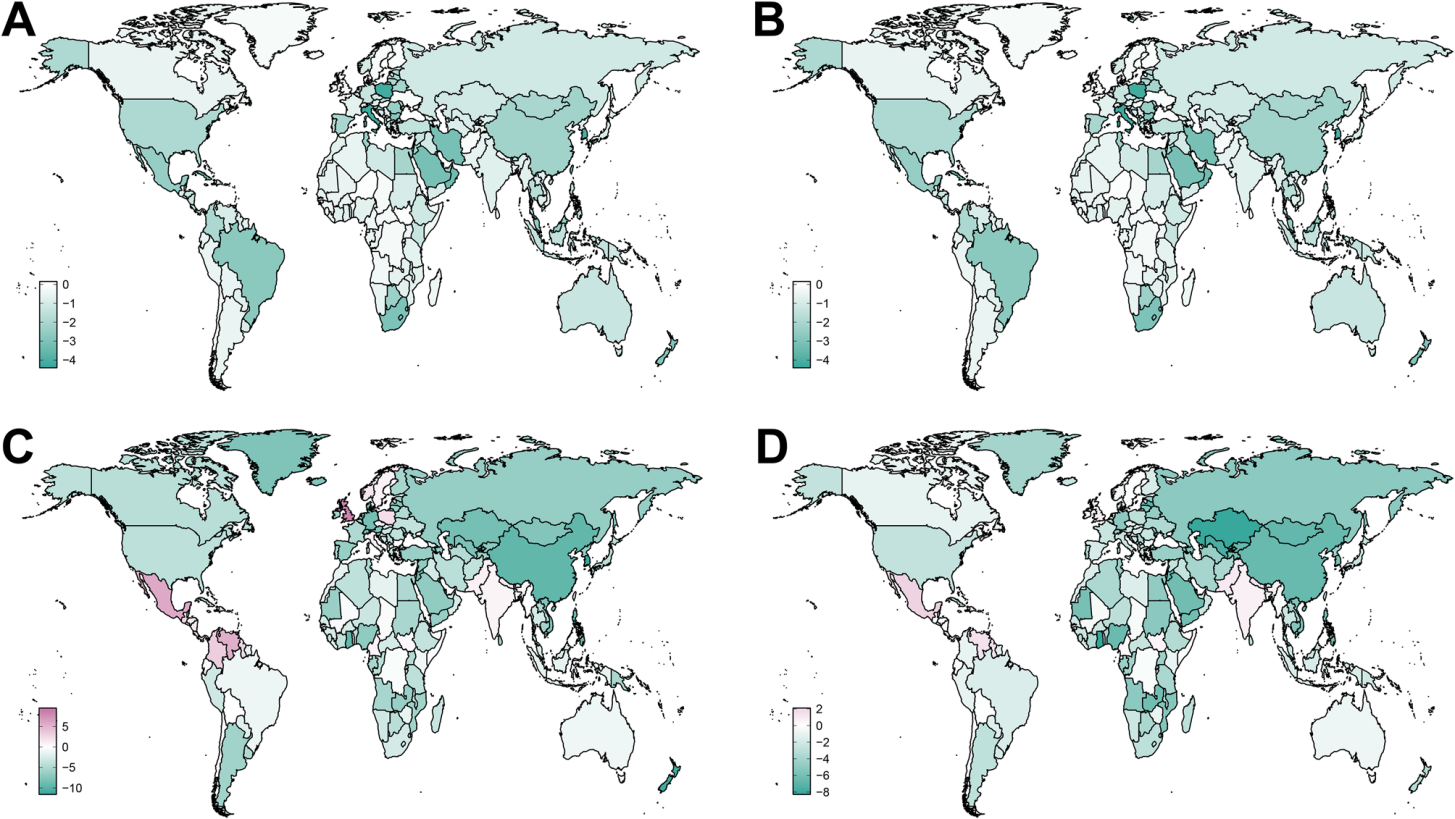
Map of EAPC for AHB in WCBA in 204 countries and territories (1990–2021). (**A**: Prevalence, **B**: Incidence, **C**: Mortality, **D**: DALYs; EAPC = Estimated Annual Percentage Change, AHB = Acute hepatitis B, WCBA = women of childbearing age, DALYs = disability-adjusted life years)

The global burden of acute hepatitis B exhibits distinct regional patterns that closely mirror SDI levels. The ASPR demonstrated notable disparities, with Low SDI regions bearing the highest rate at 170.17 per 100,000 people (95% UI: 90.601–310.405), whereas High SDI regions reported the lowest at 33.334 per 100,000 (95% UI: 17.847–61.394) (Table 1, Figs. 1, 2.B). Temporal trends in ASPR all decreased in five SDI levels. High-middle SDI regions showed the most obvious decline, with an EAPC of −2.14 (95% CI: −2.43 to −1.84). In these regions, the ASPR decreased from 155.754 per 100,000 people (95% UI: 79.991–284.799) in 1990 to 75.684 per 100,000 people (95% UI: 37.204–144.278) in 2021 (Table 1, Fig. 1). In contrast, Low SDI regions exhibited the lowest decrease in magnitude, with an EAPC of −0.52 (95% CI: −0.59 to −0.45) (Table 1, Fig. 3). The temporal trends in ASIR of five SDI levels, substantially is the same as ASPR. The ASIR in low SDI regions was 1474.808 per 100,000 people (95% UI: 785.205–2690.176), whereas High SDI regions reported a considerably lower rate of 288.896 per 100,000 people (95% UI: 154.676–532.083) (Table 1, Figs. 1, 2.A). The ASMR and ASDR also further emphasized these regional disparities. Low SDI regions reported the highest ASMR, whereas High SDI regions presented the lowest. Specifically, the ASMR in Low SDI regions was 0.23 per 100,000 people (95% UI: 0.156–0.36), as compared to 0.006 per 100,000 people (95% UI: 0.004–0.009) in High SDI regions, despite both rates being not very high (Table 1, Figs. 1, 2.C). For the ASDR, Low SDI regions bore the highest burden with an ASDR of 15.114 per 100,000 people (95% UI: 10.454–22.861), whereas High SDI regions presented the lowest burden at 0.947 per 100,000 people (95% UI: 0.57–1.591) (Table 1, Figs. 1, 2.D). Notably, when comparing among different SDI curves, in both ASMR and ASDR, as the SDI increases, the disease burden decreases. However, ASPR and ASIR were not like this in the early stage, which were presented by the High-middle SDI with the second-highest disease burden. Overall, the ASRs of the four measures in low and low-middle SDI regions consistently surpassed the global average, whereas that in the high SDI region was the lowest. Over the study years, all five SDI regions displayed a continuous downward trend in the ASRs for all four measures, whereas some higher SDI regions showed a more significant trend.

### National level

Overall, at the national level, the distributions of the four measures are similar, with low SDI regions such as Africa experiencing the highest age-standardized rates (ASPR, ASIR, ASMR, and ASDR), whereas other high SDI regions have less impact. When analyzing the EAPC, the four measures are generally negative in all countries, with only a few countries having positive values. (Fig. 3, Additional file 2: Table S1) In terms of the EAPC distribution, the incidence rates and prevalence rates exhibit similarities, as do the Death rates and DALYs rates.

The ASPR of acute hepatitis B varies from approximately 430.857 to 7.137 per 100,000 people. Among all the countries, the Republic of Zimbabwe (430.857 per 100,000 persons; 95% UI: 206.656–817.674), the Islamic Republic of Mauritania (344.781 per 100,000 persons; 95% UI: 182.897–629.241), and the Democratic Republic of the Congo (306.253 per 100,000 persons; 95% UI: 149.869–578.824) share the highest ASPR (Fig. 2. B, Additional file 2: Table S1). The highest ASPR values can be observed in three African countries. The consistency between country-level and regional data emphasized the major challenge linked to acute hepatitis B in African nations. The only two increases in the ASPR were found in the United Kingdom of Great Britain and Northern Ireland (EAPC 0.15, 95% CI 0.12 to 0.18) and the Kingdom of Denmark (EAPC 0.09, 95% CI 0.03 to 0.14) (Additional file 2: Table S1). Although the values were positive, the increases were not statistically significant.

The country-specific distribution of ASIR is presented in Fig. 2. A and Additional file 2: Table S2. The Republic of Zimbabwe had the highest ASIR (3734.095 per 100,000 persons; 95% UI: 1791.016–7086.511), whereas the Republic of Cuba had the lowest (61.852 per 100,000 persons; 95% UI: 24.641–127.919). The wide range of incidence rates and the notable decrease in prevalence across countries indicate that there may be considerable differences in risk factors, healthcare systems, and prevention strategies among nations.

The 2021 data for ASMR and ASDR due to acute hepatitis B reflect a notable consistency among the countries most affected. Somalia, Afghanistan, and Pakistan stand out prominently in two measures. Afghanistan ranks first in ASMR (2.872 per 100,000 persons; 95% UI: 0.638–6.882) and second in ASDR (149.61 per 100,000 persons; 95% UI: 34.189–357.91), whereas Somalia ranks second in ASMR (2.812 per 100,000 persons; 95% UI: 0.381–6.813) and first in ASDR (162.094 per 100,000 persons; 95% UI: 24.579–387.183). Pakistan in both categories shares the third highest ASMR (1.152 per 100,000 persons; 95% UI: 0.358–2.492) and the third highest ASDR rate (62.786 per 100,000 persons; 95% UI: 20.519–133.951) (Fig. 2.D, Additional file 2: Table S4). The trend of age-standardized ASDR decreased the most in the Republic of Ghana (EAPC −8.27, 95% CI −8.73 to −7.82), the Republic of Kazakhstan (EAPC −8.13, 95% CI −8.59 to −7.66), and Brunei Darussalam (EAPC −7.98, 95% CI −8.39 to −7.57) (Fig. 3.D and Additional file 2: Table S4).

### Age-period-cohort analyses

Globally and in the five SDI regions, Local Drifts show negative results across all age groups, indicating that a consistent downward trend exists in both incidence and DALYs.

**Fig. 4:**
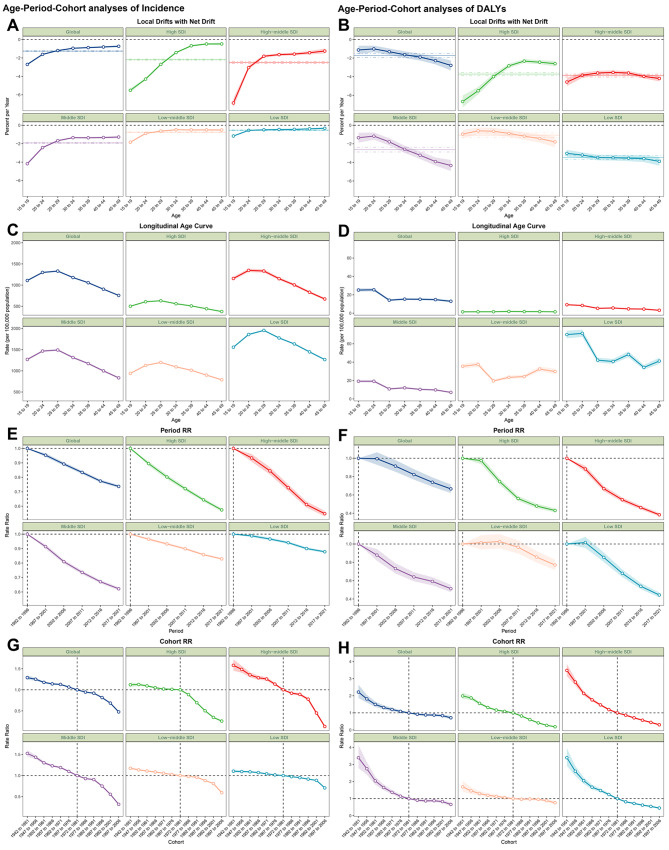
Age-period-cohort analyses on AHB incidence and DALYs in WCBA by SDI quintile (1992–2021). (**AB**: local drift and net drift, **CD**: age effect was depicted through the longitudinal age curves, **EF**: period effects were shown through the period RR, **GH**: birth cohort effects were described by the cohort RR; the horizontal dotted-dashed lines in panel a presented the net drift and corresponding 95% CI, while the dots-linking lines with their shaded areas presented the local drift, rates or rate ratios with their corresponding 95% CIs. AHB = acute hepatitis B; WCBA = women of childbearing age)

In terms of incidence, the degree of decline varies across different SDI regions, with higher SDI regions experiencing a greater degree of decline, and this phenomenon is more pronounced in the 15–29 age group. This decreasing trend exhibits a gradual attenuation with the ascent of the age spectrum (Fig. 4.A). For DALYs, there is no absolute relationship between the SDI, age group, and degree of decline. Even in the Global-level and Middle SDI regions, the decline clearly tends to gradually increase with the rise of the age spectrum (Fig. 4.B).

Overall, the age effect across different SDI regions displayed a similar pattern in incidence. (Fig. 4 C: all six panels) Initially, it increases until it peaks at ages 25–29 years, after which it subsequently decreases as age increases, and the lowest point is at ages 45–49 years for each region. In addition, compared with the global level and the other four SDI regions, the high SDI region presented lower incidence rates across all age groups, with relatively small disparities observed between different age groups within these regions (Fig. 4.C). For DALYs, there is generally a decreasing trend with advancing age. However, in the high and high-middle SDI regions, this trend is less pronounced, with the curves appearing almost flat. The most significant changes across all age groups occur between the 20–24 and 25–29 age brackets, particularly in low SDI areas. When the five SDI regions were compared, a clear pattern emerged: higher SDI levels were associated with lower DALYs, indicating a negative correlation between the SDI and DALYs (Fig. 4.D).

Period effects generally showed a persistent declining risk of incidence over the study period, and this pattern was similarly observed across the five SDI regions and at the global level. Furthermore, the curve also revealed a positive correlation between the SDI and the degree of decline over time (Fig. 4.E). For DALYs, the period effect indicated a consistent global downward trend, closely resembling that seen in high, high-middle, and middle SDI regions, while low and low-middle SDI regions exhibited unfavorable period risks. Compared with individuals in the reference period of 1992–1996, the relative period risk for individuals in the periods of 1997–2001 and 2002–2006 were respectively 1.015 (95% CI: 0.940 to 1.095) and 1.027 (95% CI: 0.952 to 1.108) in the low-middle SDI region. In the period of 1997 – 2001, there was 1.016 (95% CI: 0.957 to 1.078) in the low SDI region (Fig. 4.F).

Regarding the birth cohort effect, there was an overall persistent decline in incidence and DALYs across consecutive birth cohorts in the global and 5 SDI regions. Notably, across successive birth cohorts, the degree of decline in incidence became more pronounced, whereas the decline in DALYs reflects a gradual deceleration. (Fig. 4GH).

### Trends between SDI and AHB burden

**Fig. 5:**
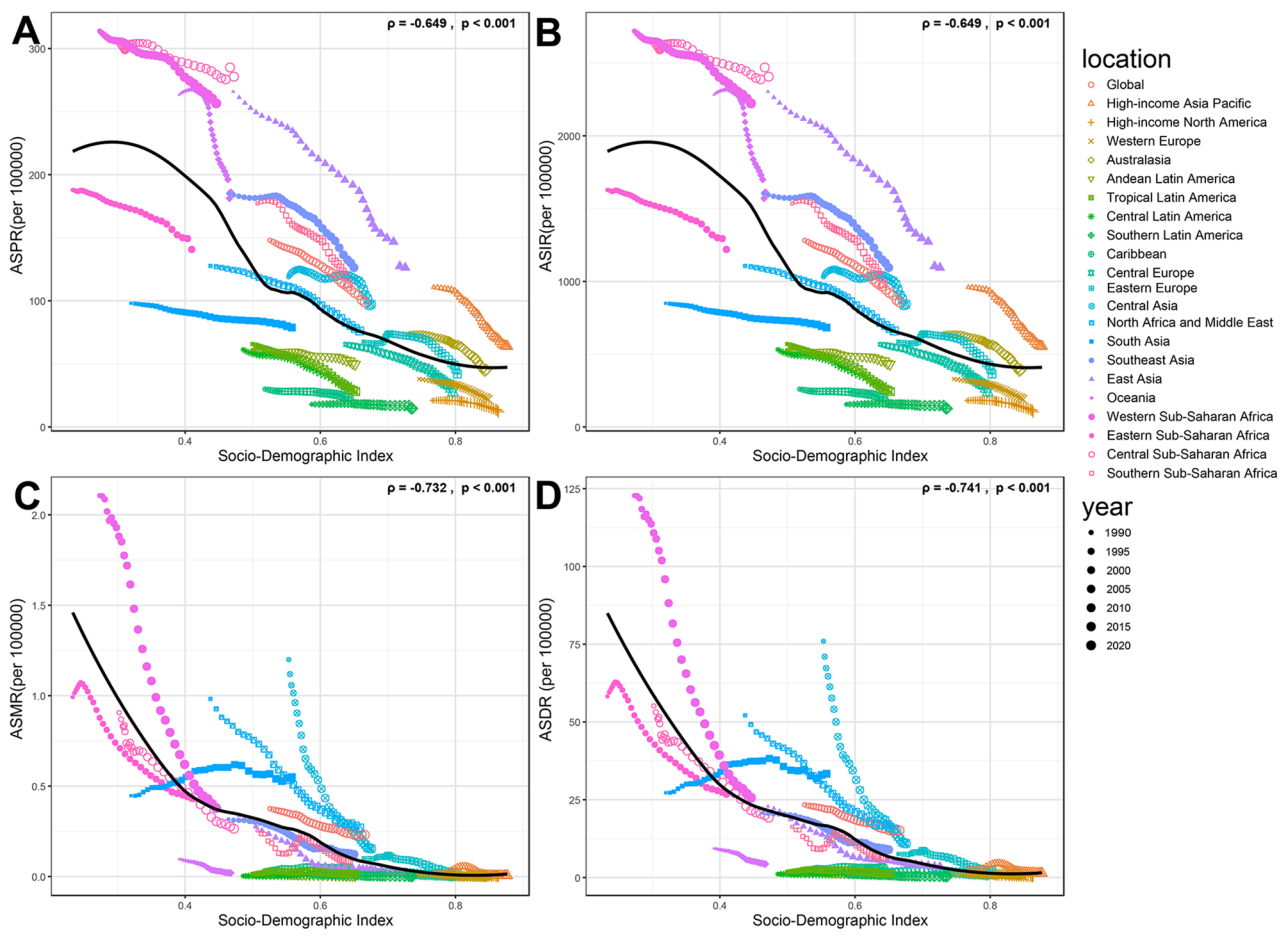
Global and regional total burdens related to AHB in WCBA by SDI from 1990 to 2021. (**A**: Age-standardized rate of prevalence, **B**: Age-standardized rate of incidence, **C**: Age-standardized rate of mortality, **D**: Age-standardized rate of disability-adjusted life years; AHB = acute hepatitis B, WCBA = women of childbearing age, SDI = Socio-demographic Index)

Overall, the distributions of the four measures at global and regional levels are similar, showing that low SDI regions suffer a substantially higher disease burden, while high SDI regions are less impacted. This pattern means that as SDI increases, the values of ASPR, ASIR, ASDR, and ASMR generally decrease. For instance, within East Asia, the ASPR shows a pronounced decline over time as SDI rises. Similarly, within Western Sub-Saharan Africa, the ASMR demonstrates a sharp decrease alongside SDI improvement. Moreover, with socio-economic development across regions, the burden of acute hepatitis B in WCBA has steadily declined over time. (Fig. 5)

### Frontier analysis

**Fig. 6:**
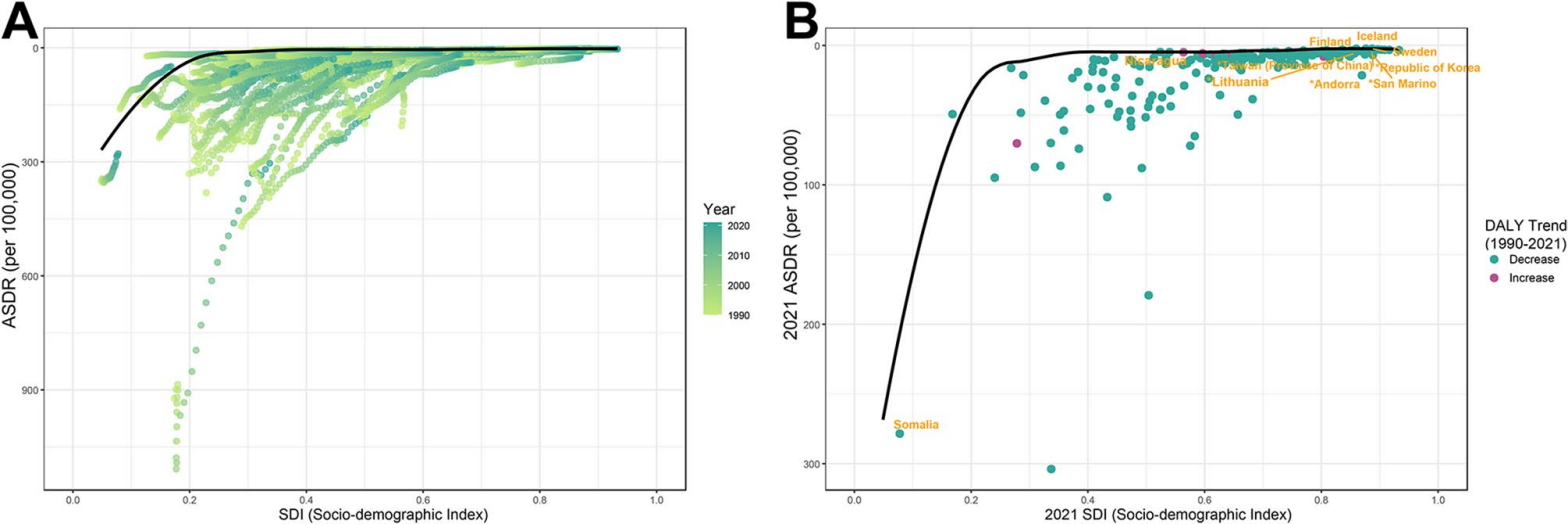
Frontier analysis of national burden exploring the relationship between SDI and ASDR. (**A**: Temporal trajectories for all regions, colored by year. **B**: Cross-sectional snapshot of ASDR in 2021; the line is the expected frontier. The labeled regions in panel **B**: Asterisks* mark the top 5 high SDI regions with the most significant deviation from the frontier, indicating potential for improved efficiency, while other 5 regions closest to the frontier are labeled without asterisks, representing near-optimal outcomes relative to their SDI. SDI = Socio-demographic Index, ASDR = age-standardized rate of disability-adjusted life years, AHB = acute hepatitis B, WCBA = women of childbearing age)

The color gradient in Fig. 6. A illustrates the progression of years, ranging from light shades (yellow-green) representing 1990 to the darkest shades (dark-green) denoting 2021. As the years changed from 1990 to 2021, the SDI increased, and the ASDR exhibited an overall downward trend, indicating a good global development and a general decrease in DALYs.

In Fig. 6. B, each dot signifies a specific region or territory for the year 2021, with the 5 high SDI (>0.85) regions displaying the most significant deviation from the frontier labeled and connected with “*” at the top, whereas the other 5 countries labeled without “*” are the closest top 5 to the frontier. The distance from the frontier line (marked with ‘*’) shows that, in 2021, disease control effectiveness was not commensurate with SDI, indicating substantial room for improvement. (*Principality of Andorra, *Republic of Lithuania, *Japan, *Republic of San Marino, *Taiwan Province of China) Correspondingly, the closest one has already spared no effort to act, with its current SDI situation. In addition, the green points constitute the majority, also indicating a general improvement in disease control. (Federal Republic of Somalia, Federal Republic of Germany, Bermuda, Eastern Republic of Uruguay, Republic of Nicaragua).

### Joinpoint regression analysis

**Fig. 7:**
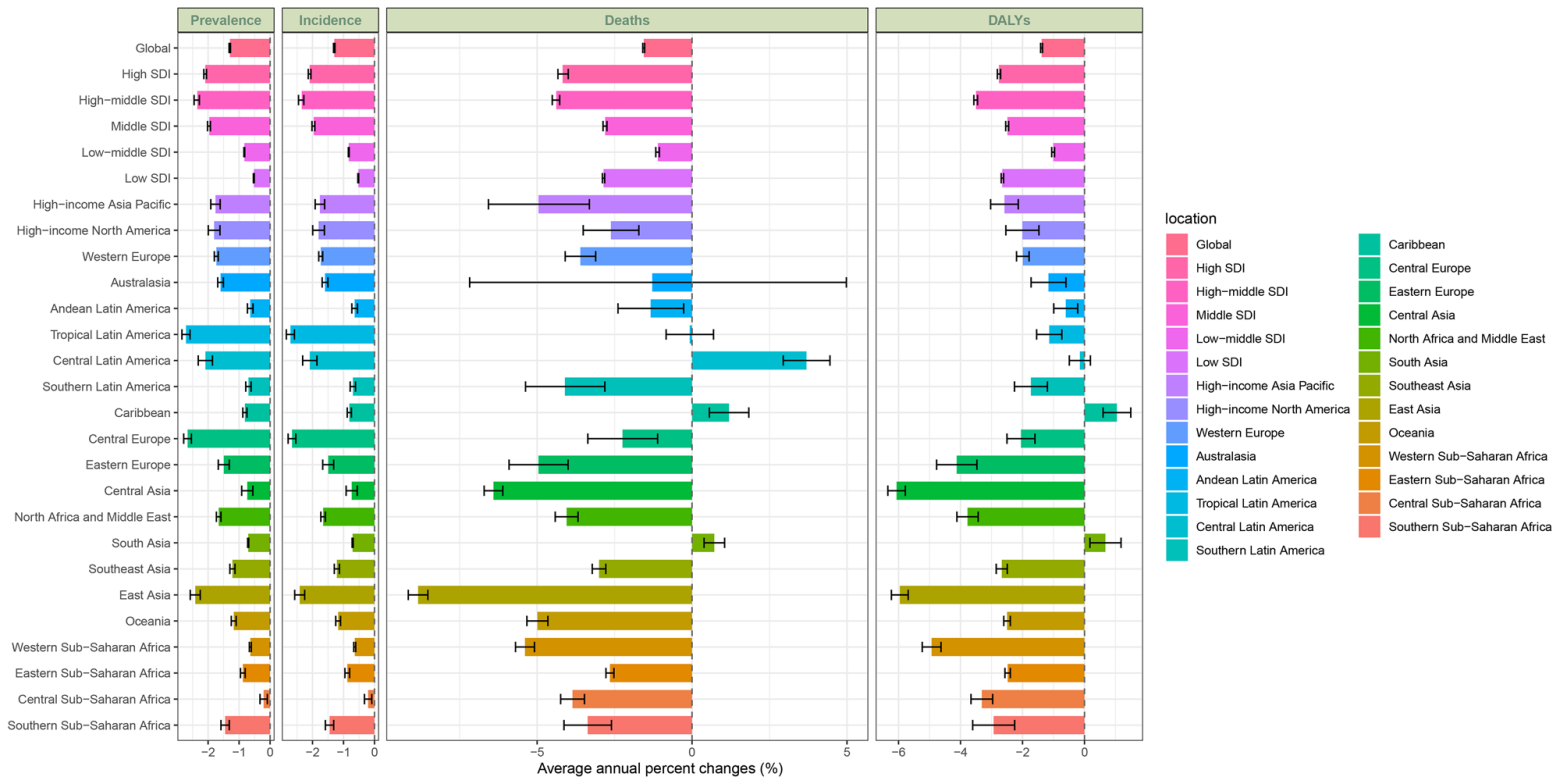
Global, SDI and regional AAPC of acute hepatitis B burden in WCBA (1990–2021). (DALYs = disability-adjusted life years; AAPC = average annual percent change, SDI = Socio-demographic Index, AHB = acute hepatitis B, WCBA = women of childbearing age)

**Fig. 8:**
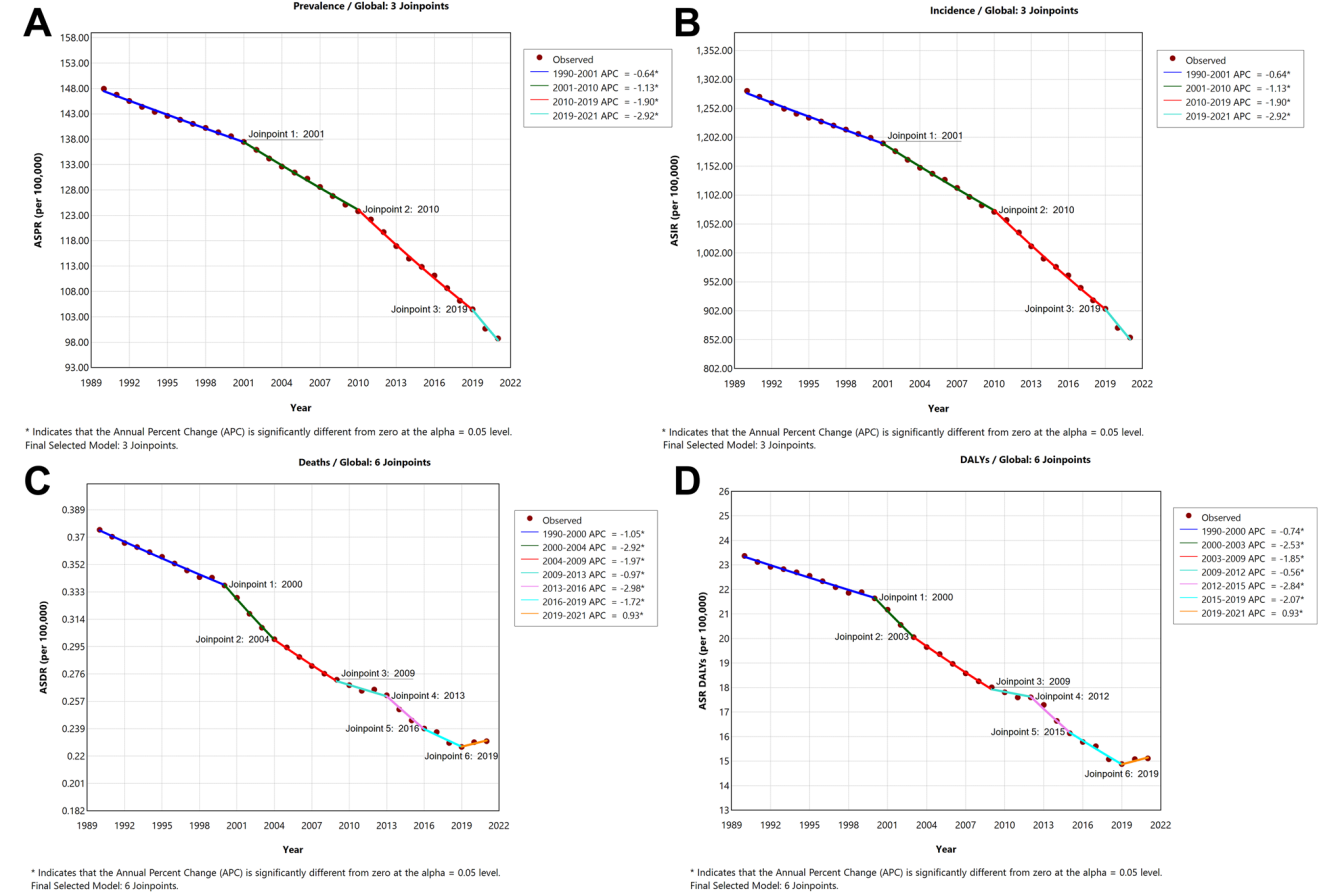
Joinpoint regression analysis of acute hepatitis B burden in WCBA from 1990 to 2021. (**A**: ASPR, **B**: ASIR, **C**: ASMR, **D**: ASDR; ASPR = age-standardized rate of prevalence, ASIR = age-standardized rate of incidence, ASMR = age-standardized rate of mortality, ASDR = age-standardized rate of disability-adjusted life years, WCBA = women of childbearing age)

In terms of time distribution, the ASIR and ASPR of acute hepatitis B showed a continuous downward trend from 1990 to 2021, which exhibited a similar slope distribution, with AAPCs of −1.297% (95% CI: −1.322% to −1.277%) and −1.297% (95% CI: −1.322% to −1.277%), respectively (Fig. 7, Additional file 2: Table S5, Fig. 8). The trends can be divided into four decreasing periods: 1990–2001, 2001–2010, 2010–2019 and 2019–2021. In these four periods, the speed of decrease gradually increased over time. The ASIR and ASPR of acute hepatitis B decreased most significantly from 2019 to 2021, with APCs of −2.916% per year (95% CI: −3.331% to −2.402%) and −2.916% per year (95% CI: −3.331% to −2.402%), respectively.

Comparatively, for the global ASMR and ASDR of acute hepatitis B, the trend declined consistently prior to 2019 which was followed by a slight increase from 2019 to 2021, with APCs of 0.932% (95% CI: 0.312% to 1.446%) and 0.928% (95% CI: 0.296% to 1.405%), respectively. Overall, the ASMR and ASDR of acute hepatitis B patients still declined from 1990 to 2021, with AAPCs of −1.559% (95% CI: −1.59% to −1.531%) and −1.383% (95% CI: −1.415% to −1.354%), respectively. The ASMR experienced the greatest reduction from 2013 to 2016, with an APC of −2.979% per year (95% CI: −3.273%, −1.13%). Similarly, the most significant decrease in the ASDR occurred from 2012 to 2015, with an APC of −2.837% per year (95% CI: −3.096%, −0.829%).

### Future forecasts of the global burden of acute hepatitis B

The global burden of acute hepatitis B was projected to decrease from 2021 to 2050, with the same decreasing trends across different measures. Overall, both the observed and predicted results indicate a decline in all four measures. The incidence and prevalence rates show similar downward trends, as do the death rates and DALYs rates; however, the latter decline less steeply than the former.

**Fig. 9:**
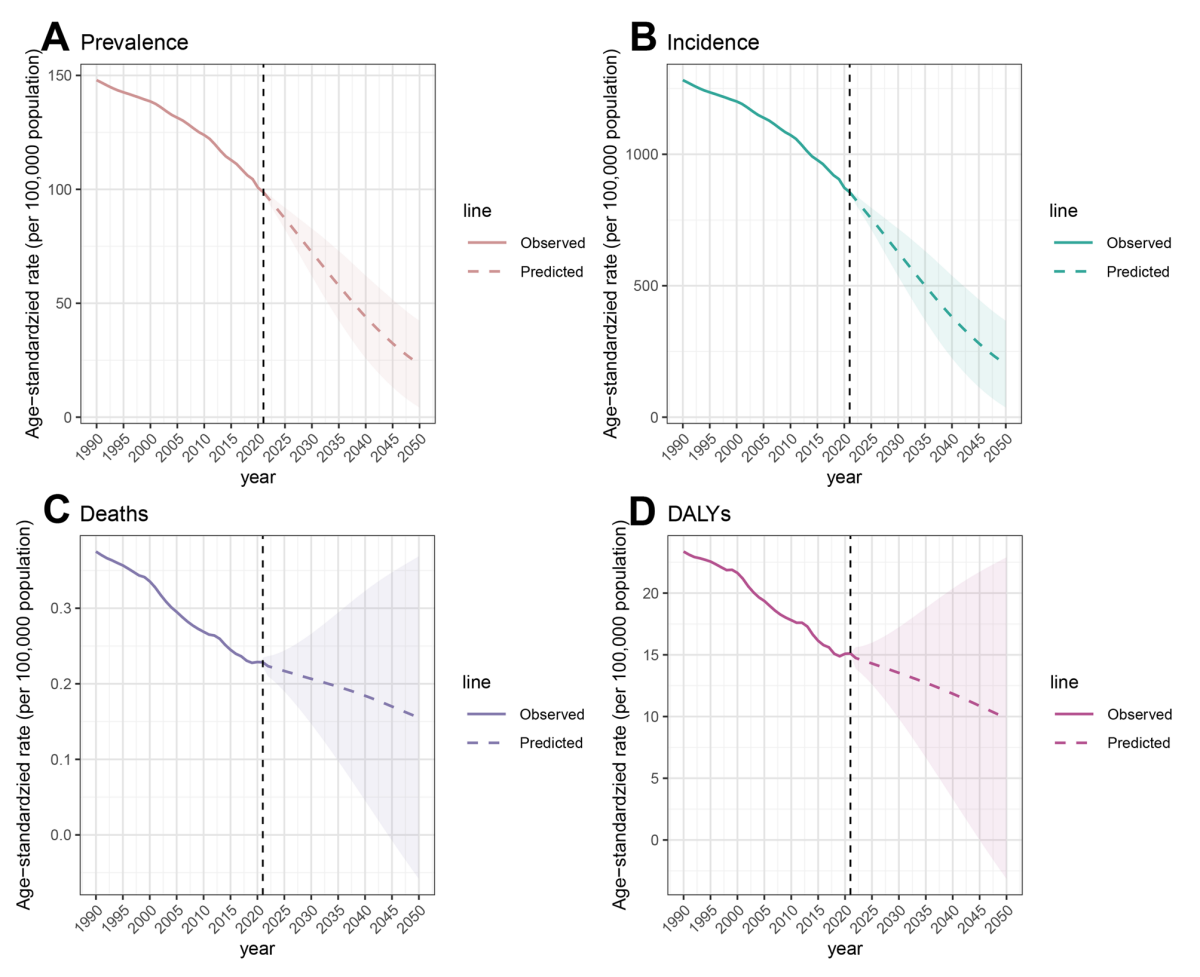
Future forecasts of the global burden of acute hepatitis B ASRs in WCBA to 2050. (**A**: Prevalence, **B**: Incidence, **C**: Deaths, **D**: DALYs; ASRs = age-standardized rates, WCBA = women of childbearing age, DALYs = disability-adjusted life years)

The ASPR of acute hepatitis B is expected to decrease globally, from approximately 98.7 per 100,000 people in 2021 to approximately 23.3 per 100,000 people by 2050, representing approximately 76.4% decrease over three decades (Fig. 9). The global incidence of acute hepatitis B is on a downward declining, with the ASIR expected to fall from approximately 855.8 per 100,000 people in 2021 to about 201.9 per 100,000 people by 2050. The ASMR for acute hepatitis B is projected to decline significantly, from about 0.228 per 100,000 people in 2021 to approximately 0.155 per 100,000 people by 2050. The ASDR for acute hepatitis B is expected to plummet, with a significant decrease from 15.1 per 100,000 people in 2021 to approximately 9.9 per 100,000 people by 2050.

## Discussion

The findings of this study provide a comprehensive assessment of the global burden and long-term trends of acute hepatitis B (AHB) among women of childbearing age (WCBA) from 1990 to 2021, with projections extending to 2050. Overall, age-standardized incidence, prevalence, mortality, and DALY rates showed sustained declines over the past three decades, indicating substantial global progress in hepatitis B control. These encouraging trends are broadly consistent with the expansion of hepatitis B vaccination programs, prevention of mother-to-child transmission strategies, and improvements in infection control and blood safety worldwide [4, 28].

However, these improvements were not evenly distributed. Pronounced disparities persisted across socio-demographic settings, with low-SDI regions continuing to experience disproportionately high burdens and slower rates of decline. These findings suggest that while global hepatitis B prevention efforts have yielded measurable benefits, structural inequalities in healthcare access, vaccination coverage, and disease control capacity remain major barriers to achieving equitable and sustained reductions in AHB burden.

### Socio-demographic inequalities in AHB burden

Regional variations in acute hepatitis B burden exhibit substantial disparities, demonstrating a strong correlation with the Socio-demographic Index (SDI). When comparing ASPR and ASIR across SDI regions in Fig. 1, a seemingly paradoxical pattern emerges: high SD regions have higher ASPR/ASIR (especially high-middle SDI regions exhibit the second-highest burden). This might be due to the improvement in screening scope and accuracy. High SDI countries (such as the United States) might discover more cases through universal screening, resulting in reported ASPR/ASIR being higher [44]. In 2021, the ASRs in low SDI regions were markedly lower than those in high SDI regions, with slower decline trajectories indicating structural divides in healthcare accessibility [45, 46]. This difference stems mainly from the interaction of multiple factors. Mother-to-child transmission is an important source of hepatitis B transmission and is generally prevented by vaccination [47]. However, WHO estimates the hepatitis B (HepB3) vaccination coverage in the African Region at merely 17%, highlighting a stark disparity with the 79% reported in the Western Pacific Region. This disparity is particularly pronounced in African countries with weak cold chain systems, such as Zimbabwe (ASPR 430.9/100,000) [48–51]. Structural constraints in low and middle-income countries limit resource and technology access, while their fragile health systems delay case detection and treatment, thereby exacerbating disease transmission dynamics [52]. Although transmission routes were not directly assessed in the GBD dataset, existing epidemiological evidence indicates that acute hepatitis B in adults is mainly transmitted through sexual contact, injecting drug use, and healthcare-associated exposures [18, 53–55]. The relative importance of these routes varies across regions. In high-income regions, surveillance data suggest that sexual transmission and injecting drug use account for a substantial proportion of acute HBV infections [53, 54], whereas in some low-SDI settings unsafe medical practices and limited infection control remain important contributors [53, 55]. These contextual differences in transmission patterns may partly help explain the persistent regional disparities observed in our analysis. Notably, The regions represented by Somalia and Afghanistan are prone to perennial conflict, which can lead to medical paralysis, thus further limiting service accessibility [56]. Cross-regional population movement between areas with differing hepatitis B prevalence may partially contribute to observed epidemiological changes, although causality cannot be inferred from the present analysis [57].

### Age-related vulnerability within women of childbearing age

In terms of age structure, the burden of acute hepatitis B disease also significantly differed by age, with a distinct age gradient effect within women of childbearing age. Notably, women with HBV DNA > 200,000 IU/mL are at higher risk of vertical transmission, and women of childbearing age are more likely to develop chronic hepatitis B because pregnancy may induce an immune-tolerant state [58]. Although AHB is typically transient, it can manifest with high HBV DNA levels during acute viremia, which needs anti-viral intervention to prevent mother-to-child transmission [18, 19]. The observed peak incidence of acute hepatitis B among women aged 25–29 years may partly reflect increased opportunities for diagnosis rather than a true increase in infection risk. Previous studies have shown that routine health examinations, including pregnancy-related screening and other standardized medical assessments in early adulthood, substantially increase the likelihood of hepatitis B testing and diagnosis among women, thereby influencing age-specific incidence patterns [10]. In the low SDI region, women aged 20–29 years accounted for 40% of all cases, whereas in the high SDI region, the incidence was lower in all age groups and the gap was small, revealing differences in the public health system and health promotion strategies behind the two. This age range also coincides with the typical age at first birth in some countries and regions, which commonly falls in the mid-to-late twenties [59]. Increased healthcare contact related to pregnancy may therefore contribute to higher opportunities for hepatitis B testing and diagnosis in this age group. Furthermore, the age-period-cohort analysis also revealed that the most rapid decline in incidence occurred in the 15–19-year age group, with the rate of decline diminishing progressively with age, and in regions with a higher SDI, the rate of decline varies more significantly among different age groups. (Fig. 4 A) An important demographic perspective to consider is the increasing age at first birth in high-SDI regions, which may modify both the age distribution of reproductive health service utilization and the broader social and behavioral contexts of HBV exposure [10, 59]. The more pronounced decline among younger women in high-SDI regions likely reflects the long-term cumulative impact of universal infant vaccination programs and improved infection control practices, which would preferentially benefit cohorts entering adolescence and early adulthood in recent decades [60].

In addition, the potential confounding effect of HIV or HCV coinfection should be considered. Such coinfections can accelerate liver disease progression, affecting mortality and DALY estimates. They may also lead to more frequent health encounters and testing, possibly creating surveillance bias in reported AHB Incidence among some groups. While our analysis cannot cover this directly, it remains an important background factor for interpreting age-specific patterns [61–63]. What’s more, the screening strategy chosen is also important: point-of-care rapid diagnostic tests (RDTs) overcome the access barriers of conventional lab testing. A prime example is a novel hepatitis B core-related antigen (HBcrAg) RDT, which accurately identifies high-viral-load pregnant women, with same-day results, solving the critical bottleneck of diagnostic delay [64, 65]. Thus, the wider adoption of such rapid tests facilitates earlier detection and likely elevates the reported incidence in this age group.

In the broader epidemiological context, men of reproductive age consistently bear a higher burden of acute hepatitis B than women [66]. For instance, U.S. surveillance data show a higher acute HBV incidence in men, a pattern likely reflecting higher engagement in risk behaviors such as injection drug use and multiple sexual partners, which together account for approximately half of all reported cases with identifiable risk factors [67].

### Future projections and implications for hepatitis B elimination

In the past, the Joinpoint analysis identified four distinct segments (1990–2001, 2001–2010, 2010–2019, 2019–2021), during which the rate of decline in ASIR accelerated progressively. This pattern of non-linear acceleration in decline temporally overlaps with major global and regional milestones in hepatitis B prevention [4, 68–71]. However, as a descriptive analysis, this study cannot attribute these trend changes to specific policies or interventions.

The model predicted that the four indicators of the global WCBA burden of hepatitis B will decline in varying amounts over time and by region from 2021 to 2050, with the ASIR in particular declining by 76.4%, showing that prevention and treatment initiatives are making the elimination of viral hepatitis possible, but that continuous investment in viral hepatitis services and strengthening of the wider health system are still needed if the WHO 2030 targets are to be met [4, 22, 72]. It is critical to emphasize that these projections are model-based extrapolations, not deterministic forecasts. They illustrate a potential trajectory contingent on the continuation of current public health efforts and in the absence of major disruptions or accelerated interventions. Therefore, they do not guarantee the achievement of elimination targets but rather underscore the need for sustained and accelerated action, particularly in low-resource settings.

Among these prevention measures, hepatitis B vaccination remains a cost-effective, population-wide intervention [73, 74]. Since the introduction of the hepatitis B vaccine into national immunization programs by the World Health Organization (WHO) in 1992, the steady increase in vaccination coverage has played a key role in curbing the spread of AHB [28, 73]. Although hepatitis B vaccination is offered free of charge in 191 countries, important gaps remain: global coverage of the timely birth dose is only 46%, with marked regional disparities (17% in sub-Saharan Africa and 79% in the Western Pacific), while three-dose coverage ranges from 72% in sub-Saharan Africa to > 93% in East Asia [74, 75]. These data underscore that optimisation efforts should focus on improving regional equity rather than simply extending free-policy status. In addition to sustaining high vaccination coverage, prevention strategies should also address the dominant transmission routes in different settings. In higher-SDI regions, strengthening sexual health services, targeted screening, and harm reduction programs for people who inject drugs may further reduce AHB incidence. In low-SDI regions, improving infection control in healthcare facilities, ensuring safe injection practices, and enhancing blood safety remain essential [55]. Tailoring interventions to local transmission patterns may help accelerate progress toward hepatitis B elimination goals. Additionally, the inclusion of AHB screening in universal health coverage and the lowering of antiviral prices through patent exemptions could also lead to an increase in targeted treatment coverage, suggesting that we need to focus on increasing testing and treatment rates in the female population of childbearing age by optimizing efficacy and price accessibility [58, 76, 77].

### Strengths

The innovation of this study is that it focuses on the global WCBA and provides a multidimensional and systematic framework for analyzing AHB. This study combines global, regional, and national data and uses the age-period-cohort model to analyze the spatial and temporal characteristics of AHB, clearly revealing the differences in disease burden among different SDI regions, age groups, and birth cohorts, and providing a basis for precise prevention and control. We also classified the stages of decline through linkage regression analysis, which provided quantitative support for the assessment of the timeliness of prevention and control strategies. Importantly, the prediction of future trends indicates that the burden of AHB will continue to decline from 2021 to 2050, which provides a forward-looking reference for global prevention and control planning.

### Limitations

However, there are limitations to this study. At the data level, poor medical records and limited diagnostic capacity in some low SDI countries may lead to underreporting of cases, affecting the accuracy of incidence rates. The reliance on GBD modeling data may result in biased estimations of age, period and cohort effects due to uncertainty in model assumptions and parameter settings. This study is based on retrospective analysis of historical data, which makes reflecting emergencies such as new outbreaks and mutated strains in real time difficult. Furthermore, the targeted analyses of special populations (e.g., pregnant women and immunodeficient persons) limit the in-depth exploration of prevention and control strategies for the general population. Subsequent studies can further optimize the conclusions of the analysis by improving the data monitoring system and combining it with real-time epidemiological investigations.

## Conclusions

The global burden of acute hepatitis B among women of childbearing age has a declining trajectory, with this positive trend being consistent with the widespread implementation of vaccination and mother-to-child transmission interruption strategies. Concurrently, significant geographical heterogeneity exists in disease burden, where limited healthcare accessibility and fragile health systems exacerbate transmission risks in low-development regions. Overall, modeling projections indicate that continued declines may contribute to progress toward the WHO viral hepatitis elimination goals, although current projections alone are insufficient to indicate full elimination. Achieving these goals will require sustained efforts. In particular, higher vaccine coverage, safe injection and sufficient prevention of mother-to-child transmission remain essential.

## Electronic supplementary material

Below is the link to the electronic supplementary material.

Supplementary Material 1: Additional files 1. Contains Supplemental Methods used throughout the study

Supplementary Material 2: Additional files 2. Contains supplemental tables used throughout the study

## Data Availability

The datasets supporting the conclusions of this article are available in the GBD repository, https://vizhub.healthdata.org/gbd-results/.

## Abbreviations

AHB: Acute hepatitis B
WCBA: Women of childbearing age
DALYs: Disability-Adjusted Life Years
ASRs: Age-standardized rates
ASIR: Age-standardized rate of Incidence
ASPR: Age-standardized rate of Prevalence
ASMR: Age-standardized rate of Mortality
ASDR: Age-standardized rate of disability-adjusted life years
EAPC: Estimated Annual Percentage Change
APC model: Age-period-cohort model
BAPC: Bayesian age-period-cohort
APC: Annual percent change
AAPC: Average annual percent change
GBD: Global Burden of Disease
GHDx: Global Health Data Exchange
IHME: Institute for Health Metrics and Evaluation
SDI: Socio-Demographic Index
UI: Uncertainty Interval
CI: Confidence Interval
