# Supplementary material for: Global burden and forecast of acute viral hepatitis B among women of childbearing age: a systematic analysis of the global burden of disease study 2021
Source: BMC Infect Dis. 2026 May 16;26:1056. doi: 10.1186/s12879-026-13302-w (PMC13227652; doi:10.1186/s12879-026-13302-w)
Supplement: Supplementary file 1 — Supplementary Material 1: Additional files 1. Contains Supplemental Methods used throughout the study [file 12879_2026_13302_MOESM1_ESM.docx]

**Additional file 1**

This supplemental material has been provided by the authors to give readers additional information about their work.

**CONTENTS**

1. Supplemental Methods2

1.1 Age-standardized rate (ASR)2

1.2 Estimated Annual Percentage Change (EAPC)2

1.3 Frontier analysis2

1.4 Smoothing spline models3

1.5 Joinpoint Regression Analysis3

1.6 Age-period-cohort model analysis4

1.7 The Bayesian age-period-cohort (BAPC) Model Projection5

2. Explanation of the Data Used6

2.1 Data sources6

2.2 Case definitions7

**1. Supplemental Methods**

**1.1 Age-standardized rate (ASR)**

The ASRs were calculated using the world population age standard in the GBD Study 2021 as the reference used for standardization, which serves as the benchmark for all age groups.[1] We calculated the age-standardized rate in women aged from 15 to 49 years following this formula:

$$\mathrm{ASR}= \frac{\sum_{i=1}^{A} a_{i}w_{i}}{\sum_{i=1}^{A} w_{i}} \times100,000$$

(𝑎_𝑖_: age-specific rate of the 𝑖^th^ age group; *w* _𝑖_: number of the corresponding 𝑖^th^ age group in the standard population; *A*: number of age groups.)

**1.2 Estimated Annual Percentage Change (EAPC)**

The equation used was: Y = α+βX+ε, where Y represents the natural logarithm of the ASR, X represents the calendar year, α is the intercept, β is the slope or trend, and ε is the error term. The linear regression model is used to calculate the 95% confidence interval (CI) of the EAPC. To assess the overall trends of AHB among women of reproductive age, EAPC was calculated using the formula: 100 × [exp(β) -1].

If both the EAPC and its 95% CI lower limit are positive, it indicates an upward trend in the ASR. Conversely, if both the EAPC and its 95% CI upper limit are negative, it indicates a downward trend in the ASR. If neither condition is met, the age-standardized rate is considered stable.

**1.3 Frontier analysis**

Frontier analysis was utilized to capture the nonlinear relationship between SDI and the age-standardized DALYs rate. To generate a non-linear frontier, locally weighted regression (LOESS) combined with local polynomial regression was applied, using smoothing spans of 0.3 to create smooth frontier lines and 1,000 bootstrap samples to ensure the robustness of the analysis.

The absolute distance between a country's DALYs rate and its frontier, termed the effective difference, reflects the unrealized health gains and improvement potential that exist, based on the country or region's current development level. Through frontier analysis, the countries and territories exhibiting the most pronounced disparities have been identified, including: (1) the 5 regions with the smallest gap to frontier among all regions; (2) the 5 high SDI (>0.85) regions with the largest gap to frontier values.

**1.4 Smoothing spline models** [2, 3]

Smoothing spline models were employed to explore the relationship between AHB burdens among WCBA and SDI levels. Smooth splines were fitted using the Locally Weighted Scatterplot Smoothing method. Additionally, Spearman correlation analysis was utilized to estimate the ρ indices and p values, thereby measuring the association between the age-standardized rate and SDI for each region and country.

**1.5 Joinpoint Regression Analysis**

The Joinpoint regression model is a collection of linear statistical models that were used to identify significant changes in data trends over time, distinguishing real shifts in trends from random variability.[4] In the analysis, compared to linear transformation (𝑦=𝑥b), log transformation (𝑙𝑛(𝑦)=𝑥b) is more suitable for epidemiological data, which was selected in this study.[5] Additionally, the grid search method (GSM) is utilized to calculate all possible join points, and in the heteroscedastic error option, the standard error (provided) is selected. Building on this foundation, the Monte Carlo permutation test was employed to determine the optimal number of join points, with a maximum of 6 and a minimum of 0. Finally, this model computed the annual percentage change (APC), average annual percentage change (AAPC), and the corresponding 95% confidence intervals (CI) to quantify trend changes from 1990 to 2021. Previous studies have detailed the calculation methods for standard error (SE), annual percent change (APC), and average annual percent change (AAPC). [5] The Joinpoint regression program software and computing formulas of APC & AAPC can be obtained on their website. [5, 6]

The Annual Percent Change (APC) measures the trend (increase or decrease) within each specific segment of the fitted Joinpoint model. It represents a constant, localized rate of change for each period.

The Average Annual Percent Change (AAPC) provides a single, summary measure of the trend over the entire pre-specified time interval (e.g., 1990–2021). It is calculated as a weighted average of the APCs from all segments within that interval, offering an overall trend estimate that is comparable even if the underlying segmented models differ between regions or groups.

**1.6 Age-period-cohort model analysis**

The Age-period-cohort model is used to evaluate the associations between age, period, and birth cohort in AHB incidence and DALYs. An individual’s birth cohort is calculated by the time period of death and the individual’s death age (i.e., birth cohort = period - age). Net drift represents the overall log-linear trend by period and birth cohort and indicates the overall annual percentage change of the expected age-adjusted rates over time. Local drift represents the log-linear trend by period and birth cohort for each age group and indicates the annual percentage change of the expected age-specific rates over time. The longitudinal age curve indicates the expected age-specific rate in a reference cohort adjusted for period effects. The period relative risks are the ratio of age-specific rates in each period relative to the reference period, representing changes over time periods that affect all age groups simultaneously, presumably arising from changes in social, cultural, economic, or physical environments. Cohort effects are associated with changes across groups of individuals with the same birth years, and Cohort relative risks are the ratio of the age-specific rate in each cohort relative to a reference cohort.

The WCBA range of 15-49 years was segmented into seven successive 5-year age intervals for further evaluation: 15-19, 20-24, 25-29, 30-34, 35-39, 40-44, and 45-49 years. The study's timeframe spanning from 1992 to 2021(data from 1990-1991 were not considered because there were insufficient years for a complete 5-year period) was divided into six consecutive 5-year periods: 1992-1996, 1997-2001, 2002-2006, 2007-2011, 2012-2016, and 2017-2021, with 1992 to 1996 survey year as the reference period group. Thus, the analysis considered 12 partially overlapping 10-year birth cohorts, ranging from 1942 to 1951 (median, 1947) to 1997 to 2006 (median, 2002), with the birth cohort of 1972 to 1981 (median, 1977) as the reference group.

APC model analyses were conducted by the age-period-cohort Web Tool provided by the National Cancer Institute. [7] The Wald χ2 test was adopted to test the significance of the estimable parameters and functions. Detailed methods can be found in the introduction section of the age-period-cohort Web Tool and the related book on the APC model. [8]

**1.7 The Bayesian age-period-cohort (BAPC) Model Projection**

This study utilized the Bayesian age-period-cohort (BAPC) model incorporating integrated nested Laplace approximations (INLA) to project future trends in AHB burden from 2022 to 2050. The computational process was executed using the R-packages BAPC and INLA, leveraging data from GBD 2021 and demographic projections by the IHME, while adhering to protocols established in prior studies.[9]This methodology facilitates nuanced projections of future disease burdens, taking into account the intricate interplay of age, period, and cohort effects.

**2. Explanation of the Data Used**

**2.1 Data sources**

The 2021 Global Burden of Disease (GBD) report, compiled by the Institute for Health Metrics and Evaluation (IHME), is an international collaborative research project that provides comprehensive epidemiological data on 371 diseases and injuries across 204 countries and regions, covering 811 subnational locations, along with 88 attributable risk factors. [10]

The burden of AHB was estimated using Bayesian meta-regression models (DisMod-MR 2.1), which accounted for location, age, sex, year, and pathogen. Detailed information on the estimated burden of AHB, including data input, processing, and modeling methods has been described in previous studies.[11] All data can be accessed through GBD 2021, including previously reported data, statistical modeling, and methodological information. [12, 13] Therefore, no ethical approval or informed consent is required for this study.

This study utilized the Global Health Data Exchange (GHDx) query tool[11, 14] to collect annual data on acute hepatitis B among women of childbearing age (15–49 years) from 1990 to 2021 across 204 countries and regions. These regions were categorized into 21 GBD areas based on epidemiological similarity and geographical location. Furthermore, these areas and countries were further divided into five distinct quintiles via the Socio-Demographic Index (SDI): Low SDI, Low to Medium SDI, Medium SDI, High to Medium SDI, and High SDI. [12] The study focused on women aged 15 to 49, divided into seven GBD age groups with a 5-year interval: 15-19 years, 20-24 years, 25-29 years, 30-34 years, 35-39 years, 40-44 years, and 45-49 years.

**2.2 Case definitions**

Case Definitions in source data (e.g., vital registration, hospital records) is fundamentally linked to specific ICD codes, which are then mapped to the GBD cause list. The ICD-10 codes mapped to acute hepatitis B within the GBD framework primarily include codes corresponding to acute HBV infection (e.g., B16 series). Detailed cause mapping is described in the GBD 2021 methodology. Other HBV-related conditions, including **chronic hepatitis B**, **cirrhosis**, **liver cancer**, and **hepatitis B (overall),** each correspond to distinct ICD-10 code ranges. [11]

Case Definitions for ‘Acute Hepatitis B’ in each of the four GBD measures are explicitly and narrowly defined around the initial, short-term infection event of HBV:

**Acute hepatitis B**: A new hepatitis B virus (HBV) infection event and the individual in the initial acute phase of infection (typically ≤ 6 months), irrespective of clinical symptoms. This is distinct from chronic HBV infection (long-term HBsAg carriage).

**Incidence**: The number of new Acute Hepatitis B virus (HBV) infections occurring in given years.

**Prevalence**: The number of individuals experiencing the acute phase of HBV infection (typically ≤ 6 months) at a point in time, regardless of clinical symptoms.

**Mortality**: Only deaths directly and causally due to the AHB, predominantly fulminant hepatic failure. [15] This number is exceedingly rare. Consequently, the ASMR for acute hepatitis B is very low.

**DALYs (**Disability**-Adjusted Life Years)**: The sum of:

**YLLs (Years of Life Lost)**: From the aforementioned mortality due to the AHB (minimal contribution).

**YLDs (Years Lived with Disability)**: Calculated from the prevalence of acute cases and a disability weight assigned to the short-term symptoms of acute hepatitis (e.g., jaundice, fatigue, abdominal pain).

**References**

1. GBD 2021 Demographics Collaborators. Global age-sex-specific mortality, life expectancy, and population estimates in 204 countries and territories and 811 subnational locations, 1950-2021, and the impact of the COVID-19 pandemic: a comprehensive demographic analysis for the Global Burden of Disease Study 2021. Lancet. 2024;403:1989–2056. https://doi.org/10.1016/S0140-6736(24)00476-8.

2. Bao Y, Li Y, Zhou Y, Qiang N, Li T, Zhang Y, et al. Global burden associated with rare infectious diseases of poverty in 2021: findings from the Global Burden of Disease Study 2021. Infect Dis Poverty. 2024;13:85. https://doi.org/10.1186/s40249-024-01249-6.

3. Cleveland WS. Robust Locally Weighted Regression and Smoothing Scatterplots. Journal of the American Statistical Association. 1979;74:829–36. https://doi.org/10.2307/2286407.

4. Kim HJ, Fay MP, Feuer EJ, Midthune DN. Permutation tests for joinpoint regression with applications to cancer rates. Stat Med. 2000;19:335–51. https://doi.org/10.1002/(sici)1097-0258(20000215)19:3<335::aid-sim336>3.0.co;2-z.

5. Li HZ, Du LB. [Application of Joinpoint regression model in cancer epidemiological time trend analysis]. Zhonghua Yu Fang Yi Xue Za Zhi. 2020;54:908–12. https://doi.org/10.3760/cma.j.cn112150-20200616-00889.

6. Joinpoint Regression Program. https://surveillance.cancer.gov/joinpoint/index.html. Accessed 27 Jun 2025.

7. Rosenberg PS, Check DP, Anderson WF. A web tool for age-period-cohort analysis of cancer incidence and mortality rates. Cancer Epidemiol Biomarkers Prev. 2014;23:2296–302. https://doi.org/10.1158/1055-9965.EPI-14-0300.

8. Yang Y, Land KC. Age-Period-Cohort Analysis: New Models, Methods, and Empirical Applications. 1st edition. New York: Chapman and Hall/CRC; 2016. https://doi.org/10.1201/b13902.

9. Knoll M, Furkel J, Debus J, Abdollahi A, Karch A, Stock C. An R package for an integrated evaluation of statistical approaches to cancer incidence projection. BMC Med Res Methodol. 2020;20:257. https://doi.org/10.1186/s12874-020-01133-5.

10. GBD 2019 Diseases and Injuries Collaborators. Global burden of 369 diseases and injuries in 204 countries and territories, 1990-2019: a systematic analysis for the Global Burden of Disease Study 2019. Lancet. 2020;396:1204–22. https://doi.org/10.1016/S0140-6736(20)30925-9.

11. GBD 2021 Diseases and Injuries Collaborators. Global incidence, prevalence, years lived with disability (YLDs), disability-adjusted life-years (DALYs), and healthy life expectancy (HALE) for 371 diseases and injuries in 204 countries and territories and 811 subnational locations, 1990-2021: a systematic analysis for the Global Burden of Disease Study 2021. Lancet. 2024;403:2133–61. https://doi.org/10.1016/S0140-6736(24)00757-8.

12. Global Health Data Exchange | GHDx. https://ghdx.healthdata.org/. Accessed 28 Apr 2025.

13. Global Burden of Disease Study 2021 (GBD 2021) Sources Tool | GHDx. https://ghdx.healthdata.org/gbd-2021/sources. Accessed 27 Apr 2025.

14. GBD Results. Institute for Health Metrics and Evaluation. https://vizhub.healthdata.org/gbd-results. Accessed 28 Apr 2025.

15. GBD 2021 Causes of Death Collaborators. Global burden of 288 causes of death and life expectancy decomposition in 204 countries and territories and 811 subnational locations, 1990-2021: a systematic analysis for the Global Burden of Disease Study 2021. Lancet. 2024;403:2100–32. https://doi.org/10.1016/S0140-6736(24)00367-2.
