# Supplementary material for: Global burden and forecast of acute viral hepatitis B among women of childbearing age: a systematic analysis of the global burden of disease study 2021
Source: BMC Infect Dis. 2026 May 16;26:1056. doi: 10.1186/s12879-026-13302-w (PMC13227652; doi:10.1186/s12879-026-13302-w)
Supplement: Supplementary file 2 — Supplementary Material 2: Additional files 2. Contains supplemental tables used throughout the study [file 12879_2026_13302_MOESM2_ESM.docx]

**Additional file 2**

This supplemental material has been provided by the authors to give readers additional information about their work.

**CONTENTS**

Table S1. Prevalence cases and ASPR of AHB in 1990 and 2021 and its trends (National level)2

Table S2. Incidence cases and ASIR of AHB in 1990 and 2021 and its trends (National level)14

Table S3. Deaths cases and ASMR of AHB in 1990 and 2021 and its trends (National level)28

Table S4. DALYs and ASDR of AHB in 1990 and 2021 and its trends (National level).38

Table S5. Global, SDI and regional AAPC of AHB burden in WCBA (1990–2021)49

Table S6. Global, SDI and regional APC of AHB burden in WCBA (1990–2021)52

| **Table S1. Prevalence cases and ASPR of AHB in 1990 and 2021 and its trends (National level)** | | | | | |
| --- | --- | --- | --- | --- | --- |
| **location** | **Number (95% UI) 1990** | **ASR (95% UI) 1990** | **Number (95% UI) 2021** | **ASR (95% UI) 2021** | **EAPC (95%CI) 1990–2021** |
| American Samoa | 25.175 (13.107-45.622) | 208.458 (107.737-380.242) | 13.646 (6.971-25.879) | 117.706 (60.085-223.592) | -1.77 (-2.04 to -1.51) |
| Antigua and Barbuda | 4.153 (1.989-8.167) | 25.182 (11.97-49.687) | 3.941 (1.748-7.689) | 15.522 (6.914-30.246) | -1.48 (-1.76 to -1.2) |
| Arab Republic of Egypt | 22672.479 (10578.061-42593.654) | 173.571 (79.856-328.538) | 20508.698 (9572.606-39130.187) | 81.759 (38.073-155.982) | -2.09 (-2.4 to -1.78) |
| Argentine Republic | 1268.026 (682.906-2268.806) | 15.839 (8.518-28.372) | 1551.402 (826.225-2809.026) | 12.738 (6.79-23.055) | -0.5 (-0.66 to -0.34) |
| Australia | 3271.092 (1628.711-5986.757) | 73.194 (36.508-133.619) | 3130.063 (1524.41-5926.96) | 48.104 (23.555-90.814) | -1.28 (-1.53 to -1.03) |
| Barbados | 12.825 (5.337-26.052) | 18.495 (7.69-37.643) | 9.111 (3.764-19.027) | 12.551 (5.211-26.103) | -0.89 (-1.06 to -0.72) |
| Belize | 14.665 (7.163-27.636) | 34.279 (16.319-65.209) | 26.382 (12.017-51.88) | 22.607 (10.251-44.548) | -1.03 (-1.2 to -0.85) |
| Bermuda | 3.256 (1.473-6.571) | 18.957 (8.656-38.031) | 1.923 (0.852-3.764) | 13.92 (6.278-27.162) | -0.84 (-0.94 to -0.74) |
| Bolivarian Republic of Venezuela | 1139.863 (461.377-2420.43) | 22.8 (9.137-48.646) | 1062.293 (414.664-2248.659) | 15.385 (6.032-32.413) | -0.95 (-1.1 to -0.8) |
| Bosnia and Herzegovina | 665.573 (348.127-1210.787) | 56.881 (29.744-103.509) | 287.534 (140.566-548.03) | 37.95 (18.642-72.103) | -0.94 (-1.05 to -0.82) |
| Brunei Darussalam | 102.493 (49.936-193.807) | 149.056 (71.88-283.854) | 53.094 (24.292-101.859) | 40.385 (18.525-77.386) | -4.4 (-4.76 to -4.04) |
| Burkina Faso | 6892.67 (3464.669-12578.038) | 332.307 (165.174-610.962) | 13771.801 (7111.736-25351.269) | 263.991 (135.414-487.829) | -0.56 (-0.69 to -0.43) |
| Canada | 4042.693 (1948.151-7583.796) | 54.69 (26.589-101.957) | 3952.273 (1984.758-7365.539) | 47.353 (24-87.61) | -0.53 (-0.59 to -0.48) |
| Central African Republic | 1694.864 (726.109-3269.488) | 261.247 (110.594-508.906) | 3308.121 (1429.342-6445.442) | 237.819 (99.267-467.308) | -0.3 (-0.35 to -0.26) |
| Commonwealth of Dominica | 5.168 (2.43-10.044) | 29.819 (13.766-58.568) | 3.835 (1.721-7.538) | 23.377 (10.486-45.938) | -0.48 (-0.56 to -0.39) |
| Commonwealth of the Bahamas | 19.591 (8.328-38.428) | 26.469 (11.136-52.165) | 19.152 (8.278-38.601) | 17.722 (7.702-35.662) | -0.81 (-1 to -0.61) |
| Cook Islands | 5.684 (2.688-10.779) | 123.005 (57.523-234.892) | 2.083 (0.953-4.022) | 47.535 (21.803-92.032) | -2.99 (-3.33 to -2.65) |
| Czech Republic | 987.278 (444.732-1900.15) | 38.999 (17.771-74.503) | 533.056 (242.3-1062.536) | 21.415 (9.844-42.341) | -1.11 (-1.34 to -0.88) |
| Democratic People's Republic of Korea | 20559.969 (9489.104-39999.229) | 361.64 (166.291-707.015) | 19119.195 (8659.463-38022.403) | 283.911 (129.654-563.729) | -0.74 (-0.88 to -0.6) |
| Democratic Republic of Sao Tome and Principe | 62.241 (31.225-115.05) | 243.641 (120.164-453.623) | 106.323 (53.301-198.684) | 196.064 (97.647-367.805) | -0.63 (-0.82 to -0.44) |
| Democratic Republic of Timor-Leste | 282.724 (129.799-550.77) | 149.692 (68.171-292.971) | 449.704 (213.161-863.224) | 129.261 (60.338-251.533) | -0.39 (-0.47 to -0.32) |
| Democratic Republic of the Congo | 27354.219 (12951.909-52166.948) | 319.398 (149.002-617.474) | 65026.675 (32245.647-121980.279) | 306.253 (149.869-578.824) | -0.23 (-0.32 to -0.15) |
| Democratic Socialist Republic of Sri Lanka | 3461.889 (1826.668-6311.511) | 75.421 (39.675-137.953) | 3400.823 (1836.063-6171.985) | 59.806 (32.331-108.607) | -0.51 (-0.65 to -0.37) |
| Dominican Republic | 810.769 (396.44-1482.01) | 42.798 (20.794-78.959) | 818.744 (405.411-1549.183) | 28.371 (14.033-53.704) | -1.14 (-1.33 to -0.96) |
| Eastern Republic of Uruguay | 100.924 (51.794-187.806) | 13.45 (6.903-25.036) | 81.974 (41.839-152.228) | 9.527 (4.873-17.689) | -0.95 (-1.22 to -0.68) |
| Federal Democratic Republic of Ethiopia | 21662.508 (11104.517-39911.183) | 190.285 (96.135-353.45) | 37373.603 (19233.112-68831.723) | 133.929 (68.015-248.507) | -1.17 (-1.26 to -1.08) |
| Federal Democratic Republic of Nepal | 2886.344 (1653.729-5006.072) | 64.511 (36.752-112.68) | 4921.309 (2822.981-8618.248) | 55.076 (31.506-96.62) | -0.36 (-0.42 to -0.29) |
| Federal Republic of Germany | 3124.256 (1522.241-5865.646) | 16.009 (7.865-29.936) | 1812.543 (901.92-3392.015) | 9.578 (4.792-17.862) | -1.33 (-1.61 to -1.04) |
| Federal Republic of Nigeria | 74677.892 (39264.715-134987.088) | 367.468 (189.406-670.446) | 169359.674 (89816.185-309278.214) | 300.819 (157.484-554.169) | -0.66 (-0.72 to -0.59) |
| Federal Republic of Somalia | 5693.415 (2720.831-10956.391) | 333.388 (156.923-646.93) | 14671.94 (7030.469-27822.884) | 299.385 (141.367-574.072) | -0.37 (-0.4 to -0.34) |
| Federated States of Micronesia | 54.34 (28.146-98.067) | 233.671 (119.08-426.848) | 27.2 (13.73-50.784) | 110.137 (55.268-206.491) | -2.5 (-2.77 to -2.22) |
| Federative Republic of Brazil | 25909.334 (13463.08-46365.993) | 66.174 (34.147-118.921) | 17130.127 (7947.802-33867.723) | 27.756 (12.921-54.75) | -2.73 (-3.04 to -2.43) |
| French Republic | 3184.101 (1614.184-5935.595) | 21.979 (11.165-40.922) | 2424.447 (1211.35-4519.021) | 16.881 (8.493-31.351) | -0.65 (-0.74 to -0.55) |
| Gabonese Republic | 419.694 (195.328-804.797) | 186.183 (85.634-362.223) | 741.921 (365.14-1395.664) | 152.622 (74.53-289.224) | -0.51 (-0.6 to -0.43) |
| Georgia | 846.569 (421.556-1577.742) | 61.153 (30.526-113.953) | 388.683 (196.58-724.338) | 46.784 (23.867-86.852) | -0.71 (-0.85 to -0.56) |
| Grand Duchy of Luxembourg | 32.66 (15.631-61.767) | 33.446 (16.136-63.015) | 35.039 (16.124-67.334) | 20.014 (9.271-38.395) | -1.2 (-1.47 to -0.93) |
| Greenland | 9.927 (4.392-18.889) | 65.375 (29.065-124.63) | 7.461 (3.21-15.01) | 58.199 (25.064-116.793) | -0.21 (-0.25 to -0.17) |
| Grenada | 5.64 (2.528-11.248) | 28.23 (12.48-56.852) | 4.094 (1.621-8.564) | 15.994 (6.34-33.482) | -1.29 (-1.53 to -1.05) |
| Guam | 63.644 (34.69-113.88) | 181.178 (98.832-324.65) | 34.917 (18.442-64.204) | 95.025 (50.3-174.919) | -2.21 (-2.48 to -1.93) |
| Hashemite Kingdom of Jordan | 1087.806 (523.146-2048.06) | 131.228 (61.909-249.447) | 1902.282 (964.515-3595.032) | 66.282 (33.581-125.415) | -1.93 (-2.24 to -1.63) |
| Hellenic Republic | 2117.054 (1146.095-3827.893) | 83.945 (45.556-151.562) | 1223.338 (642.058-2258.05) | 50.355 (26.625-92.62) | -1.45 (-1.66 to -1.24) |
| Hungary | 1061.315 (550.984-1902.512) | 42.392 (22.184-75.624) | 790.18 (406.518-1462.292) | 38.086 (19.945-69.922) | -0.23 (-0.27 to -0.19) |
| Independent State of Papua New Guinea | 2764.339 (1243.155-5341.223) | 281.881 (123.927-548.889) | 5212.65 (2409.095-9995.163) | 201.999 (92.821-388.746) | -1.18 (-1.35 to -1.02) |
| Independent State of Samoa | 87.148 (44.802-161.682) | 238.585 (120.935-448.509) | 76.41 (39.138-142.634) | 161.876 (82.256-303.911) | -1.24 (-1.37 to -1.1) |
| Ireland | 233.271 (107.503-451.527) | 26.424 (12.138-51.23) | 276.871 (132.305-533.603) | 24.269 (11.744-46.444) | -0.34 (-0.37 to -0.3) |
| Islamic Republic of Afghanistan | 2275.018 (1299.405-3942.273) | 108.71 (61.295-190.621) | 6742.667 (3941.484-11595.55) | 98.862 (57.26-171.581) | -0.19 (-0.25 to -0.12) |
| Islamic Republic of Iran | 14929.618 (8326.932-26097.583) | 119.227 (65.735-210.77) | 12928.631 (7177.827-23436.311) | 47.45 (26.41-85.6) | -3.16 (-3.39 to -2.93) |
| Islamic Republic of Mauritania | 2007.384 (1030.857-3685.915) | 427.658 (217.285-792.868) | 3575.21 (1922.023-6473.728) | 344.781 (182.897-629.241) | -0.58 (-0.67 to -0.49) |
| Islamic Republic of Pakistan | 26339.206 (13473.1-48540.907) | 111.833 (56.278-207.776) | 47238.754 (23971.678-88257.152) | 79.306 (40.036-148.715) | -0.59 (-0.73 to -0.44) |
| Jamaica | 210.714 (95.14-418.743) | 34.183 (15.178-68.547) | 192.644 (80.23-399.476) | 24.268 (10.081-50.348) | -0.95 (-1.11 to -0.8) |
| Japan | 29934.563 (16666.955-53144.027) | 93.594 (52.287-165.365) | 18009.376 (9952.343-32326.206) | 72.885 (40.608-129.921) | -0.92 (-0.97 to -0.87) |
| Kingdom of Bahrain | 124.109 (69.059-218.353) | 106.999 (59.694-188.817) | 178.685 (99.92-314.633) | 52.296 (29.254-92.027) | -1.92 (-2.26 to -1.58) |
| Kingdom of Belgium | 530.975 (255.657-1000.006) | 21.781 (10.545-40.896) | 382.725 (178.528-743.36) | 14.678 (6.867-28.42) | -0.87 (-1.06 to -0.69) |
| Kingdom of Bhutan | 151.749 (73.59-285.48) | 107.02 (50.757-203.662) | 121.595 (60.295-226.336) | 58.403 (28.945-108.674) | -1.79 (-2.02 to -1.57) |
| Kingdom of Cambodia | 5605.562 (2473.539-11164.767) | 219.691 (95.884-442.084) | 6574.645 (2975.148-12987.963) | 143.168 (64.453-283.177) | -0.79 (-0.99 to -0.6) |
| Kingdom of Denmark | 354.405 (168.607-680.578) | 27.343 (13.103-52.214) | 351.129 (171.948-664.898) | 28.038 (13.872-52.9) | 0.09 (0.03 to 0.14) |
| Kingdom of Eswatini | 420.629 (199.261-785.556) | 214.498 (99.529-405.512) | 317.632 (156.454-596.369) | 107.716 (52.619-202.542) | -1.67 (-1.96 to -1.38) |
| Kingdom of Lesotho | 852.876 (413.172-1624.52) | 225.103 (107.875-430.8) | 775.649 (358.681-1487.849) | 155.791 (71.361-299.997) | -1 (-1.11 to -0.89) |
| Kingdom of Morocco | 3766.282 (2110.82-6538.281) | 60.832 (33.892-106.39) | 4430.876 (2508.746-7752.716) | 44.953 (25.465-78.623) | -0.71 (-0.86 to -0.56) |
| Kingdom of Norway | 270.158 (133.854-508.793) | 25.667 (12.782-48.234) | 217.777 (102.792-418.228) | 17.565 (8.326-33.602) | -0.99 (-1.19 to -0.79) |
| Kingdom of Saudi Arabia | 4173.182 (2093.07-7623.067) | 128.848 (63.771-237.511) | 5591.986 (2963.405-10234.907) | 50.11 (26.582-91.592) | -3.15 (-3.46 to -2.84) |
| Kingdom of Spain | 5149.531 (2752.627-9230.398) | 53.504 (28.6-95.991) | 2926.862 (1550.35-5369.391) | 24.017 (12.761-44.131) | -2.04 (-2.37 to -1.72) |
| Kingdom of Sweden | 387.203 (190.64-730.373) | 19.061 (9.485-35.826) | 396.217 (198.583-739.404) | 18.134 (9.171-33.671) | -0.22 (-0.25 to -0.2) |
| Kingdom of Thailand | 17661.923 (8655.747-32732.048) | 109.837 (53.661-204.3) | 11526.125 (5722.587-21614.005) | 63.275 (31.51-118.614) | -1.67 (-2.03 to -1.32) |
| Kingdom of Tonga | 52.726 (29.558-92.586) | 241.59 (133.981-426.666) | 24.872 (13.441-46.555) | 106.631 (57.553-199.809) | -3.13 (-3.55 to -2.7) |
| Kingdom of the Netherlands | 771.483 (401.557-1412.158) | 19.415 (10.141-35.41) | 640.972 (334.881-1155.877) | 17.395 (9.147-31.299) | -0.4 (-0.43 to -0.36) |
| Kyrgyz Republic | 1004.624 (504.047-1868.508) | 94.487 (46.916-176.782) | 1137.075 (570.61-2152.926) | 64.489 (32.266-122.39) | -0.87 (-1.01 to -0.72) |
| Lao People's Democratic Republic | 1349.786 (612.813-2659.532) | 137.539 (61.446-274.697) | 2133.56 (954.248-4160.553) | 106.607 (47.474-208.389) | -0.84 (-0.95 to -0.73) |
| Lebanese Republic | 596.505 (330.523-1050.447) | 80.319 (44.451-141.841) | 1008.224 (561.015-1781.941) | 62.566 (34.955-110.412) | -0.85 (-1.03 to -0.66) |
| Malaysia | 5123.712 (2857.671-9039.784) | 115.227 (64.047-204.142) | 4253.512 (2384.903-7556.469) | 51.592 (28.935-91.654) | -2.56 (-2.88 to -2.24) |
| Mongolia | 1515.145 (839.266-2690.059) | 298.995 (164.579-536.933) | 1436.896 (777.781-2591.804) | 156.75 (85.018-282.383) | -2.04 (-2.28 to -1.79) |
| Montenegro | 48.016 (21.383-94.463) | 30.659 (13.647-60.334) | 31.438 (14.4-62.596) | 21.506 (9.943-42.727) | -0.64 (-0.77 to -0.5) |
| New Zealand | 695.34 (337-1305.056) | 76.781 (37.241-144.053) | 395.73 (182.767-751.995) | 31.652 (14.699-60.018) | -2.91 (-3.12 to -2.69) |
| North Macedonia | 260.851 (135.1-479.187) | 51.187 (26.52-94.016) | 206.693 (106.75-380.883) | 37.31 (19.439-68.355) | -0.74 (-0.82 to -0.66) |
| Northern Mariana Islands | 36.641 (19.94-66.161) | 262.932 (143.054-475.472) | 17.191 (9.183-31.204) | 139.135 (74.55-252.785) | -2.02 (-2.3 to -1.73) |
| Palestine | 458.71 (251.546-818.259) | 105.469 (57.404-189.884) | 653.669 (351.496-1174.073) | 57.394 (30.824-103.363) | -1.99 (-2.37 to -1.61) |
| People's Democratic Republic of Algeria | 6516.14 (3592.218-11459.452) | 114.074 (62.455-202.615) | 10698.837 (5971.548-18748.41) | 92.13 (51.539-161.205) | -0.56 (-0.65 to -0.46) |
| People's Republic of Bangladesh | 24879.612 (13594.753-44383.997) | 103.007 (55.9-185.723) | 36348.436 (19642.986-65041.689) | 79.318 (42.809-142.128) | -0.52 (-0.64 to -0.41) |
| People's Republic of China | 849116.925 (431924.546-1549417.482) | 261.265 (131.759-478.747) | 424683.92 (200520.123-826366.042) | 122.972 (58.43-237.212) | -2.23 (-2.42 to -2.04) |
| Plurinational State of Bolivia | 485.605 (282.941-831.107) | 32.45 (18.819-56.028) | 778.403 (438.979-1374.798) | 25.249 (14.228-44.62) | -0.54 (-0.69 to -0.4) |
| Portuguese Republic | 831.524 (388.261-1619.138) | 33.01 (15.431-64.262) | 496.007 (223.657-965.716) | 19.803 (9.101-38.442) | -1.34 (-1.59 to -1.09) |
| Principality of Andorra | 5.731 (2.644-11.158) | 38.005 (17.619-73.795) | 5.412 (2.396-10.744) | 25.056 (11.233-49.468) | -1.29 (-1.49 to -1.09) |
| Principality of Monaco | 2.001 (0.975-3.817) | 29.093 (14.392-54.837) | 1.2 (0.568-2.336) | 14.532 (6.912-28.21) | -2.28 (-2.69 to -1.86) |
| Puerto Rico | 208.55 (93.351-405.406) | 21.786 (9.744-42.345) | 74.997 (35.168-143.494) | 9.322 (4.403-17.83) | -2.65 (-2.92 to -2.38) |
| Republic of Albania | 428.315 (229.587-768.657) | 51.189 (27.371-92.501) | 158.306 (83.215-293.594) | 24.772 (13.037-45.939) | -2.29 (-2.68 to -1.91) |
| Republic of Angola | 6777.098 (3101.69-13162.865) | 292.131 (132.542-573.52) | 18258.366 (8322.362-34946.291) | 241.798 (107.769-467.106) | -0.34 (-0.44 to -0.24) |
| Republic of Armenia | 639.999 (333.301-1166.47) | 73.058 (37.93-133.348) | 407.496 (215.514-766.465) | 49.638 (26.312-93.057) | -0.94 (-1.13 to -0.75) |
| Republic of Austria | 612.676 (288.581-1191.613) | 30.973 (14.667-60.165) | 472.326 (219.309-924.04) | 22.633 (10.568-44.182) | -0.8 (-0.96 to -0.64) |
| Republic of Azerbaijan | 2255.521 (1251.11-3997.875) | 120.157 (66.609-214.684) | 2925.912 (1602.758-5248.795) | 100.76 (55.395-180.154) | -0.45 (-0.56 to -0.35) |
| Republic of Belarus | 1420.45 (620.389-2821.502) | 55.66 (24.382-110.253) | 564.123 (241.736-1162.719) | 22.363 (9.678-45.835) | -2.11 (-2.47 to -1.76) |
| Republic of Benin | 1478.534 (820.885-2587.315) | 136.882 (75.617-241.767) | 3856.772 (2171.485-6761.571) | 125.729 (70.322-221.321) | -0.22 (-0.32 to -0.13) |
| Republic of Botswana | 640.635 (309.986-1217.869) | 196.666 (92.662-377.291) | 678.004 (308.87-1299.85) | 98.14 (44.677-188.443) | -2.35 (-2.61 to -2.08) |
| Republic of Bulgaria | 1982.592 (905.609-3871.962) | 97.247 (44.895-188.98) | 579.681 (255.038-1181.258) | 33.556 (14.905-67.765) | -2.79 (-3.17 to -2.41) |
| Republic of Burundi | 1435.211 (694.159-2673.058) | 112.086 (53.581-210.326) | 2548.409 (1179.506-4935.578) | 85.223 (39.094-165.763) | -0.6 (-0.77 to -0.42) |
| Republic of Cabo Verde | 152.73 (75.57-286.86) | 191.264 (94.077-360.939) | 221.465 (111.69-407.336) | 145.498 (73.359-267.691) | -0.81 (-0.89 to -0.73) |
| Republic of Cameroon | 4781.378 (2403.714-8702.154) | 201.755 (99.564-370.231) | 11973.855 (6115.138-21924.484) | 158.843 (80.413-293.105) | -0.41 (-0.53 to -0.3) |
| Republic of Chad | 3807.299 (1806.877-7071.173) | 286.89 (133.819-536.961) | 9386.906 (4423.182-18028.074) | 244.122 (113.436-472.001) | -0.25 (-0.35 to -0.15) |
| Republic of Chile | 850.249 (464.652-1532.338) | 23.459 (12.775-42.351) | 997.957 (531.617-1815.403) | 20.365 (10.863-36.997) | -0.21 (-0.33 to -0.09) |
| Republic of Colombia | 16119.776 (7037.31-32048.293) | 180.704 (78.028-362.598) | 12140.637 (4942.221-24494.774) | 91.737 (37.429-184.956) | -1.97 (-2.28 to -1.66) |
| Republic of Costa Rica | 303.622 (130.565-620.217) | 38.073 (16.299-78.453) | 314.239 (132.575-634.707) | 23.309 (9.856-47.016) | -1.34 (-1.56 to -1.11) |
| Republic of Croatia | 562.92 (288.923-1040.218) | 46.754 (24.121-86.043) | 288.906 (146.44-540.562) | 29.42 (15.008-54.885) | -1.46 (-1.73 to -1.19) |
| Republic of Cuba | 620.85 (271.379-1257.688) | 19.996 (8.695-40.618) | 194.162 (76.416-403.348) | 7.137 (2.843-14.76) | -3.1 (-3.47 to -2.72) |
| Republic of Cyprus | 101.332 (49.801-190.32) | 51.182 (25.229-96.002) | 97.649 (48.661-183.406) | 22.579 (11.32-42.258) | -2.75 (-3.02 to -2.47) |
| Republic of C么te d'Ivoire | 6482.344 (3290.483-11727.353) | 237.655 (119.175-435.14) | 11285.763 (5651.5-20838.508) | 173.971 (86.292-322.199) | -0.73 (-0.86 to -0.6) |
| Republic of Djibouti | 141.745 (68.331-270.835) | 142.881 (68.104-276.217) | 413.829 (188.486-797.3) | 127.989 (58.313-246.494) | -0.46 (-0.51 to -0.4) |
| Republic of Ecuador | 886.136 (503.807-1542.485) | 35.777 (20.235-62.871) | 1321.186 (744.73-2331.083) | 28.094 (15.826-49.607) | -0.5 (-0.66 to -0.34) |
| Republic of El Salvador | 531.392 (223.697-1082.707) | 39.6 (16.098-81.93) | 394.952 (159.263-818.554) | 22.182 (8.94-45.995) | -1.61 (-1.84 to -1.39) |
| Republic of Equatorial Guinea | 114.927 (51.677-222.119) | 115.524 (50.94-224.78) | 283.56 (141.042-530.585) | 77.53 (38.065-146.017) | -1.3 (-1.37 to -1.24) |
| Republic of Estonia | 238.514 (113.879-455.39) | 62.699 (30.094-119.096) | 103.023 (45.796-205.806) | 33.492 (14.997-66.641) | -1.86 (-2.08 to -1.64) |
| Republic of Fiji | 314.898 (153.079-590.576) | 159.933 (77.271-301.115) | 155.267 (73.901-304.482) | 67.325 (32.044-131.921) | -2.92 (-3.24 to -2.6) |
| Republic of Finland | 407.643 (188.31-794.341) | 32.662 (15.285-63.259) | 349.684 (162.759-677.117) | 31.159 (14.679-59.912) | -0.11 (-0.15 to -0.08) |
| Republic of Ghana | 10927.331 (5463.416-20042.092) | 306.038 (150.654-565.759) | 18877.287 (9169.297-36048.416) | 213.485 (102.887-409.305) | -0.77 (-0.96 to -0.58) |
| Republic of Guatemala | 1417.506 (622.985-2819.235) | 75.363 (32.493-151.86) | 2359.791 (998.997-4864.848) | 54.109 (22.773-112.192) | -0.85 (-0.97 to -0.72) |
| Republic of Guinea | 4056.913 (2047.639-7592.349) | 296.302 (148.878-557.168) | 8889.151 (4437.702-16419.316) | 267.409 (132.055-496.644) | -0.41 (-0.53 to -0.28) |
| Republic of Guinea-Bissau | 839.625 (416.545-1556.756) | 358.996 (175.01-671.665) | 1556.812 (752.544-2933.127) | 293.799 (140.8-557.155) | -0.56 (-0.61 to -0.51) |
| Republic of Guyana | 84.471 (40.371-160.102) | 40.618 (19.152-78.112) | 47.377 (22.313-93.547) | 23.717 (11.134-46.868) | -0.82 (-1.08 to -0.57) |
| Republic of Haiti | 603.154 (291.651-1141.212) | 38.81 (18.511-73.872) | 1197.136 (581.39-2289.024) | 33.585 (16.271-64.35) | -0.28 (-0.35 to -0.2) |
| Republic of Honduras | 631.456 (279.198-1237.883) | 58.97 (25.318-117.136) | 933.333 (389.044-1903.87) | 34.456 (14.26-70.525) | -1.3 (-1.6 to -1) |
| Republic of Iceland | 16.033 (7.257-31.596) | 24.528 (11.108-48.378) | 17.434 (8.185-34.055) | 22.1 (10.477-42.973) | -0.23 (-0.31 to -0.15) |
| Republic of India | 196417.146 (100421.79-360611.181) | 96.772 (49.043-178.63) | 301501.217 (161620.012-548785.746) | 79.449 (42.566-144.733) | -0.74 (-0.77 to -0.71) |
| Republic of Indonesia | 79258.149 (42377.083-143460.514) | 164.977 (87.311-300.211) | 81461.421 (43832.555-149736.522) | 106.351 (57.378-195.205) | -1.43 (-1.65 to -1.21) |
| Republic of Iraq | 1750.288 (1009.535-3006.434) | 44.49 (25.482-77.454) | 3177.169 (1825.606-5607.388) | 31.488 (18.042-55.69) | -1.03 (-1.18 to -0.87) |
| Republic of Italy | 12903.449 (6649.521-23647.099) | 90.523 (46.8-165.587) | 3746.062 (1787.761-7304.545) | 24.89 (11.955-48.198) | -4.21 (-4.57 to -3.86) |
| Republic of Kazakhstan | 2136.296 (1156.405-3837.176) | 51.439 (27.798-92.435) | 1683.162 (886.263-3079.398) | 32.685 (17.237-59.723) | -1.11 (-1.31 to -0.91) |
| Republic of Kenya | 10522.281 (5541.674-19221.227) | 203.615 (105.423-375.373) | 14128.442 (7130.842-26708.573) | 115.072 (57.629-218.798) | -1.34 (-1.61 to -1.06) |
| Republic of Kiribati | 63.074 (32.165-114.608) | 335.267 (169.071-613.13) | 64.679 (32.153-120.613) | 206.835 (102.522-386.32) | -1.42 (-1.58 to -1.26) |
| Republic of Korea | 18945.374 (9432.811-35086.067) | 147.606 (73.527-273.266) | 6387.397 (3334.594-11889.976) | 46.06 (24.026-85.659) | -4 (-4.21 to -3.78) |
| Republic of Latvia | 254.549 (108.204-512.307) | 39.45 (16.883-79.125) | 80.546 (33.598-168.191) | 18.684 (7.836-38.714) | -1.93 (-2.2 to -1.66) |
| Republic of Liberia | 1734.141 (812.228-3200.006) | 311.975 (144.834-578.446) | 4163.662 (2146.098-7669.188) | 300.498 (153.522-556.39) | -0.32 (-0.41 to -0.23) |
| Republic of Lithuania | 476.192 (206.559-933.8) | 51.675 (22.528-101.108) | 158.067 (69.698-314.911) | 25.454 (11.287-50.703) | -1.95 (-2.22 to -1.68) |
| Republic of Madagascar | 4962.194 (2206.081-9743.847) | 180.846 (79.046-359.389) | 10280.749 (4470.817-20328.255) | 146.729 (63.492-291.681) | -0.4 (-0.52 to -0.28) |
| Republic of Malawi | 3443.699 (1572.458-6644.363) | 150.12 (66.836-292.563) | 5231.058 (2379.611-10000.014) | 111.282 (50.099-214.104) | -0.96 (-1.09 to -0.83) |
| Republic of Maldives | 48.161 (21.282-93.716) | 99.613 (42.824-196.504) | 56.722 (24.06-114.98) | 44.08 (18.691-89.178) | -2.21 (-2.53 to -1.88) |
| Republic of Mali | 3698.601 (1734.473-7099.822) | 191.989 (88.567-372.381) | 8622.661 (4119.866-16445.887) | 164.26 (77.341-316.402) | -0.53 (-0.68 to -0.38) |
| Republic of Malta | 21.874 (9.679-43.649) | 23.416 (10.443-46.606) | 13.939 (5.969-28.514) | 13.374 (5.796-27.123) | -1.4 (-1.65 to -1.15) |
| Republic of Mauritius | 259.933 (111.179-522.002) | 85.507 (36.594-172.188) | 169.164 (69.211-343.668) | 51.581 (21.235-104.563) | -1.35 (-1.65 to -1.06) |
| Republic of Moldova | 3322.71 (1626.849-6219.074) | 293.01 (143.687-547.95) | 1380.544 (656.637-2690.808) | 131.574 (63.225-254.987) | -2.01 (-2.3 to -1.72) |
| Republic of Mozambique | 8763.995 (4210.384-16280.339) | 278.92 (132.411-523.044) | 14534.848 (7235.644-27009.073) | 207.055 (101.85-386.388) | -0.67 (-0.8 to -0.55) |
| Republic of Namibia | 684.241 (339.837-1281.49) | 199.368 (97.447-378.43) | 1076.985 (554.496-2008.283) | 161.752 (82.728-303.537) | -0.55 (-0.6 to -0.5) |
| Republic of Nauru | 6.325 (3.234-11.56) | 259.291 (131.582-476.705) | 2.8 (1.357-5.268) | 105.286 (50.755-198.537) | -3.04 (-3.3 to -2.77) |
| Republic of Nicaragua | 150.078 (72.264-288.046) | 16.427 (7.837-31.717) | 185.442 (83.588-358.799) | 10.251 (4.61-19.857) | -1.34 (-1.62 to -1.06) |
| Republic of Niue | 0.935 (0.468-1.766) | 189.721 (94.513-359.258) | 0.194 (0.093-0.371) | 44.96 (21.605-85.814) | -3.82 (-4.31 to -3.33) |
| Republic of Palau | 8.675 (4.478-15.658) | 210.866 (108.169-381.852) | 3.993 (1.936-7.616) | 90.323 (44.018-172.862) | -2.9 (-3.23 to -2.56) |
| Republic of Panama | 180.652 (74.776-368.969) | 28.927 (11.804-59.564) | 185.26 (79.477-376.417) | 17.46 (7.498-35.468) | -0.95 (-1.16 to -0.73) |
| Republic of Paraguay | 450.901 (208.558-862.033) | 47.518 (21.773-91.633) | 685.272 (322.288-1329.747) | 35.966 (16.9-69.801) | -0.75 (-0.91 to -0.59) |
| Republic of Peru | 4328.376 (2236.791-7836.894) | 79.853 (40.846-145.874) | 6662.521 (3466.252-12216.535) | 68.115 (35.476-124.769) | -0.5 (-0.59 to -0.41) |
| Republic of Poland | 4893.726 (2796.439-8584.468) | 51.679 (29.625-90.134) | 1404.005 (713.879-2630.271) | 13.509 (6.899-25.245) | -4.16 (-4.42 to -3.91) |
| Republic of Rwanda | 1975.744 (823.46-4041.704) | 119.122 (48.544-247.513) | 2949.009 (1112.265-6149.482) | 86.024 (31.955-180.369) | -0.65 (-0.83 to -0.46) |
| Republic of San Marino | 2.065 (1.011-3.835) | 33.069 (16.206-61.397) | 1.403 (0.683-2.648) | 17.425 (8.528-32.963) | -1.77 (-2.04 to -1.49) |
| Republic of Senegal | 4510.378 (2184.696-8453.767) | 261.996 (124.407-495.21) | 8210.389 (3885.462-15790.63) | 217.929 (102.407-421.4) | -0.65 (-0.76 to -0.54) |
| Republic of Serbia | 903.762 (430.392-1708.182) | 38.771 (18.522-73.146) | 594.986 (280.362-1126.367) | 28.62 (13.568-54.094) | -0.72 (-0.85 to -0.6) |
| Republic of Seychelles | 22.341 (11.031-42.698) | 122.201 (59.712-234.989) | 15.74 (7.762-30.037) | 60.259 (29.785-114.974) | -1.98 (-2.32 to -1.63) |
| Republic of Sierra Leone | 2331.852 (1128.424-4311.54) | 231.321 (110.702-430.78) | 4584.639 (2144.455-8628.152) | 201.898 (93.746-382.283) | -0.41 (-0.51 to -0.3) |
| Republic of Singapore | 1322.578 (708.95-2376.546) | 140.025 (75.174-251.394) | 1061.527 (566.404-1960.649) | 56.845 (30.556-104.58) | -2.99 (-3.28 to -2.69) |
| Republic of Slovenia | 271.531 (128.128-513.274) | 54.644 (25.886-102.91) | 151.59 (70.258-299.361) | 32.895 (15.425-64.435) | -1.41 (-1.66 to -1.15) |
| Republic of South Africa | 7778.57 (4093.39-13952.84) | 79.939 (41.611-144.136) | 5173.838 (2647.697-9700.23) | 32.401 (16.62-60.614) | -3.02 (-3.32 to -2.73) |
| Republic of South Sudan | 2239.692 (1026.326-4322.072) | 171.691 (76.996-333.27) | 3806.478 (1847.324-7225.549) | 165.56 (78.92-315.465) | -0.09 (-0.12 to -0.05) |
| Republic of Sudan | 8626.969 (4040.038-16420.135) | 184.021 (84.669-352.52) | 15574.201 (7860.689-28953.595) | 139.973 (70.092-261.186) | -0.95 (-1.03 to -0.87) |
| Republic of Suriname | 27.79 (12.397-55.354) | 28.197 (12.404-56.562) | 31.422 (13.813-61.809) | 21.538 (9.478-42.351) | -0.71 (-0.88 to -0.53) |
| Republic of Tajikistan | 1254.281 (625.587-2297.052) | 103.175 (50.91-191.402) | 2075.503 (1038.06-3899.769) | 80.758 (40.278-152.131) | -0.63 (-0.74 to -0.52) |
| Republic of Trinidad and Tobago | 84.15 (37.342-170.886) | 26.878 (11.839-54.82) | 69.763 (32.017-138.54) | 20.36 (9.394-40.271) | -0.74 (-0.84 to -0.65) |
| Republic of Tunisia | 3302.066 (1636.901-6033.36) | 159.753 (78.162-294.483) | 2485.323 (1274.044-4639.446) | 72.73 (37.372-135.58) | -2.3 (-2.54 to -2.06) |
| Republic of Turkey | 15664.269 (8390.03-27658.792) | 110.401 (58.822-196.324) | 15052.998 (8251.302-26798.879) | 67.342 (36.991-119.738) | -1.46 (-1.61 to -1.32) |
| Republic of Uganda | 6536.316 (3056.463-12214.433) | 168.173 (76.742-318.814) | 14204.036 (7034.798-26458.603) | 143.534 (70.543-269.081) | -0.43 (-0.52 to -0.35) |
| Republic of Uzbekistan | 9868.314 (4804.803-18349.332) | 198.432 (94.798-372.255) | 13483.968 (6520.632-25595.235) | 146.597 (71.009-278.32) | -0.94 (-1.11 to -0.77) |
| Republic of Vanuatu | 105.403 (51.241-197.577) | 299.313 (143.557-565.473) | 128.804 (63.619-243.978) | 171.357 (84.137-325.973) | -1.63 (-1.78 to -1.49) |
| Republic of Yemen | 6997.703 (3343.081-13108.795) | 253.098 (118.789-478.111) | 15153.946 (7644.408-27916.826) | 187.029 (93.895-346.557) | -0.93 (-0.99 to -0.87) |
| Republic of Zambia | 2710.054 (1333.225-4984.397) | 147.443 (70.933-275.629) | 5595.698 (2916.181-10297.164) | 118.161 (61.104-219.042) | -0.62 (-0.72 to -0.51) |
| Republic of Zimbabwe | 13510.07 (6659.274-24945.69) | 562.427 (271.183-1054.369) | 16753.265 (8099.286-31616.578) | 430.857 (206.656-817.674) | -0.7 (-0.88 to -0.51) |
| Republic of the Congo | 1313.581 (588.182-2545.506) | 230.307 (100.755-451.202) | 2629.513 (1199.155-5148.314) | 183.285 (82.949-360.531) | -0.54 (-0.6 to -0.48) |
| Republic of the Gambia | 517.469 (267.531-946.455) | 227.705 (116.202-419.198) | 556.726 (279.632-1030.544) | 106.442 (52.853-198.19) | -2.44 (-2.66 to -2.21) |
| Republic of the Marshall Islands | 22.283 (11.267-40.326) | 226.842 (112.173-416.018) | 18.574 (8.946-34.951) | 127.888 (61.476-240.873) | -1.71 (-1.95 to -1.47) |
| Republic of the Niger | 5844.473 (2674.532-11345.933) | 334.847 (150.504-656.828) | 15927.331 (7779.686-29634.92) | 300.362 (142.645-568.845) | -0.08 (-0.16 to 0.01) |
| Republic of the Philippines | 62668.521 (32751.765-114654.018) | 402.107 (207.444-740.785) | 68272.797 (34717.802-127183.765) | 237.889 (120.687-444.083) | -1.53 (-1.69 to -1.36) |
| Republic of the Union of Myanmar | 4945.317 (2228.697-9746.798) | 46.276 (20.779-91.533) | 5330.578 (2434.515-10673.281) | 35.407 (16.171-70.918) | -0.7 (-0.81 to -0.59) |
| Romania | 6936.784 (3512.32-12920.728) | 123.773 (62.657-230.453) | 2527.169 (1235.603-4820.435) | 52.807 (26.022-100.45) | -2.43 (-2.71 to -2.15) |
| Russian Federation | 27274.137 (13159.199-51007.787) | 73.132 (35.513-136.379) | 16386.578 (7725.432-31671.146) | 45.339 (21.542-86.859) | -1.12 (-1.55 to -0.68) |
| Saint Kitts and Nevis | 2.929 (1.239-5.942) | 28.226 (11.771-57.82) | 2.745 (1.206-5.623) | 16.807 (7.411-34.397) | -1.63 (-1.95 to -1.31) |
| Saint Lucia | 11.378 (5.425-21.749) | 32.832 (15.437-63.219) | 10.908 (5.124-21.454) | 23.329 (11.021-45.839) | -0.79 (-0.95 to -0.64) |
| Saint Vincent and the Grenadines | 6.252 (2.639-12.862) | 22.935 (9.534-47.684) | 4.698 (2.013-9.513) | 16.846 (7.24-34.001) | -0.76 (-0.93 to -0.58) |
| Slovak Republic | 756.375 (375.844-1439.515) | 57.113 (28.469-108.379) | 467.635 (213.434-914.733) | 32.984 (15.258-63.987) | -1.51 (-1.8 to -1.23) |
| Socialist Republic of Viet Nam | 43623.547 (21727.787-81371.723) | 249.599 (122.875-468.733) | 50931.719 (24981.257-95701.446) | 192.146 (94.847-359.733) | -0.7 (-0.85 to -0.54) |
| Solomon Islands | 267.261 (134.816-485.547) | 356.2 (175.329-655.207) | 272.68 (125.24-531.312) | 167.928 (76.27-328.666) | -2.35 (-2.62 to -2.07) |
| State of Eritrea | 1655.913 (765.128-3180.277) | 208.302 (94.974-405.259) | 2491.118 (1158.546-4813.632) | 154.128 (71.134-298.209) | -0.73 (-0.87 to -0.58) |
| State of Israel | 249.334 (131.629-459.045) | 20.465 (10.785-37.696) | 237.058 (121.021-439.421) | 10.363 (5.291-19.216) | -2.17 (-2.51 to -1.84) |
| State of Kuwait | 156.572 (83.801-281.005) | 37.366 (19.966-67.31) | 272.271 (143.221-499.354) | 14.835 (7.84-27.17) | -2.9 (-3.22 to -2.57) |
| State of Libya | 793.071 (430.433-1412.056) | 89.007 (47.684-160.555) | 1209.81 (650.873-2208.477) | 58.629 (31.607-106.84) | -1.09 (-1.22 to -0.96) |
| State of Qatar | 104.202 (60.077-181.529) | 130.947 (75.648-228.31) | 399.431 (225.204-704.727) | 63.335 (35.98-111.358) | -2.36 (-2.74 to -1.98) |
| Sultanate of Oman | 488.898 (257.329-867.501) | 144.163 (74.949-257.738) | 485.903 (244.606-906.125) | 45.709 (23.047-84.993) | -3.34 (-3.82 to -2.86) |
| Swiss Confederation | 514.202 (250.317-991.563) | 29.238 (14.374-56.066) | 527.005 (254.647-1012.6) | 27.128 (13.318-51.564) | -0.31 (-0.36 to -0.26) |
| Syrian Arab Republic | 4276.847 (2402.079-7548.136) | 158.686 (88.298-283.76) | 3699.309 (2058.174-6623.078) | 100.486 (55.617-180.35) | -1.31 (-1.55 to -1.08) |
| Taiwan (Province of China) | 23876.395 (12596.888-43301.292) | 425.051 (223.575-770.804) | 7707.496 (4078.677-13881.571) | 107.439 (57.298-192.909) | -3.72 (-4.16 to -3.27) |
| Togolese Republic | 2545.007 (1218.94-4824.437) | 295.997 (139.483-568.472) | 5398.57 (2618.913-10026.908) | 249.407 (120.095-464.798) | -0.59 (-0.68 to -0.5) |
| Tokelau | 0.967 (0.503-1.778) | 275.123 (142.316-507.628) | 0.432 (0.224-0.81) | 135.72 (70.488-254.317) | -2.01 (-2.33 to -1.7) |
| Turkmenistan | 845.235 (432.488-1554.708) | 94.256 (47.502-174.939) | 912.896 (458.247-1730.88) | 72.311 (36.244-137.143) | -0.83 (-0.93 to -0.73) |
| Tuvalu | 6.906 (3.416-12.597) | 282.446 (139.336-515.682) | 4.955 (2.455-9.555) | 177.238 (87.458-342.698) | -1.22 (-1.41 to -1.03) |
| Ukraine | 4660.852 (2156.998-9040.954) | 36.854 (17.166-71.251) | 3133.691 (1414.114-6114.69) | 29.592 (13.608-57.331) | -0.19 (-0.53 to 0.14) |
| Union of the Comoros | 166.614 (75.939-317.067) | 156.288 (69.593-300.484) | 227.82 (100.036-449.906) | 117.709 (51.252-232.852) | -0.58 (-0.71 to -0.46) |
| United Arab Emirates | 159.857 (90.413-280.437) | 46.241 (26.155-81.361) | 534.95 (290.763-955.023) | 21.475 (11.794-38.217) | -2.33 (-2.62 to -2.03) |
| United Kingdom of Great Britain and Northern Ireland | 4504.135 (2188.338-8456.69) | 31.819 (15.563-59.57) | 4935.16 (2531.686-9150.107) | 32.428 (16.778-59.696) | 0.15 (0.12 to 0.18) |
| United Mexican States | 5775.997 (2915.828-10603.826) | 26.149 (13.063-48.288) | 4537.617 (2196.301-8776.166) | 12.833 (6.216-24.816) | -2.49 (-2.76 to -2.23) |
| United Republic of Tanzania | 9611.916 (4580.605-18080.923) | 157.692 (72.962-300.276) | 17697.328 (8406.929-33124.505) | 124.238 (58.565-234.028) | -0.61 (-0.77 to -0.46) |
| United States Virgin Islands | 6.452 (2.986-12.682) | 22.706 (10.546-44.535) | 1.729 (0.787-3.402) | 9.187 (4.198-17.989) | -2.93 (-3.14 to -2.72) |
| United States of America | 11613.253 (5954.527-21596.438) | 17.23 (8.895-31.922) | 6590.47 (3262.377-12309.228) | 8.124 (4.033-15.162) | -1.92 (-2.33 to -1.5) |
| (SDI: Socio-Demographic Index, ASR: Age-standardized rate, EAPC: Estimated Annual Percentage Change, UI: uncertainty intervals, CI: confidence intervals, AHB: Acute hepatitis B, ASPR: Age-standardized rate of Prevalence) | | | | | |

| **Table S2. Incidence cases and ASIR of AHB in 1990 and 2021 and its trends (National level).** | | | | | |
| --- | --- | --- | --- | --- | --- |
| **location** | **Number (95% UI) 1990** | **ASR (95% UI) 1990** | **Number (95% UI) 2021** | **ASR (95% UI) 2021** | **EAPC (95%CI) 1990–2021** |
| American Samoa | 218.182 (113.597-395.391) | 1806.632 (933.719-3295.433) | 118.267 (60.419-224.288) | 1020.118 (520.732-1937.797) | -1.77 (-2.04 to -1.51) |
| Antigua and Barbuda | 35.989 (17.237-70.776) | 218.242 (103.742-430.622) | 34.158 (15.148-66.637) | 134.522 (59.926-262.129) | -1.48 (-1.76 to -1.2) |
| Arab Republic of Egypt | 196494.82 (91676.527-369144.996) | 1504.285 (692.086-2847.33) | 177742.05 (82962.586-339128.281) | 708.58 (329.966-1351.848) | -2.09 (-2.4 to -1.78) |
| Argentine Republic | 10989.56 (5918.52-19662.981) | 137.27 (73.821-245.89) | 13445.486 (7160.613-24344.892) | 110.393 (58.846-199.807) | -0.5 (-0.66 to -0.34) |
| Australia | 28349.463 (14115.492-51885.231) | 634.349 (316.402-1158.032) | 27127.215 (13211.554-51366.991) | 416.898 (204.146-787.056) | -1.28 (-1.53 to -1.03) |
| Barbados | 111.15 (46.253-225.784) | 160.287 (66.647-326.241) | 78.961 (32.618-164.897) | 108.777 (45.165-226.225) | -0.89 (-1.06 to -0.72) |
| Belize | 127.098 (62.082-239.51) | 297.084 (141.432-565.141) | 228.645 (104.147-449.623) | 195.93 (88.841-386.08) | -1.03 (-1.2 to -0.85) |
| Bermuda | 28.22 (12.766-56.946) | 164.293 (75.015-329.6) | 16.667 (7.387-32.621) | 120.639 (54.41-235.401) | -0.84 (-0.94 to -0.74) |
| Bolivarian Republic of Venezuela | 9878.81 (3998.601-20977.06) | 197.601 (79.184-421.597) | 9206.535 (3593.751-19488.375) | 133.337 (52.281-280.912) | -0.95 (-1.1 to -0.8) |
| Bosnia and Herzegovina | 5768.301 (3017.098-10493.491) | 492.965 (257.778-897.077) | 2491.959 (1218.238-4749.593) | 328.899 (161.567-624.89) | -0.94 (-1.05 to -0.82) |
| Brunei Darussalam | 888.276 (432.778-1679.663) | 1291.818 (622.956-2460.071) | 460.15 (210.531-882.779) | 350.003 (160.551-670.683) | -4.4 (-4.76 to -4.04) |
| Burkina Faso | 59736.474 (30027.128-109009.657) | 2879.996 (1431.507-5295) | 119355.607 (61635.049-219710.999) | 2287.924 (1173.589-4227.855) | -0.56 (-0.69 to -0.43) |
| Canada | 35036.677 (16883.977-65726.228) | 473.978 (230.44-883.629) | 34253.03 (17201.238-63834.673) | 410.391 (207.999-759.285) | -0.53 (-0.59 to -0.48) |
| Central African Republic | 14688.824 (6292.945-28335.561) | 2264.143 (958.478-4410.519) | 28670.386 (12387.633-55860.495) | 2061.099 (860.317-4050.005) | -0.3 (-0.35 to -0.26) |
| Commonwealth of Dominica | 44.787 (21.059-87.047) | 258.433 (119.306-507.589) | 33.233 (14.915-65.326) | 202.597 (90.881-398.126) | -0.48 (-0.56 to -0.39) |
| Commonwealth of the Bahamas | 169.793 (72.178-333.044) | 229.397 (96.514-452.094) | 165.985 (71.746-334.538) | 153.588 (66.749-309.069) | -0.81 (-1 to -0.61) |
| Cook Islands | 49.261 (23.293-93.417) | 1066.046 (498.529-2035.728) | 18.05 (8.257-34.854) | 411.968 (188.962-797.608) | -2.99 (-3.33 to -2.65) |
| Czech Republic | 8556.406 (3854.347-16467.968) | 337.99 (154.018-645.691) | 4619.822 (2099.933-9208.649) | 185.596 (85.316-366.952) | -1.11 (-1.34 to -0.88) |
| Democratic People's Republic of Korea | 178186.401 (82238.899-346659.989) | 3134.212 (1441.192-6127.463) | 165699.692 (75048.675-329527.488) | 2460.558 (1123.665-4885.647) | -0.74 (-0.88 to -0.6) |
| Democratic Republic of Sao Tome and Principe | 539.419 (270.614-997.099) | 2111.551 (1041.418-3931.4) | 921.467 (461.944-1721.931) | 1699.219 (846.276-3187.646) | -0.63 (-0.82 to -0.44) |
| Democratic Republic of Timor-Leste | 2450.272 (1124.928-4773.342) | 1297.332 (590.814-2539.082) | 3897.435 (1847.393-7481.278) | 1120.266 (522.933-2179.953) | -0.39 (-0.47 to -0.32) |
| Democratic Republic of the Congo | 237069.896 (112249.876-452113.545) | 2768.115 (1291.35-5351.442) | 563564.52 (279462.272-1057162.421) | 2654.19 (1298.862-5016.478) | -0.23 (-0.32 to -0.15) |
| Democratic Socialist Republic of Sri Lanka | 30003.038 (15831.119-54699.763) | 653.652 (343.85-1195.597) | 29473.802 (15912.544-53490.539) | 518.319 (280.202-941.257) | -0.51 (-0.65 to -0.37) |
| Dominican Republic | 7026.661 (3435.816-12844.089) | 370.915 (180.215-684.315) | 7095.777 (3513.56-13426.253) | 245.884 (121.62-465.435) | -1.14 (-1.33 to -0.96) |
| Eastern Republic of Uruguay | 874.677 (448.881-1627.656) | 116.567 (59.824-216.98) | 710.441 (362.607-1319.313) | 82.563 (42.23-153.305) | -0.95 (-1.22 to -0.68) |
| Federal Democratic Republic of Ethiopia | 187741.738 (96239.144-345896.921) | 1649.135 (833.174-3063.234) | 323904.56 (166686.967-596541.595) | 1160.721 (589.462-2153.724) | -1.17 (-1.26 to -1.08) |
| Federal Democratic Republic of Nepal | 25014.979 (14332.316-43385.956) | 559.096 (318.519-976.557) | 42651.343 (24465.836-74691.48) | 477.323 (273.054-837.373) | -0.36 (-0.42 to -0.29) |
| Federal Republic of Germany | 27076.885 (13192.752-50835.601) | 138.742 (68.162-259.444) | 15708.708 (7816.644-29397.467) | 83.011 (41.532-154.804) | -1.33 (-1.61 to -1.04) |
| Federal Republic of Nigeria | 647208.399 (340294.197-1169888.095) | 3184.722 (1641.519-5810.535) | 1467783.838 (778406.938-2680411.187) | 2607.098 (1364.862-4802.796) | -0.66 (-0.72 to -0.59) |
| Federal Republic of Somalia | 49342.934 (23580.537-94955.386) | 2889.36 (1360.003-5606.727) | 127156.817 (60930.726-241131.664) | 2594.666 (1225.178-4975.289) | -0.37 (-0.4 to -0.34) |
| Federated States of Micronesia | 470.946 (243.932-849.915) | 2025.15 (1032.028-3699.353) | 235.733 (118.99-440.129) | 954.522 (478.99-1789.589) | -2.5 (-2.77 to -2.22) |
| Federative Republic of Brazil | 224547.562 (116680.024-401838.61) | 573.507 (295.936-1030.649) | 148461.097 (68880.954-293520.267) | 240.552 (111.979-474.502) | -2.73 (-3.04 to -2.43) |
| French Republic | 27595.54 (13989.593-51441.82) | 190.481 (96.765-354.661) | 21011.877 (10498.366-39164.847) | 146.302 (73.605-271.712) | -0.65 (-0.74 to -0.55) |
| Gabonese Republic | 3637.348 (1692.845-6974.907) | 1613.585 (742.158-3139.262) | 6429.982 (3164.549-12095.756) | 1322.722 (645.923-2506.611) | -0.51 (-0.6 to -0.43) |
| Georgia | 7336.933 (3653.482-13673.763) | 529.988 (264.559-987.596) | 3368.584 (1703.69-6277.594) | 405.459 (206.848-752.713) | -0.71 (-0.85 to -0.56) |
| Grand Duchy of Luxembourg | 283.057 (135.465-535.317) | 289.864 (139.843-546.134) | 303.671 (139.741-583.561) | 173.454 (80.352-332.756) | -1.2 (-1.47 to -0.93) |
| Greenland | 86.032 (38.068-163.706) | 566.586 (251.898-1080.126) | 64.659 (27.817-130.091) | 504.394 (217.22-1012.203) | -0.21 (-0.25 to -0.17) |
| Grenada | 48.884 (21.905-97.484) | 244.659 (108.161-492.713) | 35.477 (14.051-74.225) | 138.613 (54.95-290.177) | -1.29 (-1.53 to -1.05) |
| Guam | 551.578 (300.646-986.961) | 1570.205 (856.547-2813.631) | 302.618 (159.827-556.431) | 823.55 (435.929-1515.963) | -2.21 (-2.48 to -1.93) |
| Hashemite Kingdom of Jordan | 9427.651 (4533.93-17749.856) | 1137.308 (536.544-2161.874) | 16486.443 (8359.126-31156.94) | 574.446 (291.033-1086.927) | -1.93 (-2.24 to -1.63) |
| Hellenic Republic | 18347.803 (9932.822-33175.071) | 727.521 (394.821-1313.535) | 10602.265 (5564.499-19569.762) | 436.41 (230.748-802.703) | -1.45 (-1.66 to -1.24) |
| Hungary | 9198.059 (4775.191-16488.434) | 367.399 (192.263-655.406) | 6848.225 (3523.158-12673.198) | 330.077 (172.855-605.991) | -0.23 (-0.27 to -0.19) |
| Independent State of Papua New Guinea | 23957.608 (10774.01-46290.598) | 2442.965 (1074.033-4757.04) | 45176.303 (20878.824-86624.747) | 1750.655 (804.446-3369.13) | -1.18 (-1.35 to -1.02) |
| Independent State of Samoa | 755.284 (388.281-1401.243) | 2067.737 (1048.104-3887.076) | 662.217 (339.198-1236.16) | 1402.925 (712.882-2633.894) | -1.24 (-1.37 to -1.1) |
| Ireland | 2021.681 (931.693-3913.236) | 229.004 (105.198-443.996) | 2399.546 (1146.642-4624.556) | 210.334 (101.78-402.511) | -0.34 (-0.37 to -0.3) |
| Islamic Republic of Afghanistan | 19716.821 (11261.511-34166.365) | 942.157 (531.225-1652.045) | 58436.444 (34159.527-100494.764) | 856.801 (496.256-1487.032) | -0.19 (-0.25 to -0.12) |
| Islamic Republic of Iran | 129390.022 (72166.747-226179.057) | 1033.304 (569.7-1826.67) | 112048.139 (62207.832-203114.698) | 411.233 (228.89-741.863) | -3.16 (-3.39 to -2.93) |
| Islamic Republic of Mauritania | 17397.327 (8934.096-31944.599) | 3706.368 (1883.134-6871.526) | 30985.15 (16657.535-56105.643) | 2988.102 (1585.105-5453.423) | -0.58 (-0.67 to -0.49) |
| Islamic Republic of Pakistan | 228273.123 (116766.867-420687.861) | 969.217 (487.743-1800.729) | 409402.538 (207754.543-764895.309) | 687.322 (346.976-1288.862) | -0.59 (-0.73 to -0.44) |
| Jamaica | 1826.191 (824.543-3629.108) | 296.257 (131.547-594.071) | 1669.577 (695.33-3462.121) | 210.324 (87.368-436.35) | -0.95 (-1.11 to -0.8) |
| Japan | 259432.883 (144446.945-460581.568) | 811.148 (453.15-1433.164) | 156081.257 (86253.64-280160.452) | 631.674 (351.937-1125.982) | -0.92 (-0.97 to -0.87) |
| Kingdom of Bahrain | 1075.613 (598.514-1892.391) | 927.324 (517.35-1636.414) | 1548.603 (865.97-2726.823) | 453.228 (253.534-797.571) | -1.92 (-2.26 to -1.58) |
| Kingdom of Belgium | 4601.785 (2215.697-8666.719) | 188.768 (91.394-354.43) | 3316.949 (1547.239-6442.454) | 127.213 (59.517-246.31) | -0.87 (-1.06 to -0.69) |
| Kingdom of Bhutan | 1315.16 (637.781-2474.162) | 927.511 (439.892-1765.072) | 1053.822 (522.561-1961.58) | 506.162 (250.859-941.845) | -1.79 (-2.02 to -1.57) |
| Kingdom of Cambodia | 48581.537 (21437.342-96761.313) | 1903.987 (830.997-3831.393) | 56980.257 (25784.618-112562.349) | 1240.79 (558.597-2454.199) | -0.79 (-0.99 to -0.6) |
| Kingdom of Denmark | 3071.51 (1461.259-5898.345) | 236.969 (113.56-452.523) | 3043.122 (1490.219-5762.45) | 242.995 (120.224-458.469) | 0.09 (0.03 to 0.14) |
| Kingdom of Eswatini | 3645.451 (1726.927-6808.156) | 1858.978 (862.581-3514.437) | 2752.811 (1355.934-5168.531) | 933.542 (456.028-1755.36) | -1.67 (-1.96 to -1.38) |
| Kingdom of Lesotho | 7391.591 (3580.825-14079.177) | 1950.897 (934.914-3733.599) | 6722.293 (3108.568-12894.688) | 1350.188 (618.461-2599.978) | -1 (-1.11 to -0.89) |
| Kingdom of Morocco | 32641.113 (18293.775-56665.105) | 527.208 (293.733-922.048) | 38400.929 (21742.467-67190.205) | 389.595 (220.696-681.403) | -0.71 (-0.86 to -0.56) |
| Kingdom of Norway | 2341.368 (1160.067-4409.543) | 222.448 (110.78-418.029) | 1887.398 (890.864-3624.641) | 152.226 (72.155-291.218) | -0.99 (-1.19 to -0.79) |
| Kingdom of Saudi Arabia | 36167.576 (18139.942-66066.58) | 1116.682 (552.682-2058.432) | 48463.876 (25682.846-88702.53) | 434.286 (230.378-793.801) | -3.15 (-3.46 to -2.84) |
| Kingdom of Spain | 44629.265 (23856.105-79996.782) | 463.699 (247.867-831.924) | 25366.141 (13436.366-46534.719) | 208.143 (110.591-382.466) | -2.04 (-2.37 to -1.72) |
| Kingdom of Sweden | 3355.762 (1652.21-6329.902) | 165.195 (82.202-310.489) | 3433.881 (1721.051-6408.171) | 157.163 (79.481-291.813) | -0.22 (-0.25 to -0.2) |
| Kingdom of Thailand | 153069.998 (75016.472-283677.755) | 951.917 (465.064-1770.598) | 99893.082 (49595.757-187321.375) | 548.384 (273.088-1027.985) | -1.67 (-2.03 to -1.32) |
| Kingdom of Tonga | 456.959 (256.167-802.41) | 2093.782 (1161.166-3697.775) | 215.554 (116.485-403.475) | 924.133 (498.79-1731.675) | -3.13 (-3.55 to -2.7) |
| Kingdom of the Netherlands | 6686.183 (3480.158-12238.701) | 168.264 (87.888-306.887) | 5555.094 (2902.3-10017.599) | 150.753 (79.274-271.256) | -0.4 (-0.43 to -0.36) |
| Kyrgyz Republic | 8706.737 (4368.41-16193.74) | 818.884 (406.602-1532.114) | 9854.646 (4945.288-18658.694) | 558.904 (279.634-1060.712) | -0.87 (-1.01 to -0.72) |
| Lao People's Democratic Republic | 11698.149 (5311.046-23049.277) | 1192.003 (532.529-2380.706) | 18490.85 (8270.146-36058.127) | 923.93 (411.439-1806.034) | -0.84 (-0.95 to -0.73) |
| Lebanese Republic | 5169.707 (2864.535-9103.872) | 696.097 (385.243-1229.285) | 8737.942 (4862.13-15443.485) | 542.242 (302.948-956.904) | -0.85 (-1.03 to -0.66) |
| Malaysia | 44405.506 (24766.483-78344.792) | 998.635 (555.077-1769.228) | 36863.768 (20669.161-65489.396) | 447.134 (250.772-794.333) | -2.56 (-2.88 to -2.24) |
| Mongolia | 13131.253 (7273.635-23313.843) | 2591.29 (1426.353-4653.417) | 12453.1 (6740.769-22462.303) | 1358.5 (736.825-2447.321) | -2.04 (-2.28 to -1.79) |
| Montenegro | 416.14 (185.317-818.682) | 265.713 (118.278-522.893) | 272.463 (124.798-542.497) | 186.389 (86.176-370.304) | -0.64 (-0.77 to -0.5) |
| New Zealand | 6026.284 (2920.663-11310.487) | 665.431 (322.759-1248.462) | 3429.657 (1583.984-6517.287) | 274.318 (127.392-520.153) | -2.91 (-3.12 to -2.69) |
| North Macedonia | 2260.712 (1170.871-4152.958) | 443.623 (229.843-814.808) | 1791.343 (925.169-3300.986) | 323.353 (168.472-592.409) | -0.74 (-0.82 to -0.66) |
| Northern Mariana Islands | 317.551 (172.813-573.399) | 2278.741 (1239.799-4120.76) | 148.99 (79.587-270.431) | 1205.838 (646.102-2190.806) | -2.02 (-2.3 to -1.73) |
| Palestine | 3975.487 (2180.064-7091.582) | 914.061 (497.497-1645.665) | 5665.135 (3046.294-10175.297) | 497.413 (267.138-895.816) | -1.99 (-2.37 to -1.61) |
| People's Democratic Republic of Algeria | 56473.21 (31132.557-99315.255) | 988.637 (541.274-1755.996) | 92723.253 (51753.416-162486.22) | 798.464 (446.668-1397.113) | -0.56 (-0.65 to -0.46) |
| People's Republic of Bangladesh | 215623.305 (117821.189-384661.304) | 892.725 (484.469-1609.596) | 315019.779 (170239.216-563694.638) | 687.421 (371.011-1231.779) | -0.52 (-0.64 to -0.41) |
| People's Republic of China | 7359013.349 (3743346.065-13428284.846) | 2264.3 (1141.911-4149.138) | 3680593.97 (1737841.066-7161839.03) | 1065.761 (506.395-2055.833) | -2.23 (-2.42 to -2.04) |
| Plurinational State of Bolivia | 4208.577 (2452.157-7202.928) | 281.234 (163.101-485.574) | 6746.156 (3804.488-11914.919) | 218.827 (123.309-386.709) | -0.54 (-0.69 to -0.4) |
| Portuguese Republic | 7206.539 (3364.927-14032.531) | 286.087 (133.737-556.938) | 4298.723 (1938.361-8369.542) | 171.626 (78.878-333.165) | -1.34 (-1.59 to -1.09) |
| Principality of Andorra | 49.672 (22.919-96.7) | 329.376 (152.695-639.553) | 46.903 (20.764-93.111) | 217.156 (97.354-428.725) | -1.29 (-1.49 to -1.09) |
| Principality of Monaco | 17.341 (8.446-33.079) | 252.139 (124.727-475.252) | 10.4 (4.923-20.249) | 125.942 (59.901-244.49) | -2.28 (-2.69 to -1.86) |
| Puerto Rico | 1807.434 (809.038-3513.52) | 188.809 (84.448-366.987) | 649.974 (304.792-1243.614) | 80.795 (38.16-154.525) | -2.65 (-2.92 to -2.38) |
| Republic of Albania | 3712.063 (1989.754-6661.691) | 443.639 (237.218-801.673) | 1371.983 (721.201-2544.485) | 214.69 (112.985-398.138) | -2.29 (-2.68 to -1.91) |
| Republic of Angola | 58734.85 (26881.31-114078.166) | 2531.802 (1148.7-4970.503) | 158239.175 (72127.134-302867.855) | 2095.582 (933.997-4048.254) | -0.34 (-0.44 to -0.24) |
| Republic of Armenia | 5546.66 (2888.607-10109.408) | 633.171 (328.726-1155.685) | 3531.636 (1867.791-6642.693) | 430.194 (228.034-806.496) | -0.94 (-1.13 to -0.75) |
| Republic of Austria | 5309.859 (2501.032-10327.315) | 268.437 (127.118-521.434) | 4093.494 (1900.679-8008.343) | 196.156 (91.592-382.914) | -0.8 (-0.96 to -0.64) |
| Republic of Azerbaijan | 19547.849 (10842.956-34648.254) | 1041.36 (577.279-1860.597) | 25357.906 (13890.569-45489.556) | 873.257 (480.092-1561.331) | -0.45 (-0.56 to -0.35) |
| Republic of Belarus | 12310.566 (5376.704-24453.019) | 482.382 (211.313-955.53) | 4889.064 (2095.043-10076.897) | 193.812 (83.878-397.233) | -2.11 (-2.47 to -1.76) |
| Republic of Benin | 12813.961 (7114.336-22423.393) | 1186.308 (655.35-2095.316) | 33425.361 (18819.536-58600.286) | 1089.648 (609.457-1918.117) | -0.22 (-0.32 to -0.13) |
| Republic of Botswana | 5552.167 (2686.549-10554.868) | 1704.441 (803.067-3269.854) | 5876.037 (2676.874-11265.371) | 850.551 (387.204-1633.177) | -2.35 (-2.61 to -2.08) |
| Republic of Bulgaria | 17182.465 (7848.612-33557.008) | 842.804 (389.088-1637.824) | 5023.899 (2210.331-10237.573) | 290.818 (129.174-587.294) | -2.79 (-3.17 to -2.41) |
| Republic of Burundi | 12438.493 (6016.046-23166.506) | 971.409 (464.368-1822.825) | 22086.211 (10222.382-42775.008) | 738.597 (338.818-1436.617) | -0.6 (-0.77 to -0.42) |
| Republic of Cabo Verde | 1323.659 (654.941-2486.123) | 1657.625 (815.335-3128.139) | 1919.367 (967.98-3530.245) | 1260.979 (635.778-2319.991) | -0.81 (-0.89 to -0.73) |
| Republic of Cameroon | 41438.61 (20832.187-75418.664) | 1748.542 (862.891-3208.669) | 103773.406 (52997.864-190012.193) | 1376.636 (696.917-2540.243) | -0.41 (-0.53 to -0.3) |
| Republic of Chad | 32996.594 (15659.599-61283.496) | 2486.382 (1159.762-4653.664) | 81353.189 (38334.247-156243.307) | 2115.725 (983.108-4090.674) | -0.25 (-0.35 to -0.15) |
| Republic of Chile | 7368.82 (4026.986-13280.262) | 203.313 (110.717-367.038) | 8648.961 (4607.351-15733.495) | 176.495 (94.142-320.641) | -0.21 (-0.33 to -0.09) |
| Republic of Colombia | 139704.726 (60990.024-277751.873) | 1566.099 (676.241-3142.517) | 105218.857 (42832.581-212288.04) | 795.054 (324.386-1602.948) | -1.97 (-2.28 to -1.66) |
| Republic of Costa Rica | 2631.387 (1131.562-5375.216) | 329.97 (141.255-679.924) | 2723.404 (1148.988-5500.794) | 202.008 (85.421-407.47) | -1.34 (-1.56 to -1.11) |
| Republic of Croatia | 4878.642 (2504.001-9015.219) | 405.201 (209.051-745.703) | 2503.854 (1269.145-4684.869) | 254.977 (130.068-475.673) | -1.46 (-1.73 to -1.19) |
| Republic of Cuba | 5380.701 (2351.951-10899.963) | 173.298 (75.355-352.025) | 1682.733 (662.27-3495.687) | 61.852 (24.641-127.919) | -3.1 (-3.47 to -2.72) |
| Republic of Cyprus | 878.213 (431.61-1649.439) | 443.576 (218.655-832.013) | 846.29 (421.726-1589.519) | 195.688 (98.106-366.233) | -2.75 (-3.02 to -2.47) |
| Republic of C么te d'Ivoire | 56180.311 (28517.523-101637.06) | 2059.677 (1032.852-3771.213) | 97809.95 (48979.663-180600.399) | 1507.75 (747.865-2792.395) | -0.73 (-0.86 to -0.6) |
| Republic of Djibouti | 1228.459 (592.204-2347.241) | 1238.306 (590.235-2393.884) | 3586.52 (1633.542-6909.934) | 1109.241 (505.377-2136.281) | -0.46 (-0.51 to -0.4) |
| Republic of Ecuador | 7679.848 (4366.329-13368.204) | 310.068 (175.374-544.886) | 11450.276 (6454.324-20202.718) | 243.483 (137.156-429.932) | -0.5 (-0.66 to -0.34) |
| Republic of El Salvador | 4605.394 (1938.704-9383.462) | 343.196 (139.519-710.058) | 3422.918 (1380.276-7094.136) | 192.244 (77.482-398.624) | -1.61 (-1.84 to -1.39) |
| Republic of Equatorial Guinea | 996.035 (447.864-1925.028) | 1001.206 (441.482-1948.097) | 2457.519 (1222.366-4598.402) | 671.922 (329.893-1265.478) | -1.3 (-1.37 to -1.24) |
| Republic of Estonia | 2067.124 (986.952-3946.709) | 543.392 (260.819-1032.168) | 892.865 (396.9-1783.651) | 290.261 (129.976-577.557) | -1.86 (-2.08 to -1.64) |
| Republic of Fiji | 2729.117 (1326.681-5118.326) | 1386.089 (669.682-2609.665) | 1345.645 (640.476-2638.842) | 583.486 (277.713-1143.312) | -2.92 (-3.24 to -2.6) |
| Republic of Finland | 3532.906 (1632.016-6884.289) | 283.068 (132.468-548.249) | 3030.592 (1410.574-5868.345) | 270.047 (127.215-519.235) | -0.11 (-0.15 to -0.08) |
| Republic of Ghana | 94703.532 (47349.601-173698.128) | 2652.332 (1305.669-4903.248) | 163603.158 (79467.245-312419.603) | 1850.2 (891.688-3547.309) | -0.77 (-0.96 to -0.58) |
| Republic of Guatemala | 12285.049 (5399.207-24433.371) | 653.143 (281.606-1316.121) | 20451.525 (8657.975-42162.019) | 468.944 (197.368-972.334) | -0.85 (-0.97 to -0.72) |
| Republic of Guinea | 35159.91 (17746.201-65800.36) | 2567.948 (1290.276-4828.791) | 77039.307 (38460.082-142300.735) | 2317.545 (1144.477-4304.251) | -0.41 (-0.53 to -0.28) |
| Republic of Guinea-Bissau | 7276.751 (3610.053-13491.884) | 3111.303 (1516.755-5821.094) | 13492.369 (6522.044-25420.434) | 2546.258 (1220.271-4828.676) | -0.56 (-0.61 to -0.51) |
| Republic of Guyana | 732.084 (349.88-1387.555) | 352.02 (165.985-676.969) | 410.602 (193.382-810.744) | 205.551 (96.496-406.185) | -0.82 (-1.08 to -0.57) |
| Republic of Haiti | 5227.334 (2527.639-9890.504) | 336.352 (160.427-640.225) | 10375.18 (5038.709-19838.208) | 291.072 (141.015-557.704) | -0.28 (-0.35 to -0.2) |
| Republic of Honduras | 5472.621 (2419.713-10728.315) | 511.075 (219.422-1015.181) | 8088.888 (3371.712-16500.211) | 298.62 (123.586-611.215) | -1.3 (-1.6 to -1) |
| Republic of Iceland | 138.956 (62.897-273.828) | 212.574 (96.271-419.274) | 151.092 (70.937-295.145) | 191.533 (90.799-372.436) | -0.23 (-0.31 to -0.15) |
| Republic of India | 1702281.931 (870322.179-3125296.904) | 838.694 (425.035-1548.125) | 2613010.551 (1400706.768-4756143.13) | 688.555 (368.906-1254.351) | -0.74 (-0.77 to -0.71) |
| Republic of Indonesia | 686903.956 (367268.054-1243324.458) | 1429.799 (756.698-2601.828) | 705998.984 (379882.138-1297716.519) | 921.707 (497.273-1691.778) | -1.43 (-1.65 to -1.21) |
| Republic of Iraq | 15169.164 (8749.301-26055.762) | 385.581 (220.84-671.264) | 27535.468 (15821.915-48597.359) | 272.895 (156.367-482.648) | -1.03 (-1.18 to -0.87) |
| Republic of Italy | 111829.893 (57629.181-204941.522) | 784.534 (405.596-1435.089) | 32465.868 (15493.927-63306.055) | 215.709 (103.611-417.717) | -4.21 (-4.57 to -3.86) |
| Republic of Kazakhstan | 18514.566 (10022.173-33255.529) | 445.804 (240.913-801.103) | 14587.402 (7680.95-26688.12) | 283.267 (149.385-517.601) | -1.11 (-1.31 to -0.91) |
| Republic of Kenya | 91193.104 (48027.841-166583.971) | 1764.667 (913.665-3253.231) | 122446.495 (61800.631-231474.298) | 997.287 (499.448-1896.252) | -1.34 (-1.61 to -1.06) |
| Republic of Kiribati | 546.638 (278.766-993.266) | 2905.643 (1465.278-5313.796) | 560.553 (278.664-1045.309) | 1792.569 (888.526-3348.103) | -1.42 (-1.58 to -1.26) |
| Republic of Korea | 164193.24 (81751.031-304079.242) | 1279.254 (637.232-2368.304) | 55357.441 (28899.816-103046.456) | 399.187 (208.229-742.374) | -4 (-4.21 to -3.78) |
| Republic of Latvia | 2206.088 (937.769-4439.99) | 341.901 (146.316-685.751) | 698.068 (291.179-1457.652) | 161.93 (67.911-335.519) | -1.93 (-2.2 to -1.66) |
| Republic of Liberia | 15029.221 (7039.311-27733.383) | 2703.786 (1255.228-5013.196) | 36085.073 (18599.512-66466.297) | 2604.319 (1330.527-4822.051) | -0.32 (-0.41 to -0.23) |
| Republic of Lithuania | 4126.999 (1790.177-8092.933) | 447.853 (195.241-876.272) | 1369.915 (604.05-2729.225) | 220.597 (97.823-439.425) | -1.95 (-2.22 to -1.68) |
| Republic of Madagascar | 43005.685 (19119.369-84446.674) | 1567.331 (685.063-3114.705) | 89099.822 (38747.079-176178.209) | 1271.654 (550.264-2527.902) | -0.4 (-0.52 to -0.28) |
| Republic of Malawi | 29845.392 (13627.97-57584.483) | 1301.038 (579.249-2535.544) | 45335.835 (20623.3-86666.784) | 964.447 (434.195-1855.568) | -0.96 (-1.09 to -0.83) |
| Republic of Maldives | 417.392 (184.442-812.208) | 863.309 (371.143-1703.036) | 491.589 (208.522-996.492) | 382.026 (161.991-772.876) | -2.21 (-2.53 to -1.88) |
| Republic of Mali | 32054.538 (15032.099-61531.787) | 1663.903 (767.577-3227.3) | 74729.729 (35705.51-142531.017) | 1423.585 (670.291-2742.151) | -0.53 (-0.68 to -0.38) |
| Republic of Malta | 189.577 (83.884-378.291) | 202.939 (90.505-403.918) | 120.806 (51.733-247.122) | 115.905 (50.231-235.062) | -1.4 (-1.65 to -1.15) |
| Republic of Mauritius | 2252.751 (963.555-4524.017) | 741.064 (317.147-1492.298) | 1466.09 (599.829-2978.455) | 447.038 (184.034-906.216) | -1.35 (-1.65 to -1.06) |
| Republic of Moldova | 28796.818 (14099.359-53898.642) | 2539.423 (1245.285-4748.902) | 11964.713 (5690.856-23320.337) | 1140.31 (547.948-2209.889) | -2.01 (-2.3 to -1.72) |
| Republic of Mozambique | 75954.622 (36489.997-141096.272) | 2417.303 (1147.558-4533.05) | 125968.679 (62708.919-234078.631) | 1794.474 (882.701-3348.694) | -0.67 (-0.8 to -0.55) |
| Republic of Namibia | 5930.09 (2945.253-11106.245) | 1727.852 (844.545-3279.724) | 9333.871 (4805.632-17405.121) | 1401.853 (716.975-2630.653) | -0.55 (-0.6 to -0.5) |
| Republic of Nauru | 54.816 (28.024-100.182) | 2247.191 (1140.379-4131.441) | 24.27 (11.762-45.657) | 912.48 (439.878-1720.655) | -3.04 (-3.3 to -2.77) |
| Republic of Nicaragua | 1300.678 (626.285-2496.394) | 142.368 (67.921-274.885) | 1607.163 (724.433-3109.592) | 88.844 (39.957-172.098) | -1.34 (-1.62 to -1.06) |
| Republic of Niue | 8.102 (4.057-15.304) | 1644.248 (819.111-3113.573) | 1.682 (0.805-3.214) | 389.65 (187.247-743.72) | -3.82 (-4.31 to -3.33) |
| Republic of Palau | 75.185 (38.809-135.703) | 1827.504 (937.466-3309.381) | 34.608 (16.777-66.008) | 782.802 (381.49-1498.135) | -2.9 (-3.23 to -2.56) |
| Republic of Panama | 1565.651 (648.056-3197.733) | 250.699 (102.302-516.22) | 1605.589 (688.798-3262.28) | 151.323 (64.982-307.388) | -0.95 (-1.16 to -0.73) |
| Republic of Paraguay | 3907.812 (1807.501-7470.951) | 411.821 (188.7-794.156) | 5939.022 (2793.167-11524.472) | 311.705 (146.465-604.941) | -0.75 (-0.91 to -0.59) |
| Republic of Peru | 37512.588 (19385.523-67919.75) | 692.058 (354-1264.242) | 57741.85 (30040.849-105876.639) | 590.329 (307.461-1081.33) | -0.5 (-0.59 to -0.41) |
| Republic of Poland | 42412.294 (24235.803-74398.722) | 447.882 (256.746-781.165) | 12168.046 (6186.949-22795.681) | 117.077 (59.792-218.788) | -4.16 (-4.42 to -3.91) |
| Republic of Rwanda | 17123.114 (7136.654-35028.103) | 1032.387 (420.715-2145.111) | 25558.08 (9639.629-53295.509) | 745.543 (276.94-1563.199) | -0.65 (-0.83 to -0.46) |
| Republic of San Marino | 17.893 (8.759-33.237) | 286.596 (140.451-532.11) | 12.163 (5.921-22.951) | 151.015 (73.906-285.681) | -1.77 (-2.04 to -1.49) |
| Republic of Senegal | 39089.943 (18934.036-73265.982) | 2270.632 (1078.191-4291.816) | 71156.708 (33674-136852.13) | 1888.722 (887.526-3652.132) | -0.65 (-0.76 to -0.54) |
| Republic of Serbia | 7832.601 (3730.062-14804.244) | 336.016 (160.528-633.933) | 5156.548 (2429.808-9761.845) | 248.038 (117.588-468.819) | -0.72 (-0.85 to -0.6) |
| Republic of Seychelles | 193.618 (95.599-370.047) | 1059.075 (517.506-2036.572) | 136.417 (67.269-260.322) | 522.247 (258.133-996.444) | -1.98 (-2.32 to -1.63) |
| Republic of Sierra Leone | 20209.383 (9779.674-37366.679) | 2004.784 (959.419-3733.427) | 39733.537 (18585.28-74777.315) | 1749.781 (812.468-3313.118) | -0.41 (-0.51 to -0.3) |
| Republic of Singapore | 11462.344 (6144.235-20596.735) | 1213.55 (651.511-2178.747) | 9199.9 (4908.831-16992.291) | 492.661 (264.818-906.364) | -2.99 (-3.28 to -2.69) |
| Republic of Slovenia | 2353.272 (1110.444-4448.375) | 473.577 (224.346-891.887) | 1313.779 (608.903-2594.463) | 285.093 (133.683-558.435) | -1.41 (-1.66 to -1.15) |
| Republic of South Africa | 67414.275 (35476.048-120924.615) | 692.807 (360.629-1249.18) | 44839.933 (22946.704-84068.655) | 280.812 (144.039-525.322) | -3.02 (-3.32 to -2.73) |
| Republic of South Sudan | 19410.66 (8894.828-37457.954) | 1487.988 (667.301-2888.344) | 32989.473 (16010.138-62621.423) | 1434.849 (683.977-2734.031) | -0.09 (-0.12 to -0.05) |
| Republic of Sudan | 74767.066 (35013.658-142307.834) | 1594.849 (733.801-3055.169) | 134976.407 (68125.969-250931.153) | 1213.1 (607.464-2263.612) | -0.95 (-1.03 to -0.87) |
| Republic of Suriname | 240.845 (107.438-479.737) | 244.377 (107.505-490.201) | 272.322 (119.71-535.68) | 186.661 (82.142-367.041) | -0.71 (-0.88 to -0.53) |
| Republic of Tajikistan | 10870.435 (5421.756-19907.781) | 894.186 (441.217-1658.82) | 17987.695 (8996.517-33797.998) | 699.902 (349.076-1318.47) | -0.63 (-0.74 to -0.52) |
| Republic of Trinidad and Tobago | 729.299 (323.633-1481.016) | 232.943 (102.601-475.104) | 604.613 (277.478-1200.681) | 176.451 (81.411-349.019) | -0.74 (-0.84 to -0.65) |
| Republic of Tunisia | 28617.908 (14186.477-52289.119) | 1384.53 (677.403-2552.182) | 21539.467 (11041.716-40208.532) | 630.322 (323.89-1175.026) | -2.3 (-2.54 to -2.06) |
| Republic of Turkey | 135757 (72713.592-239709.528) | 956.805 (509.794-1701.475) | 130459.315 (71511.289-232256.95) | 583.631 (320.587-1037.732) | -1.46 (-1.61 to -1.32) |
| Republic of Uganda | 56648.072 (26489.346-105858.418) | 1457.498 (665.102-2763.051) | 123101.647 (60968.25-229307.887) | 1243.963 (611.37-2332.033) | -0.43 (-0.52 to -0.35) |
| Republic of Uzbekistan | 85525.386 (41641.624-159027.541) | 1719.744 (821.579-3226.213) | 116861.057 (56512.141-221825.369) | 1270.509 (615.414-2412.107) | -0.94 (-1.11 to -0.77) |
| Republic of Vanuatu | 913.491 (444.089-1712.336) | 2594.046 (1244.158-4900.765) | 1116.303 (551.367-2114.475) | 1485.092 (729.187-2825.098) | -1.63 (-1.78 to -1.49) |
| Republic of Yemen | 60646.755 (28973.372-113609.559) | 2193.52 (1029.501-4143.628) | 131334.2 (66251.534-241945.825) | 1620.914 (813.757-3003.49) | -0.93 (-0.99 to -0.87) |
| Republic of Zambia | 23487.133 (11554.617-43198.109) | 1277.84 (614.75-2388.782) | 48496.052 (25273.571-89242.085) | 1024.061 (529.567-1898.366) | -0.62 (-0.72 to -0.51) |
| Republic of Zimbabwe | 117087.27 (57713.71-216195.982) | 4874.365 (2350.256-9137.867) | 145194.964 (70193.813-274010.344) | 3734.095 (1791.016-7086.511) | -0.7 (-0.88 to -0.51) |
| Republic of the Congo | 11384.372 (5097.578-22061.052) | 1995.992 (873.212-3910.416) | 22789.112 (10392.679-44618.723) | 1588.471 (718.891-3124.604) | -0.54 (-0.6 to -0.48) |
| Republic of the Gambia | 4484.732 (2318.605-8202.606) | 1973.447 (1007.084-3633.051) | 4824.958 (2423.479-8931.383) | 922.499 (458.059-1717.647) | -2.44 (-2.66 to -2.21) |
| Republic of the Marshall Islands | 193.122 (97.645-349.493) | 1965.963 (972.166-3605.491) | 160.973 (77.534-302.907) | 1108.364 (532.792-2087.569) | -1.71 (-1.95 to -1.47) |
| Republic of the Niger | 50652.099 (23179.276-98331.422) | 2902.003 (1304.365-5692.508) | 138036.869 (67423.942-256835.969) | 2603.137 (1236.255-4929.99) | -0.08 (-0.16 to 0.01) |
| Republic of the Philippines | 543127.18 (283848.627-993668.151) | 3484.93 (1797.852-6420.133) | 591697.573 (300887.615-1102259.293) | 2061.703 (1045.953-3848.722) | -1.53 (-1.69 to -1.36) |
| Republic of the Union of Myanmar | 42859.416 (19315.377-84472.247) | 401.055 (180.088-793.284) | 46198.341 (21099.127-92501.766) | 306.861 (140.148-614.623) | -0.7 (-0.81 to -0.59) |
| Romania | 60118.794 (30440.11-111979.639) | 1072.703 (543.027-1997.256) | 21902.127 (10708.558-41777.102) | 457.662 (225.524-870.567) | -2.43 (-2.71 to -2.15) |
| Russian Federation | 236375.852 (114046.393-442067.487) | 633.81 (307.78-1181.95) | 142017.009 (66953.742-274483.265) | 392.937 (186.694-752.775) | -1.12 (-1.55 to -0.68) |
| Saint Kitts and Nevis | 25.388 (10.736-51.494) | 244.627 (102.018-501.111) | 23.793 (10.456-48.729) | 145.657 (64.229-298.103) | -1.63 (-1.95 to -1.31) |
| Saint Lucia | 98.613 (47.013-188.489) | 284.544 (133.79-547.898) | 94.534 (44.405-185.935) | 202.188 (95.519-397.275) | -0.79 (-0.95 to -0.64) |
| Saint Vincent and the Grenadines | 54.184 (22.871-111.47) | 198.769 (82.626-413.259) | 40.713 (17.442-82.443) | 145.996 (62.743-294.678) | -0.76 (-0.93 to -0.58) |
| Slovak Republic | 6555.249 (3257.313-12475.792) | 494.98 (246.729-939.282) | 4052.837 (1849.765-7927.69) | 285.864 (132.238-554.556) | -1.51 (-1.8 to -1.23) |
| Socialist Republic of Viet Nam | 378070.743 (188307.486-705221.599) | 2163.195 (1064.916-4062.356) | 441408.233 (216504.224-829412.534) | 1665.267 (822.007-3117.684) | -0.7 (-0.85 to -0.54) |
| Solomon Islands | 2316.261 (1168.404-4208.073) | 3087.071 (1519.517-5678.464) | 2363.229 (1085.411-4604.703) | 1455.372 (661.004-2848.435) | -2.35 (-2.62 to -2.07) |
| State of Eritrea | 14351.247 (6631.107-27562.4) | 1805.286 (823.106-3512.247) | 21589.688 (10040.73-41718.148) | 1335.778 (616.492-2584.475) | -0.73 (-0.87 to -0.58) |
| State of Israel | 2160.897 (1140.781-3978.387) | 177.359 (93.473-326.696) | 2054.499 (1048.849-3808.314) | 89.808 (45.857-166.537) | -2.17 (-2.51 to -1.84) |
| State of Kuwait | 1356.954 (726.274-2435.379) | 323.842 (173.038-583.354) | 2359.682 (1241.251-4327.732) | 128.568 (67.946-235.469) | -2.9 (-3.22 to -2.57) |
| State of Libya | 6873.286 (3730.42-12237.819) | 771.396 (413.262-1391.48) | 10485.018 (5640.902-19140.132) | 508.115 (273.928-925.951) | -1.09 (-1.22 to -0.96) |
| State of Qatar | 903.08 (520.666-1573.247) | 1134.876 (655.619-1978.69) | 3461.737 (1951.769-6107.63) | 548.906 (311.829-965.104) | -2.36 (-2.74 to -1.98) |
| Sultanate of Oman | 4237.118 (2230.183-7518.342) | 1249.412 (649.56-2233.732) | 4211.159 (2119.92-7853.085) | 396.148 (199.744-736.603) | -3.34 (-3.82 to -2.86) |
| Swiss Confederation | 4456.421 (2169.413-8593.548) | 253.397 (124.573-485.904) | 4567.374 (2206.944-8775.87) | 235.113 (115.426-446.891) | -0.31 (-0.36 to -0.26) |
| Syrian Arab Republic | 37066.007 (20818.015-65417.182) | 1375.278 (765.252-2459.258) | 32060.679 (17837.508-57400.012) | 870.88 (482.016-1563.031) | -1.31 (-1.55 to -1.08) |
| Taiwan (Province of China) | 206928.753 (109173.031-375277.867) | 3683.773 (1937.647-6680.299) | 66798.302 (35348.533-120306.946) | 931.14 (496.584-1671.879) | -3.72 (-4.16 to -3.27) |
| Togolese Republic | 22056.73 (10564.145-41811.791) | 2565.306 (1208.85-4926.756) | 46787.608 (22697.249-86899.87) | 2161.528 (1040.822-4028.25) | -0.59 (-0.68 to -0.5) |
| Tokelau | 8.379 (4.362-15.409) | 2384.398 (1233.409-4399.442) | 3.747 (1.944-7.017) | 1176.239 (610.897-2204.08) | -2.01 (-2.33 to -1.7) |
| Turkmenistan | 7325.373 (3748.227-13474.133) | 816.883 (411.684-1516.137) | 7911.766 (3971.47-15000.962) | 626.696 (314.117-1188.576) | -0.83 (-0.93 to -0.73) |
| Tuvalu | 59.855 (29.603-109.171) | 2447.864 (1207.577-4469.246) | 42.948 (21.279-82.811) | 1536.061 (757.966-2970.049) | -1.22 (-1.41 to -1.03) |
| Ukraine | 40394.054 (18693.981-78354.937) | 319.403 (148.775-617.51) | 27158.658 (12255.651-52993.982) | 256.467 (117.937-496.871) | -0.19 (-0.53 to 0.14) |
| Union of the Comoros | 1443.985 (658.14-2747.916) | 1354.493 (603.137-2604.194) | 1974.442 (866.976-3899.186) | 1020.149 (444.186-2018.05) | -0.58 (-0.71 to -0.46) |
| United Arab Emirates | 1385.429 (783.575-2430.454) | 400.752 (226.673-705.128) | 4636.234 (2519.945-8276.868) | 186.116 (102.214-331.214) | -2.33 (-2.62 to -2.03) |
| United Kingdom of Great Britain and Northern Ireland | 39035.839 (18965.595-73291.309) | 275.766 (134.878-516.271) | 42771.387 (21941.275-79300.923) | 281.04 (145.41-517.367) | 0.15 (0.12 to 0.18) |
| United Mexican States | 50058.643 (25270.509-91899.822) | 226.62 (113.208-418.493) | 39326.015 (19034.608-76060.101) | 111.217 (53.868-215.073) | -2.49 (-2.76 to -2.23) |
| United Republic of Tanzania | 83303.273 (39698.574-156701.33) | 1366.662 (632.339-2602.395) | 153376.847 (72860.051-287079.044) | 1076.728 (507.563-2028.247) | -0.61 (-0.77 to -0.46) |
| United States Virgin Islands | 55.914 (25.876-109.91) | 196.783 (91.398-385.974) | 14.989 (6.818-29.48) | 79.617 (36.383-155.906) | -2.93 (-3.14 to -2.72) |
| United States of America | 100648.197 (51605.902-187169.128) | 149.324 (77.086-276.66) | 57117.409 (28273.933-106679.978) | 70.408 (34.956-131.408) | -1.92 (-2.33 to -1.5) |
| (SDI: Socio-Demographic Index, ASR: Age-standardized rate, EAPC: Estimated Annual Percentage Change, UI: uncertainty intervals, CI: confidence intervals, AHB: Acute hepatitis B, ASIR: Age-standardized rate of Incidence) | | | | | |

| **Table S3. Deaths cases and ASMR of AHB in 1990 and 2021 and its trends (National level).** | | | | | |
| --- | --- | --- | --- | --- | --- |
| **location** | **Number (95% UI) 1990** | **ASR (95% UI) 1990** | **Number (95% UI) 2021** | **ASR (95% UI) 2021** | **EAPC (95%CI) 1990–2021** |
| American Samoa | 0.003 (0.001-0.007) | 0.033 (0.013-0.065) | 0.003 (0.001-0.006) | 0.026 (0.012-0.051) | -1.05 (-1.37 to -0.73) |
| Antigua and Barbuda | 0.002 (0.001-0.005) | 0.016 (0.006-0.032) | 0.002 (0.001-0.003) | 0.006 (0.003-0.011) | -3.35 (-4.07 to -2.63) |
| Arab Republic of Egypt | 178.936 (44.406-433.579) | 1.539 (0.388-3.701) | 73.943 (29.004-170.532) | 0.305 (0.12-0.704) | -5.09 (-5.32 to -4.86) |
| Argentine Republic | 0.702 (0.217-1.851) | 0.009 (0.003-0.023) | 0.224 (0.099-0.426) | 0.002 (0.001-0.003) | -5.82 (-6.43 to -5.21) |
| Australia | 0.083 (0.045-0.142) | 0.002 (0.001-0.003) | 0.159 (0.088-0.257) | 0.002 (0.001-0.004) | -1.04 (-2.45 to 0.38) |
| Barbados | 0.016 (0.005-0.037) | 0.024 (0.007-0.055) | 0.023 (0.009-0.045) | 0.03 (0.012-0.06) | 0.88 (0.44 to 1.33) |
| Belize | 0.012 (0.004-0.028) | 0.034 (0.01-0.078) | 0.018 (0.008-0.032) | 0.015 (0.007-0.028) | -2.9 (-3.43 to -2.36) |
| Bermuda | 0 (0-0) | 0.001 (0-0.002) | 0 (0-0) | 0 (0-0.001) | -4.23 (-4.9 to -3.55) |
| Bolivarian Republic of Venezuela | 0.058 (0.016-0.151) | 0.001 (0-0.003) | 0.409 (0.127-0.933) | 0.006 (0.002-0.013) | 5.29 (4.74 to 5.83) |
| Bosnia and Herzegovina | 0.435 (0.194-0.805) | 0.038 (0.017-0.07) | 0.12 (0.056-0.22) | 0.016 (0.008-0.029) | -2.99 (-3.3 to -2.68) |
| Brunei Darussalam | 0.315 (0.135-0.602) | 0.549 (0.236-1.047) | 0.058 (0.03-0.102) | 0.044 (0.023-0.077) | -8.85 (-9.38 to -8.32) |
| Burkina Faso | 19.831 (4.736-43.087) | 0.99 (0.237-2.154) | 27.102 (9.178-56.894) | 0.505 (0.174-1.052) | -2.52 (-3.29 to -1.74) |
| Canada | 0.319 (0.165-0.539) | 0.004 (0.002-0.007) | 0.157 (0.067-0.286) | 0.002 (0.001-0.003) | -4.37 (-5.09 to -3.65) |
| Central African Republic | 11.558 (1.407-31.996) | 1.981 (0.241-5.44) | 10.887 (2.383-26.607) | 0.835 (0.182-2.043) | -2.69 (-2.95 to -2.42) |
| Commonwealth of Dominica | 0.006 (0.002-0.014) | 0.039 (0.013-0.09) | 0.004 (0.002-0.008) | 0.027 (0.012-0.051) | -1.44 (-1.58 to -1.29) |
| Commonwealth of the Bahamas | 0.03 (0.011-0.063) | 0.043 (0.016-0.09) | 0.029 (0.013-0.053) | 0.027 (0.012-0.048) | -1.89 (-2.69 to -1.08) |
| Cook Islands | 0 (0-0) | 0 (0-0) | 0 (0-0) | 0 (0-0) | -4.77 (-4.97 to -4.56) |
| Czech Republic | 0.31 (0.159-0.527) | 0.012 (0.006-0.02) | 0.223 (0.127-0.358) | 0.01 (0.005-0.015) | -1.58 (-2.78 to -0.36) |
| Democratic People's Republic of Korea | 22.513 (6.242-51.178) | 0.416 (0.116-0.942) | 3.935 (1.613-7.617) | 0.056 (0.023-0.108) | -6.42 (-7.05 to -5.78) |
| Democratic Republic of Sao Tome and Principe | 0.071 (0.024-0.15) | 0.307 (0.108-0.643) | 0.059 (0.023-0.127) | 0.113 (0.045-0.242) | -3.75 (-4.48 to -3.01) |
| Democratic Republic of Timor-Leste | 0.018 (0.001-0.085) | 0.011 (0.001-0.049) | 0.028 (0.002-0.106) | 0.009 (0.001-0.032) | -1.64 (-2.24 to -1.04) |
| Democratic Republic of the Congo | 9.14 (1.892-22.978) | 0.115 (0.024-0.29) | 19.528 (4.685-42.901) | 0.096 (0.023-0.21) | -0.43 (-0.78 to -0.07) |
| Democratic Socialist Republic of Sri Lanka | 2.046 (0.407-5.608) | 0.047 (0.01-0.127) | 0.669 (0.173-1.566) | 0.011 (0.003-0.027) | -5.66 (-6.18 to -5.14) |
| Dominican Republic | 0.165 (0.061-0.344) | 0.009 (0.003-0.019) | 0.674 (0.271-1.345) | 0.023 (0.009-0.046) | 1.92 (0.92 to 2.93) |
| Eastern Republic of Uruguay | 0.022 (0.008-0.049) | 0.003 (0.001-0.006) | 0.01 (0.004-0.019) | 0.001 (0-0.002) | -4.88 (-5.5 to -4.25) |
| Federal Democratic Republic of Ethiopia | 84.868 (16.713-231.828) | 0.819 (0.162-2.215) | 94.38 (20.697-222.747) | 0.356 (0.081-0.841) | -3.68 (-4.1 to -3.25) |
| Federal Democratic Republic of Nepal | 15.334 (1.111-66.885) | 0.345 (0.025-1.505) | 43.183 (7.068-138.298) | 0.482 (0.079-1.543) | 1.4 (1.25 to 1.55) |
| Federal Republic of Germany | 2.98 (1.498-5.151) | 0.015 (0.008-0.026) | 0.204 (0.082-0.399) | 0.001 (0-0.002) | -8.37 (-9.07 to -7.67) |
| Federal Republic of Nigeria | 595.597 (145.7-1354.273) | 3.302 (0.8-7.557) | 254.756 (105.293-500.049) | 0.471 (0.196-0.927) | -6.91 (-7.47 to -6.35) |
| Federal Republic of Somalia | 49.499 (4.07-141.164) | 3.183 (0.265-9.134) | 127.534 (17.401-309.318) | 2.812 (0.381-6.813) | -0.2 (-0.41 to 0) |
| Federated States of Micronesia | 0.006 (0.001-0.018) | 0.027 (0.004-0.084) | 0.003 (0.001-0.007) | 0.01 (0.002-0.03) | -3.08 (-3.19 to -2.96) |
| Federative Republic of Brazil | 5.251 (3.926-7.118) | 0.015 (0.011-0.02) | 8.641 (7.183-10.371) | 0.014 (0.012-0.017) | -1.14 (-2.1 to -0.16) |
| French Republic | 0.661 (0.362-1.088) | 0.005 (0.003-0.008) | 0.267 (0.11-0.521) | 0.002 (0.001-0.004) | -5.02 (-6.09 to -3.93) |
| Gabonese Republic | 0.954 (0.274-2.109) | 0.479 (0.139-1.059) | 0.399 (0.126-0.946) | 0.086 (0.028-0.204) | -5.3 (-5.71 to -4.9) |
| Georgia | 0.559 (0.247-1.101) | 0.039 (0.017-0.078) | 0.122 (0.075-0.19) | 0.016 (0.01-0.025) | -1.74 (-3.25 to -0.19) |
| Grand Duchy of Luxembourg | 0.002 (0.001-0.003) | 0.002 (0.001-0.003) | 0.001 (0-0.001) | 0 (0-0.001) | -7.5 (-8.86 to -6.11) |
| Greenland | 0.006 (0.002-0.014) | 0.043 (0.014-0.095) | 0.001 (0-0.002) | 0.006 (0.002-0.013) | -7.87 (-8.5 to -7.24) |
| Grenada | 0.035 (0.015-0.075) | 0.188 (0.081-0.404) | 0.02 (0.011-0.033) | 0.077 (0.042-0.13) | -3.22 (-3.74 to -2.71) |
| Guam | 0 (0-0.001) | 0.001 (0-0.004) | 0.007 (0.002-0.014) | 0.018 (0.006-0.038) | 9.49 (7.03 to 12.02) |
| Hashemite Kingdom of Jordan | 2.234 (0.601-5.459) | 0.337 (0.092-0.814) | 0.792 (0.277-1.752) | 0.027 (0.01-0.06) | -8.75 (-9.16 to -8.33) |
| Hellenic Republic | 0.443 (0.273-0.684) | 0.018 (0.011-0.027) | 0.212 (0.11-0.362) | 0.009 (0.005-0.015) | -3.77 (-4.86 to -2.67) |
| Hungary | 0.54 (0.286-0.899) | 0.021 (0.011-0.035) | 0.197 (0.115-0.32) | 0.009 (0.005-0.014) | -3.3 (-4.01 to -2.58) |
| Independent State of Papua New Guinea | 0.554 (0.091-1.904) | 0.062 (0.01-0.217) | 0.354 (0.041-1.221) | 0.014 (0.002-0.048) | -5.26 (-5.53 to -5) |
| Independent State of Samoa | 0.009 (0.003-0.021) | 0.028 (0.009-0.064) | 0.011 (0.004-0.023) | 0.025 (0.01-0.051) | -0.23 (-0.55 to 0.09) |
| Ireland | 0.001 (0.001-0.003) | 0 (0-0) | 0.001 (0-0.002) | 0 (0-0) | -5.7 (-7.19 to -4.19) |
| Islamic Republic of Afghanistan | 156.168 (13.894-402.549) | 7.678 (0.685-19.612) | 175.896 (38.819-425.946) | 2.872 (0.638-6.882) | -3.84 (-4.51 to -3.16) |
| Islamic Republic of Iran | 30.12 (17.113-45.255) | 0.286 (0.167-0.427) | 19.83 (15.2-25.839) | 0.081 (0.062-0.105) | -4.35 (-4.52 to -4.18) |
| Islamic Republic of Mauritania | 20.185 (3.513-52.635) | 4.567 (0.792-12.04) | 5.849 (2.364-12.072) | 0.595 (0.24-1.233) | -6.27 (-6.67 to -5.85) |
| Islamic Republic of Pakistan | 191.789 (40.614-561.202) | 0.933 (0.198-2.736) | 643.537 (199.842-1390.363) | 1.152 (0.358-2.492) | 0.96 (0.75 to 1.16) |
| Jamaica | 0.121 (0.033-0.29) | 0.023 (0.006-0.054) | 0.254 (0.097-0.507) | 0.032 (0.012-0.065) | 0.72 (0.22 to 1.22) |
| Japan | 1.888 (1.613-2.191) | 0.005 (0.004-0.006) | 1.694 (1.45-1.937) | 0.006 (0.005-0.006) | -4.35 (-7.29 to -1.31) |
| Kingdom of Bahrain | 0.211 (0.079-0.45) | 0.231 (0.086-0.489) | 0.159 (0.062-0.338) | 0.047 (0.018-0.1) | -5.6 (-6.24 to -4.97) |
| Kingdom of Belgium | 0.258 (0.133-0.444) | 0.011 (0.006-0.018) | 0.097 (0.049-0.165) | 0.004 (0.002-0.006) | -5.1 (-6 to -4.19) |
| Kingdom of Bhutan | 0.858 (0.113-2.769) | 0.637 (0.086-2.016) | 0.744 (0.194-1.725) | 0.363 (0.095-0.842) | -2.44 (-3.1 to -1.77) |
| Kingdom of Cambodia | 33.802 (5.654-99.727) | 1.453 (0.247-4.277) | 13.258 (3.581-31.894) | 0.301 (0.082-0.724) | -5.74 (-6.54 to -4.93) |
| Kingdom of Denmark | 0.07 (0.033-0.123) | 0.005 (0.002-0.009) | 0.012 (0.004-0.024) | 0.001 (0-0.002) | -8.11 (-9.15 to -7.07) |
| Kingdom of Eswatini | 1.137 (0.375-2.453) | 0.706 (0.24-1.513) | 0.519 (0.17-1.163) | 0.189 (0.064-0.421) | -3.94 (-4.69 to -3.18) |
| Kingdom of Lesotho | 2.21 (0.656-4.846) | 0.649 (0.196-1.408) | 1.665 (0.55-3.513) | 0.382 (0.128-0.799) | -0.42 (-1.35 to 0.53) |
| Kingdom of Morocco | 11.241 (2.743-27.728) | 0.212 (0.053-0.516) | 13.503 (5.094-27.71) | 0.136 (0.051-0.279) | -1.58 (-1.78 to -1.39) |
| Kingdom of Norway | 0.005 (0.003-0.006) | 0 (0-0.001) | 0.012 (0.009-0.015) | 0.001 (0.001-0.001) | 1.65 (0.04 to 3.3) |
| Kingdom of Saudi Arabia | 19.763 (5.037-45.961) | 0.745 (0.189-1.731) | 7.882 (3.636-14.744) | 0.074 (0.034-0.138) | -6.8 (-7.46 to -6.13) |
| Kingdom of Spain | 0.161 (0.085-0.276) | 0.002 (0.001-0.003) | 0.045 (0.02-0.083) | 0 (0-0.001) | -6.47 (-7.86 to -5.07) |
| Kingdom of Sweden | 0.007 (0.004-0.012) | 0 (0-0.001) | 0.013 (0.007-0.021) | 0.001 (0-0.001) | 0.86 (-1.16 to 2.92) |
| Kingdom of Thailand | 21.014 (9.173-42.071) | 0.142 (0.063-0.284) | 10.562 (5.111-19.346) | 0.057 (0.027-0.104) | -2.61 (-3.63 to -1.57) |
| Kingdom of Tonga | 0.277 (0.092-0.599) | 1.398 (0.453-3.059) | 0.059 (0.017-0.146) | 0.246 (0.069-0.606) | -5.72 (-5.92 to -5.52) |
| Kingdom of the Netherlands | 0.075 (0.038-0.127) | 0.002 (0.001-0.003) | 0.019 (0.007-0.038) | 0 (0-0.001) | -6.83 (-8.09 to -5.54) |
| Kyrgyz Republic | 11.693 (7.127-19.984) | 1.047 (0.631-1.801) | 1.585 (0.97-2.525) | 0.091 (0.056-0.145) | -7.03 (-7.56 to -6.5) |
| Lao People's Democratic Republic | 1.809 (0.22-6.12) | 0.207 (0.025-0.7) | 1.095 (0.268-2.93) | 0.057 (0.014-0.152) | -4.81 (-5.61 to -4) |
| Lebanese Republic | 0.501 (0.14-1.184) | 0.074 (0.021-0.173) | 0.27 (0.127-0.501) | 0.017 (0.008-0.032) | -4.89 (-5.12 to -4.65) |
| Malaysia | 12.191 (5.905-22.045) | 0.301 (0.147-0.543) | 11.635 (6.017-20.669) | 0.142 (0.074-0.252) | -2.39 (-2.55 to -2.24) |
| Mongolia | 6.202 (2.564-12.029) | 1.144 (0.473-2.202) | 1 (0.536-1.67) | 0.12 (0.064-0.201) | -7.64 (-8.04 to -7.23) |
| Montenegro | 0.004 (0.002-0.008) | 0.003 (0.001-0.005) | 0.002 (0.001-0.004) | 0.001 (0.001-0.003) | -1.94 (-2.71 to -1.15) |
| New Zealand | 0.131 (0.088-0.185) | 0.015 (0.01-0.021) | 0.009 (0.006-0.014) | 0.001 (0-0.001) | -11.06 (-13.35 to -8.71) |
| North Macedonia | 0.112 (0.05-0.213) | 0.022 (0.01-0.042) | 0.058 (0.023-0.127) | 0.011 (0.004-0.024) | -2.79 (-3.71 to -1.87) |
| Northern Mariana Islands | 0.007 (0.003-0.013) | 0.057 (0.024-0.11) | 0.006 (0.003-0.011) | 0.044 (0.022-0.079) | -0.64 (-0.81 to -0.46) |
| Palestine | 0.323 (0.084-0.77) | 0.09 (0.024-0.216) | 0.252 (0.112-0.471) | 0.022 (0.01-0.041) | -4.64 (-4.74 to -4.53) |
| People's Democratic Republic of Algeria | 29.931 (11.355-60.802) | 0.646 (0.249-1.293) | 21.267 (9.37-41.428) | 0.181 (0.08-0.354) | -4.01 (-4.16 to -3.86) |
| People's Republic of Bangladesh | 140.259 (17.831-513.86) | 0.683 (0.087-2.488) | 218.689 (43.432-565.364) | 0.486 (0.096-1.258) | -0.85 (-1.14 to -0.56) |
| People's Republic of China | 1028.64 (513.106-1590.181) | 0.355 (0.178-0.547) | 77.882 (52.518-111.527) | 0.021 (0.014-0.031) | -9.48 (-10.09 to -8.86) |
| Plurinational State of Bolivia | 0.143 (0.029-0.414) | 0.01 (0.002-0.029) | 0.32 (0.095-0.762) | 0.01 (0.003-0.025) | 0.03 (-0.03 to 0.1) |
| Portuguese Republic | 0.333 (0.205-0.513) | 0.013 (0.008-0.02) | 0.122 (0.067-0.203) | 0.005 (0.003-0.008) | -5.07 (-6.01 to -4.11) |
| Principality of Andorra | 0.027 (0.008-0.068) | 0.183 (0.052-0.464) | 0.041 (0.016-0.086) | 0.188 (0.07-0.393) | 0.45 (-0.1 to 0.99) |
| Principality of Monaco | 0 (0-0) | 0.004 (0.002-0.007) | 0 (0-0) | 0.003 (0.001-0.006) | -0.64 (-0.87 to -0.4) |
| Puerto Rico | 0.019 (0.006-0.044) | 0.002 (0.001-0.005) | 0.122 (0.067-0.204) | 0.015 (0.008-0.026) | 5.09 (3 to 7.22) |
| Republic of Albania | 0.24 (0.125-0.414) | 0.028 (0.015-0.048) | 0.078 (0.037-0.144) | 0.013 (0.006-0.023) | -2.35 (-3.18 to -1.53) |
| Republic of Angola | 65.416 (5.491-192.922) | 3.041 (0.252-8.936) | 39.644 (7.045-107.384) | 0.539 (0.096-1.453) | -5.44 (-5.88 to -4.99) |
| Republic of Armenia | 0.886 (0.562-1.288) | 0.096 (0.061-0.14) | 0.036 (0.023-0.054) | 0.005 (0.003-0.008) | -8.2 (-8.65 to -7.75) |
| Republic of Austria | 0.058 (0.031-0.096) | 0.003 (0.002-0.005) | 0.008 (0.003-0.017) | 0 (0-0.001) | -7.47 (-8.91 to -6.01) |
| Republic of Azerbaijan | 7.127 (3.894-11.882) | 0.343 (0.185-0.579) | 1.317 (0.544-2.541) | 0.049 (0.02-0.095) | -6.56 (-6.87 to -6.25) |
| Republic of Belarus | 4.511 (2.852-6.727) | 0.185 (0.117-0.275) | 0.785 (0.41-1.352) | 0.039 (0.02-0.068) | -4.93 (-5.86 to -3.99) |
| Republic of Benin | 6.392 (1.961-14.026) | 0.62 (0.189-1.351) | 5.901 (2.122-13.766) | 0.194 (0.071-0.451) | -4.08 (-4.56 to -3.6) |
| Republic of Botswana | 1.065 (0.363-2.358) | 0.411 (0.143-0.894) | 0.48 (0.202-0.941) | 0.072 (0.031-0.142) | -4.4 (-5.18 to -3.62) |
| Republic of Bulgaria | 0.43 (0.233-0.717) | 0.021 (0.012-0.035) | 0.46 (0.252-0.767) | 0.032 (0.017-0.054) | -0.03 (-1.06 to 1.01) |
| Republic of Burundi | 1.318 (0.113-5.033) | 0.114 (0.01-0.426) | 2.391 (0.201-8.811) | 0.08 (0.007-0.292) | -1.32 (-1.64 to -1) |
| Republic of Cabo Verde | 0.267 (0.125-0.505) | 0.385 (0.181-0.727) | 0.165 (0.072-0.325) | 0.112 (0.049-0.222) | -5.06 (-6.18 to -3.94) |
| Republic of Cameroon | 1.927 (0.366-5.869) | 0.086 (0.016-0.263) | 5.466 (1.617-13.605) | 0.073 (0.022-0.182) | 0.68 (0.28 to 1.1) |
| Republic of Chad | 14.576 (3.579-32.106) | 1.187 (0.293-2.617) | 34.978 (12.459-72.463) | 0.991 (0.352-2.049) | -0.4 (-0.9 to 0.11) |
| Republic of Chile | 0.028 (0.01-0.059) | 0.001 (0-0.002) | 0.077 (0.036-0.143) | 0.002 (0.001-0.003) | -0.34 (-2.53 to 1.89) |
| Republic of Colombia | 0.088 (0.036-0.179) | 0.001 (0-0.002) | 0.398 (0.17-0.785) | 0.003 (0.001-0.006) | 3.3 (2.32 to 4.28) |
| Republic of Costa Rica | 0.013 (0.004-0.03) | 0.002 (0.001-0.004) | 0.046 (0.02-0.088) | 0.003 (0.001-0.007) | 2.79 (2 to 3.6) |
| Republic of Croatia | 0.064 (0.038-0.099) | 0.005 (0.003-0.008) | 0.021 (0.011-0.034) | 0.002 (0.001-0.004) | -3.04 (-3.73 to -2.34) |
| Republic of Cuba | 0.859 (0.31-1.778) | 0.029 (0.01-0.061) | 0.322 (0.167-0.554) | 0.012 (0.006-0.02) | -3.51 (-3.87 to -3.16) |
| Republic of Cyprus | 0.009 (0.004-0.018) | 0.005 (0.002-0.009) | 0.004 (0.002-0.007) | 0.001 (0-0.002) | -5.75 (-6.54 to -4.95) |
| Republic of C么te d'Ivoire | 17.365 (5.672-39.315) | 0.685 (0.227-1.553) | 9.067 (3.573-19.774) | 0.141 (0.056-0.308) | -3.83 (-4.35 to -3.3) |
| Republic of Djibouti | 0.202 (0.047-0.538) | 0.238 (0.057-0.628) | 0.618 (0.183-1.438) | 0.197 (0.058-0.458) | -0.66 (-1.16 to -0.15) |
| Republic of Ecuador | 0.099 (0.028-0.247) | 0.004 (0.001-0.011) | 0.151 (0.049-0.345) | 0.003 (0.001-0.007) | -0.46 (-1.97 to 1.07) |
| Republic of El Salvador | 0.072 (0.014-0.202) | 0.006 (0.001-0.017) | 0.15 (0.048-0.347) | 0.008 (0.003-0.02) | 1.71 (1.2 to 2.22) |
| Republic of Equatorial Guinea | 0.027 (0.002-0.102) | 0.03 (0.002-0.112) | 0.085 (0.023-0.215) | 0.025 (0.007-0.062) | -0.71 (-1.05 to -0.37) |
| Republic of Estonia | 0.247 (0.111-0.444) | 0.071 (0.032-0.127) | 0.031 (0.015-0.052) | 0.014 (0.007-0.023) | -6.1 (-6.87 to -5.32) |
| Republic of Fiji | 0.188 (0.053-0.455) | 0.102 (0.028-0.248) | 0.056 (0.016-0.134) | 0.024 (0.007-0.059) | -4.33 (-4.57 to -4.08) |
| Republic of Finland | 0.085 (0.045-0.143) | 0.007 (0.003-0.011) | 0.02 (0.01-0.036) | 0.002 (0.001-0.003) | -5.41 (-5.92 to -4.89) |
| Republic of Ghana | 89.462 (30.618-194.794) | 2.618 (0.898-5.659) | 12.519 (3.328-32.387) | 0.138 (0.036-0.359) | -9.37 (-9.96 to -8.77) |
| Republic of Guatemala | 0.034 (0.008-0.089) | 0.002 (0.001-0.005) | 0.125 (0.045-0.268) | 0.003 (0.001-0.007) | 2.7 (1.94 to 3.47) |
| Republic of Guinea | 22.16 (5.772-48.949) | 1.708 (0.442-3.77) | 23.673 (9.9-46.561) | 0.746 (0.31-1.466) | -2.51 (-2.94 to -2.09) |
| Republic of Guinea-Bissau | 5.831 (1.514-13.126) | 2.673 (0.69-6.045) | 4.385 (1.763-8.835) | 0.884 (0.356-1.79) | -2.96 (-3.55 to -2.37) |
| Republic of Guyana | 0.013 (0.005-0.026) | 0.007 (0.003-0.014) | 0.102 (0.053-0.175) | 0.051 (0.026-0.087) | 2.68 (0.42 to 4.99) |
| Republic of Haiti | 1.312 (0.197-4.365) | 0.092 (0.014-0.302) | 3.64 (0.902-9.716) | 0.105 (0.026-0.278) | 0.28 (-0.05 to 0.62) |
| Republic of Honduras | 0.086 (0.015-0.274) | 0.01 (0.002-0.031) | 0.396 (0.1-1.033) | 0.015 (0.004-0.04) | 1.17 (0.8 to 1.56) |
| Republic of Iceland | 0.003 (0.002-0.005) | 0.005 (0.003-0.007) | 0 (0-0.001) | 0 (0-0.001) | -7.64 (-9.25 to -6.01) |
| Republic of India | 763.251 (323.457-1754.029) | 0.372 (0.156-0.86) | 1795.502 (962.267-3208.624) | 0.472 (0.253-0.844) | 0.76 (0.37 to 1.14) |
| Republic of Indonesia | 145.014 (29.01-415.263) | 0.31 (0.062-0.887) | 168.162 (51-415.396) | 0.217 (0.066-0.537) | -1.64 (-1.96 to -1.31) |
| Republic of Iraq | 7.786 (2.219-18.126) | 0.237 (0.068-0.55) | 10.079 (4.141-19.852) | 0.103 (0.042-0.203) | -2.78 (-2.98 to -2.57) |
| Republic of Italy | 0.218 (0.169-0.289) | 0.002 (0.001-0.002) | 0.545 (0.432-0.674) | 0.004 (0.003-0.005) | 1.13 (-0.99 to 3.29) |
| Republic of Kazakhstan | 29.081 (18.989-41.877) | 0.687 (0.445-0.994) | 2.784 (1.668-4.196) | 0.058 (0.035-0.088) | -8.46 (-8.97 to -7.95) |
| Republic of Kenya | 22.193 (6.394-45.529) | 0.474 (0.134-0.986) | 41.667 (19.987-79.044) | 0.343 (0.164-0.66) | -0.93 (-1.32 to -0.53) |
| Republic of Kiribati | 0.125 (0.046-0.256) | 0.714 (0.262-1.469) | 0.078 (0.027-0.172) | 0.248 (0.084-0.552) | -3.68 (-4.12 to -3.24) |
| Republic of Korea | 8.255 (3.177-17.376) | 0.071 (0.028-0.148) | 0.408 (0.171-0.823) | 0.003 (0.001-0.006) | -11.56 (-12.33 to -10.78) |
| Republic of Latvia | 2.75 (1.599-4.181) | 0.446 (0.261-0.676) | 0.153 (0.078-0.264) | 0.044 (0.023-0.076) | -7.47 (-8.06 to -6.89) |
| Republic of Liberia | 3.878 (1.253-8.46) | 0.726 (0.235-1.581) | 6.833 (2.8-13.511) | 0.502 (0.206-0.993) | -2.02 (-2.71 to -1.33) |
| Republic of Lithuania | 0.963 (0.501-1.628) | 0.108 (0.057-0.182) | 0.085 (0.047-0.139) | 0.015 (0.008-0.025) | -6.1 (-7 to -5.19) |
| Republic of Madagascar | 11.821 (3.264-28.045) | 0.479 (0.134-1.122) | 12.363 (2.877-31.021) | 0.178 (0.042-0.445) | -3.42 (-3.59 to -3.26) |
| Republic of Malawi | 35.402 (5.795-84.809) | 1.685 (0.283-4.035) | 36.035 (9.41-80.877) | 0.765 (0.202-1.709) | -3.53 (-4.1 to -2.95) |
| Republic of Maldives | 0.129 (0.026-0.344) | 0.34 (0.066-0.895) | 0.037 (0.018-0.068) | 0.031 (0.015-0.058) | -8.08 (-8.26 to -7.91) |
| Republic of Mali | 1.067 (0.148-3.766) | 0.059 (0.008-0.207) | 2.593 (0.441-9.116) | 0.051 (0.009-0.179) | -0.49 (-0.82 to -0.17) |
| Republic of Malta | 0.005 (0.002-0.008) | 0.005 (0.002-0.008) | 0.002 (0.001-0.003) | 0.002 (0.001-0.003) | -5.2 (-6.22 to -4.16) |
| Republic of Mauritius | 0.005 (0.001-0.013) | 0.002 (0.001-0.005) | 0.004 (0.001-0.008) | 0.001 (0-0.002) | -3.33 (-4.95 to -1.69) |
| Republic of Moldova | 2.989 (1.718-4.696) | 0.277 (0.16-0.434) | 0.242 (0.128-0.415) | 0.031 (0.017-0.053) | -7.19 (-7.87 to -6.51) |
| Republic of Mozambique | 51.02 (5.81-133.062) | 1.721 (0.197-4.467) | 24.198 (5.558-59.705) | 0.34 (0.08-0.829) | -5.79 (-6.18 to -5.41) |
| Republic of Namibia | 0.568 (0.206-1.243) | 0.205 (0.077-0.446) | 0.493 (0.192-1.028) | 0.082 (0.032-0.17) | -2.98 (-3.43 to -2.53) |
| Republic of Nauru | 0.002 (0.001-0.004) | 0.077 (0.024-0.175) | 0.001 (0-0.002) | 0.03 (0.01-0.067) | -3.09 (-3.75 to -2.43) |
| Republic of Nicaragua | 0.035 (0.005-0.112) | 0.004 (0.001-0.014) | 0.086 (0.022-0.218) | 0.005 (0.001-0.012) | 1.46 (0.91 to 2.01) |
| Republic of Niue | 0 (0-0) | 0.02 (0.006-0.048) | 0 (0-0) | 0.008 (0.003-0.021) | -4.23 (-4.67 to -3.78) |
| Republic of Palau | 0.009 (0.004-0.018) | 0.235 (0.105-0.461) | 0.004 (0.002-0.008) | 0.11 (0.05-0.209) | -2.36 (-2.52 to -2.2) |
| Republic of Panama | 0.01 (0.003-0.025) | 0.002 (0.001-0.004) | 0.029 (0.011-0.06) | 0.003 (0.001-0.006) | 2.13 (1.5 to 2.77) |
| Republic of Paraguay | 0.235 (0.054-0.595) | 0.027 (0.006-0.068) | 0.283 (0.099-0.612) | 0.015 (0.005-0.033) | -2.02 (-2.45 to -1.59) |
| Republic of Peru | 0.556 (0.161-1.319) | 0.011 (0.003-0.026) | 0.566 (0.193-1.188) | 0.006 (0.002-0.012) | -2.98 (-3.46 to -2.5) |
| Republic of Poland | 0.055 (0.04-0.075) | 0.001 (0-0.001) | 0.244 (0.18-0.317) | 0.003 (0.002-0.003) | 2.21 (-0.72 to 5.24) |
| Republic of Rwanda | 18.434 (4.488-50.784) | 1.224 (0.299-3.374) | 9.821 (2.323-24.283) | 0.292 (0.07-0.724) | -5.58 (-6.03 to -5.12) |
| Republic of San Marino | 0.004 (0.001-0.007) | 0.058 (0.02-0.118) | 0.002 (0.001-0.005) | 0.029 (0.01-0.066) | -1.51 (-1.89 to -1.13) |
| Republic of Senegal | 4.535 (1.527-9.998) | 0.282 (0.095-0.622) | 4.804 (1.7-10.413) | 0.131 (0.046-0.283) | -2.55 (-2.82 to -2.27) |
| Republic of Serbia | 1.152 (0.497-2.273) | 0.05 (0.021-0.098) | 0.321 (0.146-0.625) | 0.015 (0.007-0.029) | -4.4 (-4.9 to -3.89) |
| Republic of Seychelles | 0.015 (0.002-0.048) | 0.096 (0.016-0.299) | 0.01 (0.002-0.031) | 0.04 (0.008-0.119) | -2.24 (-2.49 to -2) |
| Republic of Sierra Leone | 14.117 (3.42-34.078) | 1.517 (0.365-3.652) | 9.675 (3.843-19.494) | 0.449 (0.179-0.911) | -3.82 (-4.77 to -2.87) |
| Republic of Singapore | 0.009 (0.004-0.018) | 0.001 (0-0.002) | 0.012 (0.005-0.021) | 0.001 (0-0.001) | -6.23 (-9.86 to -2.47) |
| Republic of Slovenia | 0.085 (0.046-0.141) | 0.017 (0.009-0.028) | 0.021 (0.011-0.034) | 0.005 (0.003-0.008) | -4.25 (-5.12 to -3.37) |
| Republic of South Africa | 17.581 (11.164-26.354) | 0.203 (0.13-0.303) | 10.677 (6.833-15.926) | 0.068 (0.044-0.102) | -2.72 (-3.57 to -1.87) |
| Republic of South Sudan | 5.183 (0.943-12.967) | 0.451 (0.084-1.113) | 10.101 (2.177-25.836) | 0.447 (0.096-1.145) | 0.5 (0.17 to 0.84) |
| Republic of Sudan | 89.532 (10.22-212.439) | 2.317 (0.268-5.404) | 45.652 (10.699-106.849) | 0.461 (0.11-1.06) | -5.4 (-5.54 to -5.26) |
| Republic of Suriname | 0.048 (0.02-0.092) | 0.052 (0.022-0.099) | 0.058 (0.027-0.11) | 0.04 (0.019-0.075) | -1.37 (-1.64 to -1.1) |
| Republic of Tajikistan | 29.823 (18.847-43.386) | 2.273 (1.43-3.318) | 10.624 (5.247-19.853) | 0.404 (0.2-0.755) | -5.95 (-6.35 to -5.55) |
| Republic of Trinidad and Tobago | 0.135 (0.054-0.266) | 0.045 (0.019-0.089) | 0.186 (0.085-0.354) | 0.051 (0.023-0.097) | 0.13 (-0.36 to 0.62) |
| Republic of Tunisia | 9.294 (3.288-19.092) | 0.54 (0.194-1.098) | 3.995 (1.782-7.725) | 0.117 (0.052-0.227) | -5.01 (-5.2 to -4.82) |
| Republic of Turkey | 18.677 (7.121-37.789) | 0.147 (0.057-0.297) | 5.237 (2.554-9.7) | 0.022 (0.011-0.042) | -6.28 (-6.55 to -6) |
| Republic of Uganda | 24.072 (4.384-62.843) | 0.701 (0.131-1.807) | 24.207 (6.88-56.829) | 0.245 (0.07-0.573) | -4.79 (-5.53 to -4.05) |
| Republic of Uzbekistan | 111.19 (77.062-151.834) | 2.088 (1.44-2.862) | 14.509 (9.609-20.688) | 0.16 (0.106-0.228) | -8.04 (-8.34 to -7.73) |
| Republic of Vanuatu | 0.021 (0.004-0.062) | 0.066 (0.014-0.197) | 0.014 (0.003-0.044) | 0.019 (0.003-0.059) | -4.75 (-5.18 to -4.31) |
| Republic of Yemen | 70.357 (7.235-190.735) | 3.517 (0.384-9.166) | 41.589 (12.821-94.909) | 0.601 (0.191-1.353) | -6.48 (-6.81 to -6.16) |
| Republic of Zambia | 44.066 (6.455-106.832) | 2.585 (0.382-6.272) | 19.738 (6.514-42.269) | 0.426 (0.141-0.904) | -6.36 (-6.87 to -5.86) |
| Republic of Zimbabwe | 6.699 (1.322-18.085) | 0.361 (0.072-0.968) | 4.778 (0.894-14.613) | 0.135 (0.026-0.415) | -1.45 (-2.81 to -0.07) |
| Republic of the Congo | 15.968 (1.589-45.992) | 3.243 (0.32-9.383) | 11.202 (2.46-29.673) | 0.809 (0.177-2.146) | -4.32 (-4.88 to -3.75) |
| Republic of the Gambia | 0.163 (0.034-0.453) | 0.079 (0.016-0.218) | 0.192 (0.032-0.598) | 0.034 (0.006-0.103) | -2.5 (-3.09 to -1.91) |
| Republic of the Marshall Islands | 0.007 (0.002-0.018) | 0.078 (0.019-0.205) | 0.005 (0.002-0.012) | 0.037 (0.012-0.082) | -2.73 (-3.33 to -2.13) |
| Republic of the Niger | 22.049 (4.861-49.074) | 1.382 (0.304-3.065) | 20.452 (6.611-46.518) | 0.421 (0.136-0.961) | -3.28 (-3.68 to -2.89) |
| Republic of the Philippines | 77.21 (38.023-118.196) | 0.529 (0.26-0.806) | 10.766 (7.894-14.38) | 0.038 (0.028-0.051) | -9.08 (-9.75 to -8.41) |
| Republic of the Union of Myanmar | 0.89 (0.045-4.242) | 0.009 (0-0.043) | 1.493 (0.139-6.294) | 0.01 (0.001-0.042) | -0.04 (-0.41 to 0.33) |
| Romania | 1.484 (0.746-2.734) | 0.027 (0.013-0.049) | 0.43 (0.238-0.682) | 0.01 (0.006-0.016) | -3.88 (-4.31 to -3.45) |
| Russian Federation | 25.398 (21.455-29.28) | 0.075 (0.064-0.086) | 4.602 (3.642-5.388) | 0.015 (0.011-0.017) | -6.52 (-7.5 to -5.52) |
| Saint Kitts and Nevis | 0.009 (0.003-0.021) | 0.101 (0.039-0.224) | 0.003 (0.002-0.006) | 0.022 (0.01-0.039) | -6.1 (-6.47 to -5.72) |
| Saint Lucia | 0.029 (0.014-0.054) | 0.096 (0.046-0.179) | 0.014 (0.008-0.025) | 0.03 (0.016-0.052) | -4.37 (-5.09 to -3.64) |
| Saint Vincent and the Grenadines | 0.001 (0.001-0.002) | 0.005 (0.002-0.01) | 0.007 (0.004-0.012) | 0.024 (0.013-0.041) | 2.53 (-0.01 to 5.14) |
| Slovak Republic | 0.229 (0.104-0.425) | 0.017 (0.008-0.032) | 0.105 (0.046-0.209) | 0.008 (0.004-0.016) | -2.23 (-2.78 to -1.67) |
| Socialist Republic of Viet Nam | 65.91 (17.556-153.245) | 0.437 (0.117-1.008) | 11.52 (3.548-26.427) | 0.042 (0.013-0.098) | -7.73 (-7.87 to -7.59) |
| Solomon Islands | 0.075 (0.013-0.216) | 0.115 (0.02-0.341) | 0.032 (0.005-0.108) | 0.02 (0.003-0.066) | -5.97 (-6.16 to -5.78) |
| State of Eritrea | 4.105 (0.856-10.799) | 0.593 (0.124-1.563) | 4.389 (1.031-10.869) | 0.284 (0.067-0.699) | -2.15 (-2.49 to -1.81) |
| State of Israel | 0.185 (0.098-0.317) | 0.015 (0.008-0.026) | 0.067 (0.034-0.115) | 0.003 (0.001-0.005) | -5.79 (-6.24 to -5.33) |
| State of Kuwait | 0.041 (0.018-0.076) | 0.012 (0.006-0.023) | 0.027 (0.013-0.047) | 0.002 (0.001-0.003) | -7.85 (-8.7 to -7) |
| State of Libya | 2.238 (0.459-5.603) | 0.32 (0.069-0.782) | 3.958 (1.58-9.1) | 0.185 (0.074-0.426) | -1.65 (-1.94 to -1.36) |
| State of Qatar | 0.121 (0.05-0.242) | 0.18 (0.074-0.363) | 0.159 (0.076-0.298) | 0.029 (0.014-0.055) | -6.06 (-6.52 to -5.6) |
| Sultanate of Oman | 0.113 (0.017-0.431) | 0.042 (0.006-0.158) | 0.157 (0.034-0.436) | 0.016 (0.003-0.045) | -2.53 (-2.75 to -2.31) |
| Swiss Confederation | 0.201 (0.102-0.36) | 0.012 (0.006-0.021) | 0.03 (0.011-0.063) | 0.001 (0.001-0.003) | -8.46 (-9.43 to -7.47) |
| Syrian Arab Republic | 1.169 (0.178-3.645) | 0.053 (0.008-0.169) | 0.347 (0.079-0.947) | 0.008 (0.002-0.023) | -6.75 (-7.6 to -5.89) |
| Taiwan (Province of China) | 0.037 (0.014-0.092) | 0.001 (0-0.002) | 0.041 (0.019-0.075) | 0.001 (0-0.001) | 1.24 (0.44 to 2.04) |
| Togolese Republic | 0.638 (0.118-1.952) | 0.079 (0.015-0.239) | 1.384 (0.332-3.776) | 0.065 (0.016-0.179) | 0.38 (-0.2 to 0.96) |
| Tokelau | 0 (0-0) | 0.061 (0.018-0.14) | 0 (0-0) | 0.053 (0.023-0.102) | -1.79 (-2.22 to -1.36) |
| Turkmenistan | 19.964 (11.715-30.821) | 2.059 (1.195-3.213) | 5.61 (2.872-10.106) | 0.442 (0.226-0.796) | -4.7 (-5.11 to -4.3) |
| Tuvalu | 0.002 (0-0.004) | 0.068 (0.017-0.187) | 0.001 (0-0.002) | 0.029 (0.01-0.062) | -3.19 (-3.59 to -2.79) |
| Ukraine | 15.484 (10.059-22.942) | 0.128 (0.084-0.189) | 3.462 (1.468-6.642) | 0.036 (0.015-0.068) | -3.82 (-5.12 to -2.5) |
| Union of the Comoros | 0.384 (0.089-0.986) | 0.391 (0.094-0.992) | 0.513 (0.155-1.207) | 0.266 (0.081-0.624) | -1.87 (-2.39 to -1.34) |
| United Arab Emirates | 0.323 (0.122-0.656) | 0.124 (0.047-0.253) | 0.892 (0.433-1.567) | 0.046 (0.022-0.081) | -2.86 (-3.13 to -2.59) |
| United Kingdom of Great Britain and Northern Ireland | 0.018 (0.015-0.022) | 0 (0-0) | 0.157 (0.133-0.181) | 0.001 (0.001-0.001) | 8.22 (4.45 to 12.12) |
| United Mexican States | 0.599 (0.456-0.768) | 0.003 (0.002-0.004) | 4.265 (3.234-5.483) | 0.012 (0.009-0.015) | 5.77 (4.42 to 7.14) |
| United Republic of Tanzania | 37.169 (7.655-92.758) | 0.665 (0.138-1.641) | 28.53 (7.564-70.337) | 0.2 (0.053-0.49) | -4.11 (-4.41 to -3.8) |
| United States Virgin Islands | 0.002 (0.001-0.004) | 0.007 (0.003-0.016) | 0.002 (0.001-0.003) | 0.009 (0.004-0.018) | 1.36 (0.08 to 2.66) |
| United States of America | 5.29 (4.277-6.551) | 0.008 (0.006-0.01) | 2.596 (1.958-3.316) | 0.003 (0.002-0.004) | -4.06 (-4.78 to -3.33) |
| (SDI: Socio-Demographic Index, ASR: Age-standardized rate, EAPC: Estimated Annual Percentage Change, UI: uncertainty intervals, CI: confidence intervals, AHB: Acute hepatitis B, ASMR: Age-standardized rate of Mortality) | | | | | |

| **Table S4. DALYs and ASDR of AHB in 1990 and 2021 and its trends (National level).** | | | | | |
| --- | --- | --- | --- | --- | --- |
| **location** | **Number (95% UI) 1990** | **ASR (95% UI) 1990** | **Number (95% UI) 2021** | **ASR (95% UI) 2021** | **EAPC (95%CI) 1990–2021** |
| American Samoa | 0.561 (0.245-1.067) | 5.073 (2.219-9.652) | 0.43 (0.196-0.815) | 3.643 (1.661-6.928) | -1.14 (-1.35 to -0.94) |
| Antigua and Barbuda | 0.202 (0.102-0.358) | 1.27 (0.642-2.235) | 0.165 (0.093-0.273) | 0.654 (0.368-1.081) | -2.43 (-2.93 to -1.92) |
| Arab Republic of Egypt | 9929.59 (2660.059-23742.764) | 82.657 (22.505-196.233) | 4289.393 (1810.243-9433.291) | 17.388 (7.345-38.284) | -4.88 (-5.08 to -4.68) |
| Argentine Republic | 57.659 (26.329-121.536) | 0.722 (0.33-1.518) | 41.3 (21.55-74.616) | 0.336 (0.175-0.609) | -2.85 (-3.18 to -2.52) |
| Australia | 57.387 (19.825-119.845) | 1.262 (0.441-2.626) | 68.847 (25.02-141.583) | 1.038 (0.384-2.122) | -0.82 (-0.93 to -0.71) |
| Barbados | 1.119 (0.433-2.295) | 1.632 (0.634-3.324) | 1.411 (0.634-2.644) | 1.919 (0.852-3.617) | 0.69 (0.31 to 1.07) |
| Belize | 0.897 (0.368-1.831) | 2.39 (0.984-4.837) | 1.484 (0.827-2.493) | 1.275 (0.713-2.135) | -2.26 (-2.67 to -1.86) |
| Bermuda | 0.065 (0.029-0.131) | 0.36 (0.164-0.722) | 0.039 (0.017-0.082) | 0.263 (0.114-0.544) | -1.1 (-1.2 to -1) |
| Bolivarian Republic of Venezuela | 19.486 (8.16-39.894) | 0.42 (0.176-0.86) | 41.189 (17.972-79.882) | 0.579 (0.251-1.126) | 1.39 (1.19 to 1.59) |
| Bosnia and Herzegovina | 36.293 (18.462-62.605) | 3.117 (1.586-5.378) | 12.098 (6.369-21.46) | 1.607 (0.85-2.835) | -2.32 (-2.54 to -2.1) |
| Brunei Darussalam | 18.725 (8.655-34.918) | 31.01 (14.36-57.694) | 4.032 (2.2-6.794) | 3.06 (1.668-5.161) | -7.98 (-8.39 to -7.57) |
| Burkina Faso | 1275.964 (363.279-2650.624) | 61.81 (17.799-128.136) | 1852.884 (718.622-3687.989) | 33.77 (13.443-66.175) | -2.27 (-2.96 to -1.57) |
| Canada | 85.219 (35.627-174.14) | 1.115 (0.476-2.256) | 77.5 (28.8-165.506) | 0.873 (0.333-1.851) | -1.1 (-1.21 to -0.99) |
| Central African Republic | 688.991 (100.927-1887.104) | 112.937 (16.623-306.841) | 681.692 (176.951-1595.199) | 50.436 (13.131-117.968) | -2.5 (-2.73 to -2.26) |
| Commonwealth of Dominica | 0.412 (0.171-0.862) | 2.611 (1.092-5.431) | 0.312 (0.16-0.55) | 1.894 (0.972-3.342) | -1.15 (-1.26 to -1.04) |
| Commonwealth of the Bahamas | 1.967 (0.852-3.936) | 2.773 (1.22-5.494) | 1.942 (0.99-3.312) | 1.781 (0.902-3.047) | -1.68 (-2.38 to -0.98) |
| Cook Islands | 0.083 (0.023-0.192) | 1.938 (0.538-4.494) | 0.043 (0.013-0.096) | 0.973 (0.307-2.174) | -2 (-2.26 to -1.74) |
| Czech Republic | 34.099 (19.51-56.857) | 1.305 (0.745-2.176) | 23.044 (13.276-39.107) | 0.964 (0.553-1.618) | -1.38 (-2.16 to -0.58) |
| Democratic People's Republic of Korea | 1514.123 (558.303-3121.535) | 27.73 (10.288-56.997) | 532.81 (237.392-1022.976) | 7.713 (3.428-14.851) | -4.25 (-4.64 to -3.85) |
| Democratic Republic of Sao Tome and Principe | 4.984 (2.039-9.784) | 20.954 (8.86-40.498) | 4.986 (2.391-9.324) | 9.414 (4.545-17.481) | -3.07 (-3.69 to -2.44) |
| Democratic Republic of Timor-Leste | 5.162 (1.418-12.214) | 2.932 (0.789-6.992) | 7.904 (2.415-18.063) | 2.492 (0.739-5.785) | -0.64 (-0.78 to -0.51) |
| Democratic Republic of the Congo | 909.825 (321.156-1849.201) | 11.296 (4.071-22.844) | 2074.67 (812.32-4003.931) | 10.197 (4.034-19.654) | -0.31 (-0.53 to -0.08) |
| Democratic Socialist Republic of Sri Lanka | 169.502 (60.533-383.238) | 3.805 (1.371-8.542) | 97.975 (42.475-186.316) | 1.685 (0.73-3.212) | -3.3 (-3.63 to -2.97) |
| Dominican Republic | 21.538 (10.498-39.258) | 1.213 (0.582-2.224) | 53.287 (27.302-95.773) | 1.834 (0.941-3.293) | 0.95 (0.33 to 1.58) |
| Eastern Republic of Uruguay | 2.802 (1.4-5.101) | 0.373 (0.186-0.679) | 2.114 (1.1-3.904) | 0.24 (0.124-0.447) | -1.87 (-2.07 to -1.66) |
| Federal Democratic Republic of Ethiopia | 5281.14 (1280.896-13965.776) | 48.546 (11.935-126.865) | 6206.048 (1714.211-13864.931) | 22.469 (6.487-49.988) | -3.41 (-3.81 to -3.01) |
| Federal Democratic Republic of Nepal | 963.646 (111.753-4056.216) | 20.941 (2.545-87.979) | 2628.722 (495.579-8228.003) | 28.835 (5.475-90.13) | 1.38 (1.21 to 1.56) |
| Federal Republic of Germany | 221.797 (127.651-355.192) | 1.141 (0.653-1.834) | 48.642 (23.38-91.558) | 0.255 (0.123-0.479) | -5.04 (-5.3 to -4.78) |
| Federal Republic of Nigeria | 35976.424 (9339.898-80256.761) | 188.863 (48.927-423.133) | 17552.945 (8280.61-32343.118) | 31.516 (15.026-57.767) | -6.47 (-6.99 to -5.95) |
| Federal Republic of Somalia | 2893.132 (311.149-8076.989) | 179.417 (19.463-503.917) | 7711.463 (1172.197-18441.075) | 162.094 (24.579-387.183) | -0.12 (-0.32 to 0.07) |
| Federated States of Micronesia | 1.102 (0.394-2.303) | 5.115 (1.818-10.736) | 0.641 (0.229-1.36) | 2.65 (0.935-5.636) | -2.03 (-2.2 to -1.85) |
| Federative Republic of Brazil | 678.037 (428.198-1098.359) | 1.843 (1.166-2.986) | 795.122 (558.308-1191.629) | 1.289 (0.908-1.922) | -1.66 (-2.28 to -1.04) |
| French Republic | 91.185 (52.531-152.368) | 0.625 (0.362-1.039) | 59.007 (30.388-109.434) | 0.395 (0.204-0.729) | -2.42 (-2.93 to -1.91) |
| Gabonese Republic | 61.53 (20.514-128.862) | 29.37 (9.939-61.397) | 33.884 (13.759-71.053) | 7.213 (2.943-15.008) | -4.41 (-4.71 to -4.11) |
| Georgia | 48.583 (24.106-87.675) | 3.455 (1.716-6.24) | 14.418 (7.747-24.993) | 1.793 (0.996-3.038) | -1.43 (-2.51 to -0.33) |
| Grand Duchy of Luxembourg | 0.642 (0.31-1.265) | 0.634 (0.309-1.242) | 0.756 (0.318-1.568) | 0.421 (0.178-0.871) | -1.6 (-1.85 to -1.34) |
| Greenland | 0.498 (0.218-0.972) | 3.39 (1.481-6.586) | 0.16 (0.065-0.321) | 1.215 (0.493-2.431) | -4.08 (-4.5 to -3.66) |
| Grenada | 2.108 (0.938-4.472) | 11.074 (4.994-23.37) | 1.191 (0.665-1.981) | 4.653 (2.6-7.749) | -3.08 (-3.56 to -2.59) |
| Guam | 0.998 (0.289-2.178) | 2.955 (0.847-6.449) | 1.039 (0.433-2.004) | 2.813 (1.183-5.412) | 0.16 (-0.17 to 0.49) |
| Hashemite Kingdom of Jordan | 139.526 (45.801-322.045) | 19.613 (6.547-44.708) | 79.618 (37.393-150.907) | 2.754 (1.301-5.203) | -6.72 (-6.96 to -6.48) |
| Hellenic Republic | 61.036 (32.756-104.625) | 2.405 (1.298-4.109) | 36.658 (16.888-69.401) | 1.49 (0.696-2.799) | -2.26 (-2.81 to -1.71) |
| Hungary | 48.585 (27.873-77.959) | 1.901 (1.094-3.04) | 24.921 (14.708-40.733) | 1.123 (0.669-1.826) | -1.96 (-2.37 to -1.56) |
| Independent State of Papua New Guinea | 70.511 (23.686-164.22) | 7.709 (2.537-18.045) | 107.371 (33.047-241.506) | 4.249 (1.308-9.562) | -2.15 (-2.26 to -2.03) |
| Independent State of Samoa | 1.752 (0.72-3.462) | 5.309 (2.144-10.544) | 1.858 (0.814-3.542) | 4.092 (1.781-7.823) | -0.69 (-0.82 to -0.55) |
| Ireland | 3.75 (1.524-7.878) | 0.43 (0.174-0.903) | 4.853 (1.958-10.292) | 0.392 (0.16-0.827) | -0.49 (-0.56 to -0.41) |
| Islamic Republic of Afghanistan | 8174.866 (739.318-21395.412) | 392.322 (35.759-1015.419) | 9529.927 (2164.019-23039.777) | 149.61 (34.189-357.91) | -3.74 (-4.41 to -3.07) |
| Islamic Republic of Iran | 1905.913 (1125.413-2770.062) | 17.036 (10.29-24.586) | 1315.448 (995.016-1733.108) | 5.336 (4.039-7.019) | -3.99 (-4.13 to -3.85) |
| Islamic Republic of Mauritania | 1219.906 (233.032-3109.534) | 264.165 (50.634-679.297) | 390.406 (187.093-748.828) | 38.523 (18.568-73.631) | -5.93 (-6.3 to -5.56) |
| Islamic Republic of Pakistan | 10649.943 (2504.02-30395.803) | 49.956 (11.769-142.964) | 35975.937 (11749.522-76650.07) | 62.786 (20.519-133.951) | 1.03 (0.82 to 1.24) |
| Jamaica | 9.73 (4.067-19.667) | 1.766 (0.743-3.545) | 16.995 (8.045-31.236) | 2.159 (1.023-3.969) | 0.32 (-0.07 to 0.71) |
| Japan | 609.016 (327.34-1101.514) | 1.799 (0.96-3.262) | 407.357 (224.486-727.815) | 1.477 (0.81-2.653) | -2.48 (-3.76 to -1.19) |
| Kingdom of Bahrain | 13.88 (5.902-27.405) | 14.07 (5.943-27.704) | 12.098 (5.847-22.303) | 3.586 (1.724-6.639) | -4.57 (-5.08 to -4.07) |
| Kingdom of Belgium | 23.688 (13.702-37.907) | 0.969 (0.561-1.55) | 12.619 (6.96-22.323) | 0.478 (0.262-0.849) | -3.24 (-3.8 to -2.68) |
| Kingdom of Bhutan | 54.14 (8.815-171.132) | 38.012 (6.414-117.695) | 45.331 (13.502-102.928) | 21.925 (6.572-49.726) | -2.38 (-3.06 to -1.69) |
| Kingdom of Cambodia | 2047.854 (395.573-5897.514) | 84.572 (16.561-242.756) | 848.365 (293.395-1892.634) | 19.007 (6.583-42.432) | -5.42 (-6.18 to -4.66) |
| Kingdom of Denmark | 9.771 (5.21-17.187) | 0.73 (0.39-1.282) | 6.539 (2.923-13.075) | 0.5 (0.224-1.003) | -2.2 (-2.55 to -1.86) |
| Kingdom of Eswatini | 69.217 (25.42-143.053) | 40.677 (15.202-83.41) | 33.683 (13.234-69.651) | 11.867 (4.726-24.412) | -3.65 (-4.32 to -2.98) |
| Kingdom of Lesotho | 130.542 (44.355-276.251) | 37.352 (12.895-78.127) | 101.389 (39.646-203.843) | 22.373 (8.777-44.614) | -0.47 (-1.32 to 0.38) |
| Kingdom of Morocco | 664.806 (195.368-1559.248) | 11.951 (3.572-27.743) | 763.36 (330.913-1491.179) | 7.705 (3.335-15.066) | -1.56 (-1.76 to -1.37) |
| Kingdom of Norway | 4.658 (2.049-9.372) | 0.43 (0.189-0.867) | 4.534 (2.167-8.901) | 0.351 (0.168-0.688) | -0.55 (-0.87 to -0.23) |
| Kingdom of Saudi Arabia | 1152.491 (336.875-2609.671) | 40.918 (11.933-92.625) | 517.43 (267.464-889.273) | 4.786 (2.46-8.25) | -6.22 (-6.79 to -5.63) |
| Kingdom of Spain | 93.462 (35.79-195.423) | 0.977 (0.372-2.048) | 66.435 (19.899-144.438) | 0.522 (0.165-1.12) | -1.86 (-2.15 to -1.58) |
| Kingdom of Sweden | 6.977 (3.077-14.037) | 0.33 (0.147-0.66) | 7.628 (3.572-14.9) | 0.334 (0.16-0.646) | -0.09 (-0.35 to 0.17) |
| Kingdom of Thailand | 1439.867 (735.76-2638.104) | 9.479 (4.88-17.318) | 789.361 (433.632-1350.123) | 4.339 (2.356-7.498) | -2.28 (-3.15 to -1.4) |
| Kingdom of Tonga | 16.305 (5.982-34.277) | 79.456 (28.657-168.775) | 3.762 (1.326-8.557) | 15.489 (5.485-35.162) | -5.4 (-5.59 to -5.21) |
| Kingdom of the Netherlands | 17.12 (9.072-31.076) | 0.423 (0.225-0.765) | 12.215 (5.705-23.358) | 0.317 (0.148-0.607) | -1.75 (-2.12 to -1.39) |
| Kyrgyz Republic | 746.156 (460.387-1262.353) | 65.565 (40.19-111.35) | 115.242 (71.39-179.423) | 6.589 (4.078-10.268) | -6.56 (-7.03 to -6.09) |
| Lao People's Democratic Republic | 122.055 (26.159-364.624) | 13.397 (2.867-39.938) | 95.987 (35.697-215.187) | 4.925 (1.845-10.986) | -3.83 (-4.46 to -3.19) |
| Lebanese Republic | 35.67 (14.65-72.371) | 5.113 (2.108-10.325) | 32.911 (16.528-57.83) | 2.064 (1.043-3.614) | -3.04 (-3.2 to -2.89) |
| Malaysia | 759.841 (393.093-1320.767) | 18.103 (9.429-31.417) | 704.178 (385.943-1198.03) | 8.532 (4.689-14.488) | -2.39 (-2.52 to -2.27) |
| Mongolia | 409.873 (178.915-777.584) | 73.893 (32.689-138.495) | 88.362 (48.5-150.12) | 10.364 (5.67-17.616) | -6.74 (-7.07 to -6.4) |
| Montenegro | 0.995 (0.463-1.975) | 0.637 (0.296-1.262) | 0.686 (0.317-1.367) | 0.452 (0.21-0.896) | -0.93 (-1.18 to -0.68) |
| New Zealand | 17.702 (9.614-30.829) | 1.955 (1.065-3.399) | 8.313 (3.142-18.205) | 0.648 (0.248-1.412) | -3.29 (-3.57 to -3.01) |
| North Macedonia | 10.866 (5.429-19.33) | 2.131 (1.064-3.792) | 7.123 (3.618-12.91) | 1.296 (0.657-2.367) | -1.97 (-2.56 to -1.37) |
| Northern Mariana Islands | 0.913 (0.42-1.66) | 7.122 (3.323-12.871) | 0.655 (0.335-1.139) | 5.041 (2.548-8.842) | -0.97 (-1.12 to -0.83) |
| Palestine | 24.397 (9.606-49.805) | 6.425 (2.537-13.145) | 26.743 (13.913-46.653) | 2.341 (1.228-4.063) | -3.26 (-3.33 to -3.19) |
| People's Democratic Republic of Algeria | 1742.174 (699.189-3476.388) | 35.451 (14.429-69.808) | 1283.107 (637.242-2353.344) | 10.99 (5.437-20.221) | -3.69 (-3.83 to -3.56) |
| People's Republic of Bangladesh | 8306.619 (1370.407-29691.65) | 38.064 (6.34-135.058) | 12630.429 (2963.465-31489.899) | 27.827 (6.535-69.371) | -0.73 (-1.01 to -0.46) |
| People's Republic of China | 69036.859 (39087.463-101346.451) | 23.067 (13.115-33.713) | 12122.984 (7024.05-21499.76) | 3.356 (1.96-5.897) | -6.53 (-6.97 to -6.09) |
| Plurinational State of Bolivia | 15.874 (6.937-33.72) | 1.109 (0.493-2.33) | 32.553 (15.859-60.962) | 1.064 (0.52-1.986) | -0.11 (-0.17 to -0.06) |
| Portuguese Republic | 32.928 (20.889-51.062) | 1.305 (0.828-2.022) | 16.847 (9.212-29.393) | 0.663 (0.364-1.156) | -3.37 (-3.96 to -2.76) |
| Principality of Andorra | 1.665 (0.546-4.023) | 11.453 (3.721-27.806) | 2.293 (0.936-4.67) | 11.156 (4.494-22.799) | 0.24 (-0.28 to 0.76) |
| Principality of Monaco | 0.05 (0.026-0.089) | 0.68 (0.356-1.209) | 0.039 (0.019-0.075) | 0.49 (0.246-0.946) | -1.03 (-1.26 to -0.8) |
| Puerto Rico | 4.404 (2.013-8.445) | 0.462 (0.211-0.886) | 8.082 (4.849-12.838) | 1.029 (0.612-1.641) | 1.89 (0.82 to 2.98) |
| Republic of Albania | 21.431 (11.858-35.241) | 2.521 (1.387-4.162) | 7.898 (4.008-14.343) | 1.261 (0.638-2.294) | -2.12 (-2.76 to -1.48) |
| Republic of Angola | 3946.952 (402.638-11488.006) | 175.672 (17.961-508.91) | 2620.431 (629.482-6654.702) | 34.571 (8.444-87.008) | -5.09 (-5.5 to -4.67) |
| Republic of Armenia | 64.949 (42.194-93.593) | 7.092 (4.593-10.25) | 10.081 (4.379-19.916) | 1.243 (0.562-2.399) | -4.86 (-5.17 to -4.54) |
| Republic of Austria | 13.188 (6.791-25.032) | 0.658 (0.339-1.248) | 9.4 (3.877-19.908) | 0.431 (0.179-0.913) | -1.9 (-2.24 to -1.56) |
| Republic of Azerbaijan | 480.338 (272.773-778.073) | 23.012 (13.002-37.454) | 132.279 (62.436-243.963) | 4.784 (2.257-8.836) | -5.39 (-5.72 to -5.06) |
| Republic of Belarus | 301.886 (194.495-441.103) | 12.398 (7.975-18.138) | 56.703 (31.789-94.368) | 2.783 (1.519-4.741) | -4.7 (-5.52 to -3.87) |
| Republic of Benin | 398.869 (135.786-854.591) | 37.208 (12.731-78.907) | 409.323 (170.129-880.171) | 13.13 (5.574-27.823) | -3.7 (-4.14 to -3.27) |
| Republic of Botswana | 67.495 (26.924-141.036) | 24.519 (9.942-50.587) | 38.228 (18.555-69.641) | 5.655 (2.738-10.301) | -3.78 (-4.4 to -3.16) |
| Republic of Bulgaria | 57.846 (29.38-102.957) | 2.792 (1.443-4.906) | 38.048 (20.484-64.679) | 2.583 (1.403-4.375) | -0.86 (-1.51 to -0.19) |
| Republic of Burundi | 98.371 (21.335-320.894) | 8.126 (1.826-25.872) | 185.039 (41.899-582.75) | 6.052 (1.464-18.555) | -1.03 (-1.3 to -0.76) |
| Republic of Cabo Verde | 17.623 (8.894-31.747) | 24.32 (12.282-43.692) | 12.596 (6.229-22.068) | 8.436 (4.152-14.788) | -4.46 (-5.42 to -3.5) |
| Republic of Cameroon | 180.464 (61.748-426.998) | 8.013 (2.747-18.713) | 508.302 (210.313-1039.411) | 6.82 (2.823-13.814) | 0.3 (0.02 to 0.59) |
| Republic of Chad | 896.352 (251.283-1916.612) | 70.451 (19.979-150.428) | 2201.585 (849.163-4455.981) | 59.589 (23.098-120.108) | -0.36 (-0.83 to 0.12) |
| Republic of Chile | 14.863 (7.038-28.09) | 0.43 (0.205-0.811) | 22.151 (11.244-40.674) | 0.446 (0.226-0.82) | -0.36 (-0.96 to 0.23) |
| Republic of Colombia | 226.465 (53.552-533.187) | 2.736 (0.625-6.477) | 247.14 (65.588-566.718) | 1.865 (0.494-4.278) | -1.02 (-1.25 to -0.8) |
| Republic of Costa Rica | 5.174 (2.211-10.79) | 0.694 (0.297-1.449) | 8.408 (4.118-15.98) | 0.623 (0.306-1.18) | -0.11 (-0.36 to 0.14) |
| Republic of Croatia | 13.183 (6.046-25.362) | 1.07 (0.503-2.033) | 7.048 (3.165-13.954) | 0.706 (0.321-1.386) | -1.44 (-1.67 to -1.2) |
| Republic of Cuba | 56.661 (24.89-108.357) | 1.893 (0.817-3.636) | 21.347 (11.795-35.239) | 0.8 (0.439-1.334) | -3.17 (-3.46 to -2.89) |
| Republic of Cyprus | 2.149 (0.896-4.235) | 1.075 (0.45-2.112) | 2.32 (0.852-4.917) | 0.522 (0.197-1.098) | -2.57 (-2.85 to -2.29) |
| Republic of C么te d'Ivoire | 1126.309 (411.012-2436.992) | 42.615 (15.781-91.913) | 708.995 (335.425-1372.932) | 10.906 (5.176-21.046) | -3.28 (-3.72 to -2.85) |
| Republic of Djibouti | 13.497 (4.226-33.251) | 15.03 (4.76-36.606) | 40.428 (14.571-88.595) | 12.741 (4.595-27.94) | -0.56 (-1.02 to -0.09) |
| Republic of Ecuador | 19.394 (9.85-34.807) | 0.841 (0.429-1.503) | 33.128 (17.272-59.965) | 0.711 (0.371-1.289) | -0.36 (-0.97 to 0.25) |
| Republic of El Salvador | 11.538 (4.682-23.6) | 0.95 (0.383-1.935) | 15.524 (7.106-29.77) | 0.879 (0.402-1.686) | 0.12 (-0.16 to 0.41) |
| Republic of Equatorial Guinea | 3.218 (1.051-8.077) | 3.453 (1.142-8.537) | 9.202 (3.909-18.876) | 2.63 (1.118-5.338) | -0.94 (-1.11 to -0.76) |
| Republic of Estonia | 19.947 (9.787-34.762) | 5.668 (2.797-9.838) | 4.001 (1.774-7.689) | 1.528 (0.706-2.854) | -4.75 (-5.27 to -4.24) |
| Republic of Fiji | 15.407 (5.918-31.696) | 8.143 (3.102-16.781) | 6.117 (2.447-12.394) | 2.662 (1.066-5.393) | -3.42 (-3.58 to -3.25) |
| Republic of Finland | 11.701 (6.461-20.555) | 0.898 (0.499-1.566) | 7.086 (3.352-14.044) | 0.602 (0.29-1.182) | -1.76 (-1.9 to -1.61) |
| Republic of Ghana | 5492.075 (1946.014-11873.377) | 155.449 (55.467-333.047) | 1065.999 (398.862-2304.591) | 11.808 (4.424-25.365) | -8.27 (-8.73 to -7.82) |
| Republic of Guatemala | 21.397 (7.025-47.777) | 1.257 (0.398-2.827) | 44.519 (15.824-97.872) | 1.072 (0.373-2.375) | -0.31 (-0.45 to -0.17) |
| Republic of Guinea | 1345.957 (386.659-2918.324) | 100.932 (29.005-218.367) | 1531.731 (702.635-2933.44) | 46.794 (21.399-89.108) | -2.36 (-2.76 to -1.95) |
| Republic of Guinea-Bissau | 354.018 (98.182-783.393) | 156.301 (43.263-347.075) | 277.649 (120.772-537.002) | 54.262 (23.696-105.244) | -2.86 (-3.42 to -2.29) |
| Republic of Guyana | 1.928 (1.001-3.485) | 1.026 (0.531-1.86) | 6.653 (3.753-10.732) | 3.3 (1.864-5.313) | 1.5 (-0.04 to 3.07) |
| Republic of Haiti | 83.708 (19.347-258.365) | 5.699 (1.335-17.417) | 226.542 (66.781-573.574) | 6.441 (1.907-16.239) | 0.3 (0 to 0.6) |
| Republic of Honduras | 13.637 (4.957-30.242) | 1.435 (0.51-3.182) | 38.178 (14.679-80.604) | 1.456 (0.563-3.043) | 0.03 (-0.2 to 0.26) |
| Republic of Iceland | 0.422 (0.238-0.732) | 0.652 (0.369-1.129) | 0.307 (0.131-0.646) | 0.372 (0.159-0.781) | -2.41 (-2.89 to -1.92) |
| Republic of India | 49980.439 (22678.103-110418.782) | 23.865 (10.832-52.767) | 114243.129 (63060.446-200538.831) | 29.883 (16.483-52.458) | 0.72 (0.31 to 1.13) |
| Republic of Indonesia | 9856.193 (2683.639-26152.744) | 20.565 (5.726-54.331) | 10666.632 (4033.21-24320.61) | 13.918 (5.26-31.761) | -1.73 (-2.04 to -1.41) |
| Republic of Iraq | 454.62 (145.141-1027.459) | 13.033 (4.221-29.419) | 580.833 (270.694-1092.691) | 5.857 (2.739-11.002) | -2.66 (-2.86 to -2.46) |
| Republic of Italy | 216.906 (97.029-432.806) | 1.506 (0.678-3.001) | 111.672 (59.973-208.867) | 0.766 (0.432-1.388) | -2.48 (-3.08 to -1.89) |
| Republic of Kazakhstan | 1830.349 (1216.138-2608.93) | 42.909 (28.368-61.387) | 193.868 (122.096-284.338) | 4.07 (2.537-5.983) | -8.13 (-8.59 to -7.66) |
| Republic of Kenya | 1473.415 (517.676-2886.136) | 29.741 (10.548-58.556) | 2643.548 (1349.079-4772.576) | 20.988 (10.763-38.258) | -0.97 (-1.33 to -0.6) |
| Republic of Kiribati | 8.241 (3.619-15.814) | 45.625 (19.999-87.797) | 5.61 (2.404-11.122) | 17.805 (7.628-35.284) | -3.27 (-3.64 to -2.91) |
| Republic of Korea | 725.293 (330.283-1334.169) | 5.983 (2.745-10.998) | 153.398 (68.973-299.023) | 1.05 (0.48-2.04) | -5.88 (-6.12 to -5.64) |
| Republic of Latvia | 173.032 (103.175-260.197) | 28.5 (17.045-42.787) | 10.72 (5.917-17.82) | 3.128 (1.715-5.231) | -7.13 (-7.66 to -6.59) |
| Republic of Liberia | 255.258 (94.825-531.739) | 46.163 (17.344-95.531) | 463.365 (211.283-865.684) | 33.443 (15.358-62.263) | -1.81 (-2.44 to -1.19) |
| Republic of Lithuania | 67.77 (37.579-110.211) | 7.634 (4.251-12.4) | 8.001 (4.629-13.22) | 1.398 (0.805-2.306) | -5.28 (-5.99 to -4.56) |
| Republic of Madagascar | 766.943 (246.841-1736.573) | 29.562 (9.699-66.105) | 890.402 (287.299-2008.325) | 12.511 (4.162-27.935) | -2.95 (-3.07 to -2.82) |
| Republic of Malawi | 2158.936 (383.633-5113.726) | 97.454 (17.917-230.49) | 2249.275 (617.505-4973.195) | 45.44 (12.728-99.939) | -3.4 (-3.97 to -2.84) |
| Republic of Maldives | 7.815 (2.014-19.972) | 19.262 (4.905-48.88) | 3.128 (1.586-5.452) | 2.565 (1.298-4.479) | -6.76 (-6.89 to -6.63) |
| Republic of Mali | 114.53 (34.191-300.284) | 6.28 (1.877-16.31) | 278.988 (92.401-713.999) | 5.552 (1.841-13.97) | -0.44 (-0.61 to -0.27) |
| Republic of Malta | 0.626 (0.341-1.1) | 0.651 (0.357-1.134) | 0.378 (0.177-0.732) | 0.353 (0.167-0.683) | -2.75 (-3.27 to -2.23) |
| Republic of Mauritius | 4.177 (1.288-9.599) | 1.425 (0.441-3.261) | 3.407 (0.97-8.024) | 1.019 (0.292-2.403) | -1.11 (-1.39 to -0.82) |
| Republic of Moldova | 239.582 (144.245-371.928) | 21.993 (13.288-34.048) | 42.398 (17.982-83.835) | 4.515 (2.03-8.646) | -5.05 (-5.4 to -4.69) |
| Republic of Mozambique | 3096.576 (435.705-7880.08) | 100.727 (14.229-254.837) | 1661.149 (503.111-3786.109) | 22.655 (7.157-50.396) | -5.37 (-5.72 to -5.02) |
| Republic of Namibia | 40.475 (17.781-78.66) | 13.769 (6.12-26.518) | 42.065 (19.253-77.356) | 6.751 (3.09-12.407) | -2.29 (-2.6 to -1.97) |
| Republic of Nauru | 0.193 (0.086-0.357) | 8.283 (3.698-15.415) | 0.096 (0.04-0.181) | 3.604 (1.502-6.799) | -2.77 (-3.28 to -2.26) |
| Republic of Nicaragua | 4.154 (1.67-9.242) | 0.504 (0.205-1.098) | 8.276 (3.464-16.754) | 0.464 (0.195-0.937) | 0.37 (0.04 to 0.71) |
| Republic of Niue | 0.02 (0.008-0.04) | 4.076 (1.547-8.345) | 0.006 (0.002-0.013) | 1.44 (0.475-3.052) | -2.96 (-3.27 to -2.65) |
| Republic of Palau | 0.651 (0.328-1.18) | 16.326 (8.248-29.542) | 0.328 (0.16-0.585) | 8.329 (4.044-15.091) | -2.06 (-2.22 to -1.91) |
| Republic of Panama | 3.193 (1.366-6.504) | 0.551 (0.234-1.127) | 4.963 (2.319-9.315) | 0.467 (0.218-0.877) | -0.05 (-0.25 to 0.15) |
| Republic of Paraguay | 20.008 (7.602-41.476) | 2.243 (0.861-4.615) | 27.674 (13.565-50.344) | 1.478 (0.727-2.688) | -1.48 (-1.78 to -1.17) |
| Republic of Peru | 95.806 (41.31-180.63) | 1.908 (0.812-3.612) | 144.757 (60.725-292.25) | 1.479 (0.622-2.981) | -1.09 (-1.25 to -0.94) |
| Republic of Poland | 87.99 (40.214-168.739) | 0.888 (0.408-1.696) | 43.422 (24.784-75.53) | 0.431 (0.253-0.735) | -2.95 (-3.64 to -2.26) |
| Republic of Rwanda | 1128.437 (289.357-3053.981) | 71.053 (18.304-192.27) | 622.168 (172.672-1470.888) | 17.943 (5.06-42.462) | -5.31 (-5.74 to -4.88) |
| Republic of San Marino | 0.241 (0.101-0.461) | 3.861 (1.617-7.375) | 0.152 (0.065-0.31) | 2.045 (0.849-4.249) | -1.44 (-1.78 to -1.1) |
| Republic of Senegal | 332.788 (137.784-678.662) | 20.103 (8.373-40.946) | 406.112 (181.28-784.765) | 10.956 (4.939-21.064) | -2.08 (-2.29 to -1.87) |
| Republic of Serbia | 82.605 (41.144-150.224) | 3.578 (1.774-6.53) | 28.614 (15.356-49.223) | 1.358 (0.724-2.351) | -3.59 (-3.97 to -3.2) |
| Republic of Seychelles | 1.206 (0.353-3.109) | 7.187 (2.11-18.487) | 0.885 (0.293-2.034) | 3.433 (1.126-7.948) | -1.88 (-2.06 to -1.7) |
| Republic of Sierra Leone | 860.75 (228.348-2039.369) | 88.717 (23.525-209.272) | 641.988 (287.254-1233.483) | 28.8 (12.995-55.394) | -3.52 (-4.39 to -2.65) |
| Republic of Singapore | 21.439 (5.623-48.611) | 2.277 (0.602-5.151) | 23.876 (6.628-54.535) | 1.233 (0.361-2.795) | -2.47 (-3.05 to -1.88) |
| Republic of Slovenia | 9.34 (5.067-15.996) | 1.868 (1.021-3.178) | 4.205 (1.94-8.165) | 0.903 (0.42-1.736) | -2.43 (-2.87 to -1.99) |
| Republic of South Africa | 1098.89 (723.873-1603.829) | 12.294 (8.151-17.9) | 651.473 (435.988-940.115) | 4.128 (2.754-5.969) | -2.92 (-3.7 to -2.15) |
| Republic of South Sudan | 335.988 (81.569-804.227) | 27.559 (6.842-64.87) | 648.852 (176.535-1583.359) | 27.889 (7.695-67.864) | 0.52 (0.2 to 0.83) |
| Republic of Sudan | 4987.977 (674.309-11798.288) | 122.604 (16.555-284.974) | 2714.594 (776.757-6205.069) | 26.355 (7.59-59.341) | -5.14 (-5.27 to -5.01) |
| Republic of Suriname | 3.136 (1.503-5.721) | 3.341 (1.617-6.025) | 3.807 (1.998-6.69) | 2.609 (1.369-4.589) | -1.25 (-1.49 to -1) |
| Republic of Tajikistan | 1898.183 (1205.403-2750.221) | 140.244 (88.897-203.684) | 683.921 (354.483-1253.831) | 25.876 (13.464-47.368) | -5.83 (-6.23 to -5.43) |
| Republic of Trinidad and Tobago | 8.785 (4.111-16.453) | 2.9 (1.374-5.387) | 11.196 (5.579-20.216) | 3.133 (1.558-5.673) | 0.02 (-0.41 to 0.46) |
| Republic of Tunisia | 551.875 (215.883-1089.378) | 30.614 (12.065-59.947) | 252.968 (128.038-446.811) | 7.504 (3.767-13.297) | -4.53 (-4.7 to -4.37) |
| Republic of Turkey | 1264.193 (575.931-2329.589) | 9.607 (4.393-17.615) | 552.023 (280.337-954.563) | 2.41 (1.22-4.179) | -4.52 (-4.74 to -4.3) |
| Republic of Uganda | 1520.777 (325.84-3852.951) | 41.568 (9.162-104.122) | 1671.009 (592.038-3673.78) | 16.347 (5.928-35.414) | -4.36 (-5.06 to -3.66) |
| Republic of Uzbekistan | 7165.661 (5017.06-9727.018) | 131.382 (91.678-178.63) | 1072.624 (698.071-1554.996) | 11.821 (7.713-17.098) | -7.59 (-7.88 to -7.29) |
| Republic of Vanuatu | 2.742 (1.051-5.595) | 8.361 (3.2-17.086) | 3.015 (1.053-6.435) | 4.118 (1.432-8.795) | -2.51 (-2.69 to -2.32) |
| Republic of Yemen | 3673.411 (430.539-10051.863) | 175.67 (21.246-461.412) | 2380.928 (839.959-5243.615) | 33.048 (11.914-71.998) | -6.12 (-6.42 to -5.82) |
| Republic of Zambia | 2700.332 (425.873-6499.091) | 149.25 (23.883-359.216) | 1261.694 (465.368-2613.974) | 26.147 (9.8-53.427) | -6.15 (-6.63 to -5.67) |
| Republic of Zimbabwe | 550.171 (199.587-1204.259) | 27.456 (9.979-60.24) | 522.3 (196.583-1111.5) | 14.253 (5.369-30.487) | -0.99 (-1.88 to -0.1) |
| Republic of the Congo | 940.227 (109.203-2662.534) | 180.746 (20.825-514.068) | 674.261 (176.641-1718.227) | 47.894 (12.561-122.227) | -4.13 (-4.68 to -3.58) |
| Republic of the Gambia | 16.793 (6.133-35.658) | 7.961 (2.953-16.839) | 21.228 (6.786-50.848) | 3.925 (1.278-9.13) | -2.07 (-2.29 to -1.86) |
| Republic of the Marshall Islands | 0.709 (0.286-1.428) | 7.83 (3.142-15.85) | 0.646 (0.264-1.263) | 4.445 (1.813-8.691) | -2.01 (-2.39 to -1.62) |
| Republic of the Niger | 1384.952 (364.121-2998.192) | 83.175 (21.927-179.007) | 1440.544 (563.555-3020.837) | 28.48 (11.194-59.37) | -2.97 (-3.31 to -2.63) |
| Republic of the Philippines | 5419.484 (2937.718-8018.492) | 36.046 (19.588-53.11) | 1767.466 (1043.808-3055.29) | 6.272 (3.688-10.887) | -5.96 (-6.45 to -5.47) |
| Republic of the Union of Myanmar | 122.119 (36.675-333.952) | 1.234 (0.371-3.3) | 172.751 (55.413-446.866) | 1.148 (0.369-2.959) | -0.32 (-0.52 to -0.12) |
| Romania | 197.058 (97.836-351.481) | 3.507 (1.754-6.223) | 78.865 (35.526-152.273) | 1.713 (0.787-3.25) | -2.44 (-2.63 to -2.26) |
| Russian Federation | 2088.485 (1660.869-2700.209) | 6.073 (4.894-7.703) | 587.368 (375.725-952.274) | 1.718 (1.143-2.65) | -5.08 (-5.87 to -4.28) |
| Saint Kitts and Nevis | 0.571 (0.229-1.265) | 5.988 (2.519-12.878) | 0.247 (0.13-0.407) | 1.543 (0.803-2.559) | -5.36 (-5.66 to -5.06) |
| Saint Lucia | 1.819 (0.919-3.258) | 5.794 (2.949-10.336) | 0.977 (0.568-1.595) | 2.062 (1.193-3.379) | -3.84 (-4.46 to -3.21) |
| Saint Vincent and the Grenadines | 0.156 (0.081-0.284) | 0.637 (0.327-1.16) | 0.458 (0.264-0.734) | 1.633 (0.94-2.622) | 1.45 (-0.44 to 3.37) |
| Slovak Republic | 25.576 (12.965-45.109) | 1.918 (0.979-3.372) | 15.29 (6.758-30.773) | 1.127 (0.507-2.244) | -1.55 (-1.85 to -1.24) |
| Socialist Republic of Viet Nam | 4402.989 (1481.185-9536.963) | 27.808 (9.398-59.685) | 1504.673 (628.323-2881.66) | 5.525 (2.311-10.59) | -5.35 (-5.54 to -5.15) |
| Solomon Islands | 7.963 (2.993-16.552) | 11.773 (4.384-24.586) | 6.802 (2.296-14.478) | 4.248 (1.428-9.037) | -3.24 (-3.36 to -3.13) |
| State of Eritrea | 252.986 (65.394-628.769) | 34.879 (9.103-86.873) | 288.069 (86.71-662.81) | 18.132 (5.537-41.428) | -1.88 (-2.2 to -1.57) |
| State of Israel | 14.967 (8.922-23.821) | 1.227 (0.732-1.952) | 8.945 (4.915-15.744) | 0.393 (0.216-0.692) | -4.04 (-4.33 to -3.75) |
| State of Kuwait | 4.657 (2.6-7.843) | 1.251 (0.708-2.088) | 7.554 (3.837-13.667) | 0.419 (0.214-0.753) | -3.91 (-4.3 to -3.51) |
| State of Libya | 134.689 (36.094-323.9) | 17.967 (4.953-42.253) | 223.728 (98.916-489.026) | 10.602 (4.677-23.205) | -1.56 (-1.84 to -1.28) |
| State of Qatar | 8.234 (4.005-14.914) | 11.482 (5.532-21.091) | 16.855 (8.16-30.231) | 2.851 (1.391-5.1) | -4.66 (-5 to -4.32) |
| Sultanate of Oman | 13.121 (4.335-32.244) | 4.369 (1.417-10.828) | 18.775 (7.762-38.438) | 1.816 (0.749-3.718) | -2.27 (-2.48 to -2.07) |
| Swiss Confederation | 20.77 (11.577-34.558) | 1.189 (0.665-1.971) | 10.975 (5.135-20.904) | 0.523 (0.248-0.988) | -4.02 (-4.51 to -3.54) |
| Syrian Arab Republic | 123.929 (43.282-272.517) | 5.27 (1.797-11.689) | 89.365 (29.97-182.944) | 2.353 (0.786-4.851) | -2.63 (-2.85 to -2.42) |
| Taiwan (Province of China) | 354.996 (111.744-779.057) | 6.427 (2.021-14.091) | 167.345 (56.04-358.722) | 2.235 (0.761-4.777) | -2.46 (-2.85 to -2.07) |
| Togolese Republic | 72.583 (25.785-160.486) | 8.991 (3.187-19.576) | 160.581 (59.899-348.711) | 7.583 (2.83-16.474) | -0.07 (-0.4 to 0.25) |
| Tokelau | 0.026 (0.012-0.05) | 7.742 (3.425-14.672) | 0.019 (0.009-0.034) | 5.797 (2.746-10.666) | -1.49 (-1.71 to -1.28) |
| Turkmenistan | 1269.041 (755.827-1945.598) | 127.973 (75.66-197.776) | 358.241 (190.694-632.976) | 28.159 (14.978-49.753) | -4.64 (-5.03 to -4.25) |
| Tuvalu | 0.198 (0.08-0.392) | 8.202 (3.304-16.238) | 0.13 (0.054-0.249) | 4.741 (1.972-9.104) | -1.85 (-2.04 to -1.66) |
| Ukraine | 1040.809 (696.214-1503.883) | 8.69 (5.828-12.512) | 258.079 (131.135-454.887) | 2.678 (1.341-4.742) | -3.56 (-4.7 to -2.41) |
| Union of the Comoros | 24.971 (6.982-61.344) | 24.353 (7.072-58.883) | 33.361 (11.994-74.686) | 17.12 (6.212-38.095) | -1.72 (-2.24 to -1.2) |
| United Arab Emirates | 20.437 (8.901-39.126) | 7.228 (3.086-13.993) | 57.136 (30.034-95.155) | 2.89 (1.475-4.924) | -2.53 (-2.78 to -2.27) |
| United Kingdom of Great Britain and Northern Ireland | 74.146 (30.736-153.565) | 0.511 (0.213-1.059) | 92.534 (43.379-182.829) | 0.573 (0.271-1.126) | 0.82 (0.26 to 1.37) |
| United Mexican States | 117.111 (67.646-205.554) | 0.588 (0.338-1.036) | 312.93 (228.182-431.565) | 0.876 (0.638-1.21) | 2.09 (1.33 to 2.86) |
| United Republic of Tanzania | 2359.545 (561.18-5733.468) | 40.071 (9.773-96.382) | 1967.783 (660.353-4500.16) | 13.451 (4.594-30.43) | -3.74 (-4.01 to -3.46) |
| United States Virgin Islands | 0.22 (0.112-0.393) | 0.767 (0.393-1.367) | 0.121 (0.057-0.223) | 0.68 (0.314-1.258) | 0.16 (-0.73 to 1.05) |
| United States of America | 492.202 (358.576-704.977) | 0.721 (0.529-1.025) | 280.592 (183.383-445.025) | 0.349 (0.23-0.549) | -2.7 (-3.07 to -2.32) |
| (SDI: Socio-Demographic Index, ASR: Age-standardized rate, EAPC: Estimated Annual Percentage Change, UI: uncertainty intervals, CI: confidence intervals, AHB: Acute hepatitis B, ASDR: Age-standardized rate of disability-adjusted life years, DALYs: Disability-Adjusted Life Years) | | | | | |

| **Table S5. Global, SDI and regional AAPC of AHB burden in WCBA (1990–2021).** | | | | | |
| --- | --- | --- | --- | --- | --- |
| **measure** | **location** | **Joinpoint Number** | **AAPC** | **AAPC 95%CI** | **P-Value** |
| DALYs | Global | 6 | -1.383 | -1.415 to -1.354 | 0 |
| DALYs | High SDI | 4 | -2.762 | -2.812 to -2.708 | 0 |
| DALYs | High-middle SDI | 6 | -3.501 | -3.57 to -3.451 | 0 |
| DALYs | Low SDI | 5 | -2.654 | -2.688 to -2.611 | 0 |
| DALYs | Low-middle SDI | 5 | -1.008 | -1.058 to -0.966 | 0 |
| DALYs | Middle SDI | 6 | -2.487 | -2.542 to -2.447 | 0 |
| DALYs | Andean Latin America | 3 | -0.606 | -0.992 to -0.219 | 0.002 |
| DALYs | Australasia | 6 | -1.163 | -1.724 to -0.598 | 0 |
| DALYs | Caribbean | 5 | 1.046 | 0.6 to 1.494 | 0 |
| DALYs | Central Asia | 6 | -6.063 | -6.345 to -5.782 | 0 |
| DALYs | Central Europe | 3 | -2.051 | -2.501 to -1.599 | 0 |
| DALYs | Central Latin America | 6 | -0.15 | -0.49 to 0.191 | 0.389 |
| DALYs | Central Sub-Saharan Africa | 4 | -3.314 | -3.663 to -2.964 | 0 |
| DALYs | East Asia | 4 | -5.96 | -6.231 to -5.689 | 0 |
| DALYs | Eastern Europe | 3 | -4.124 | -4.773 to -3.471 | 0 |
| DALYs | Eastern Sub-Saharan Africa | 5 | -2.48 | -2.567 to -2.393 | 0 |
| DALYs | High-income Asia Pacific | 6 | -2.584 | -3.029 to -2.137 | 0 |
| DALYs | High-income North America | 6 | -2.005 | -2.537 to -1.47 | 0 |
| DALYs | North Africa and Middle East | 5 | -3.775 | -4.118 to -3.431 | 0 |
| DALYs | Oceania | 2 | -2.499 | -2.605 to -2.393 | 0 |
| DALYs | South Asia | 4 | 0.676 | 0.175 to 1.179 | 0.008 |
| DALYs | Southeast Asia | 6 | -2.67 | -2.849 to -2.491 | 0 |
| DALYs | Southern Latin America | 5 | -1.73 | -2.259 to -1.197 | 0 |
| DALYs | Southern Sub-Saharan Africa | 6 | -2.933 | -3.608 to -2.254 | 0 |
| DALYs | Tropical Latin America | 5 | -1.139 | -1.544 to -0.731 | 0 |
| DALYs | Western Europe | 5 | -1.991 | -2.194 to -1.788 | 0 |
| DALYs | Western Sub-Saharan Africa | 4 | -4.934 | -5.236 to -4.63 | 0 |
| Deaths | Global | 6 | -1.559 | -1.59 to -1.531 | 0 |
| Deaths | High SDI | 6 | -4.177 | -4.328 to -3.995 | 0 |
| Deaths | High-middle SDI | 5 | -4.382 | -4.511 to -4.272 | 0 |
| Deaths | Low SDI | 5 | -2.859 | -2.893 to -2.819 | 0 |
| Deaths | Low-middle SDI | 4 | -1.104 | -1.171 to -1.054 | 0 |
| Deaths | Middle SDI | 6 | -2.805 | -2.873 to -2.746 | 0 |
| Deaths | Andean Latin America | 6 | -1.336 | -2.389 to -0.271 | 0.014 |
| Deaths | Australasia | 6 | -1.285 | -7.177 to 4.98 | 0.68 |
| Deaths | Caribbean | 4 | 1.193 | 0.558 to 1.832 | 0 |
| Deaths | Central Asia | 6 | -6.409 | -6.709 to -6.108 | 0 |
| Deaths | Central Europe | 6 | -2.244 | -3.364 to -1.111 | 0 |
| Deaths | Central Latin America | 6 | 3.693 | 2.942 to 4.45 | 0 |
| Deaths | Central Sub-Saharan Africa | 4 | -3.857 | -4.24 to -3.472 | 0 |
| Deaths | East Asia | 4 | -8.842 | -9.158 to -8.525 | 0 |
| Deaths | Eastern Europe | 3 | -4.957 | -5.905 to -4 | 0 |
| Deaths | Eastern Sub-Saharan Africa | 6 | -2.65 | -2.779 to -2.521 | 0 |
| Deaths | High-income Asia Pacific | 6 | -4.956 | -6.57 to -3.313 | 0 |
| Deaths | High-income North America | 5 | -2.619 | -3.513 to -1.717 | 0 |
| Deaths | North Africa and Middle East | 5 | -4.048 | -4.416 to -3.679 | 0 |
| Deaths | Oceania | 5 | -4.992 | -5.33 to -4.652 | 0 |
| Deaths | South Asia | 3 | 0.719 | 0.388 to 1.051 | 0 |
| Deaths | Southeast Asia | 6 | -3.001 | -3.221 to -2.782 | 0 |
| Deaths | Southern Latin America | 5 | -4.103 | -5.373 to -2.815 | 0 |
| Deaths | Southern Sub-Saharan Africa | 5 | -3.37 | -4.133 to -2.601 | 0 |
| Deaths | Tropical Latin America | 4 | -0.075 | -0.837 to 0.693 | 0.848 |
| Deaths | Western Europe | 3 | -3.604 | -4.095 to -3.11 | 0 |
| Deaths | Western Sub-Saharan Africa | 4 | -5.392 | -5.698 to -5.085 | 0 |
| Incidence | Global | 3 | -1.297 | -1.322 to -1.277 | 0 |
| Incidence | High SDI | 4 | -2.092 | -2.138 to -2.051 | 0 |
| Incidence | High-middle SDI | 4 | -2.349 | -2.452 to -2.281 | 0 |
| Incidence | Low SDI | 2 | -0.524 | -0.538 to -0.51 | 0 |
| Incidence | Low-middle SDI | 4 | -0.831 | -0.845 to -0.817 | 0 |
| Incidence | Middle SDI | 4 | -1.962 | -2.017 to -1.925 | 0 |
| Incidence | Andean Latin America | 5 | -0.641 | -0.731 to -0.55 | 0 |
| Incidence | Australasia | 4 | -1.597 | -1.689 to -1.505 | 0 |
| Incidence | Caribbean | 4 | -0.812 | -0.88 to -0.744 | 0 |
| Incidence | Central Asia | 4 | -0.736 | -0.916 to -0.556 | 0 |
| Incidence | Central Europe | 5 | -2.663 | -2.788 to -2.539 | 0 |
| Incidence | Central Latin America | 4 | -2.088 | -2.318 to -1.858 | 0 |
| Incidence | Central Sub-Saharan Africa | 2 | -0.207 | -0.325 to -0.088 | 0.001 |
| Incidence | East Asia | 3 | -2.414 | -2.575 to -2.253 | 0 |
| Incidence | Eastern Europe | 5 | -1.494 | -1.671 to -1.317 | 0 |
| Incidence | Eastern Sub-Saharan Africa | 2 | -0.879 | -0.955 to -0.803 | 0 |
| Incidence | High-income Asia Pacific | 3 | -1.762 | -1.914 to -1.61 | 0 |
| Incidence | High-income North America | 3 | -1.805 | -1.994 to -1.616 | 0 |
| Incidence | North Africa and Middle East | 3 | -1.658 | -1.732 to -1.585 | 0 |
| Incidence | Oceania | 1 | -1.173 | -1.252 to -1.094 | 0 |
| Incidence | South Asia | 6 | -0.706 | -0.723 to -0.688 | 0 |
| Incidence | Southeast Asia | 6 | -1.215 | -1.298 to -1.132 | 0 |
| Incidence | Southern Latin America | 2 | -0.698 | -0.785 to -0.612 | 0 |
| Incidence | Southern Sub-Saharan Africa | 3 | -1.451 | -1.585 to -1.316 | 0 |
| Incidence | Tropical Latin America | 5 | -2.714 | -2.845 to -2.583 | 0 |
| Incidence | Western Europe | 2 | -1.735 | -1.799 to -1.671 | 0 |
| Incidence | Western Sub-Saharan Africa | 5 | -0.637 | -0.669 to -0.606 | 0 |
| (AHB: Acute hepatitis B, WCBA: Women of childbearing age, DALYs: disability-adjusted life years, SDI: Socio-Demographic Index, AAPC: Average Annual Percent Change, CI: Confidence Interval) | | | | | |

| **Table S6. Global, SDI and regional APC of AHB burden in WCBA (1990–2021).** | | | | | |
| --- | --- | --- | --- | --- | --- |
| **measure** | **location** | **Segment** | **Period** | **APC_95%CI** | **P-Value** |
| DALYs | Global | 0 | 1990-2000 | -0.741(-0.952 to -0.58) | 0.003 |
| DALYs | Global | 1 | 2000-2003 | -2.532(-2.751 to -0.554) | 0 |
| DALYs | Global | 2 | 2003-2009 | -1.849(-2.428 to -1.569) | 0 |
| DALYs | Global | 3 | 2009-2012 | -0.563(-1.803 to -0.303) | 0 |
| DALYs | Global | 4 | 2012-2015 | -2.837(-3.096 to -0.829) | 0 |
| DALYs | Global | 5 | 2015-2019 | -2.069(-2.402 to -1.722) | 0 |
| DALYs | Global | 6 | 2019-2021 | 0.928(0.296 to 1.405) | 0.01 |
| DALYs | High SDI | 0 | 1990-1993 | -3.292(-3.864 to -2.648) | 0 |
| DALYs | High SDI | 1 | 1993-1997 | 2.307(1.964 to 2.819) | 0 |
| DALYs | High SDI | 2 | 1997-2000 | -0.638(-1.049 to -0.1) | 0.023 |
| DALYs | High SDI | 3 | 2000-2008 | -5.759(-5.943 to -5.61) | 0 |
| DALYs | High SDI | 4 | 2008-2021 | -2.77(-2.896 to -2.655) | 0 |
| DALYs | High-middle SDI | 0 | 1990-1994 | -1.155(-1.426 to -0.614) | 0.004 |
| DALYs | High-middle SDI | 1 | 1994-1997 | -2.841(-3.195 to -2.24) | 0 |
| DALYs | High-middle SDI | 2 | 1997-2000 | -1.729(-6.839 to -1.362) | 0 |
| DALYs | High-middle SDI | 3 | 2000-2004 | -7.068(-7.311 to -3.497) | 0 |
| DALYs | High-middle SDI | 4 | 2004-2016 | -3.33(-3.421 to -3.132) | 0 |
| DALYs | High-middle SDI | 5 | 2016-2019 | -5.713(-6.238 to -4.925) | 0 |
| DALYs | High-middle SDI | 6 | 2019-2021 | -2.146(-3.38 to -1.146) | 0 |
| DALYs | Low SDI | 0 | 1990-1996 | 0.812(0.534 to 1.184) | 0.002 |
| DALYs | Low SDI | 1 | 1996-2000 | -0.922(-1.894 to -0.506) | 0.005 |
| DALYs | Low SDI | 2 | 2000-2010 | -4.41(-4.658 to -4.32) | 0 |
| DALYs | Low SDI | 3 | 2010-2013 | -3.396(-3.909 to -3.105) | 0 |
| DALYs | Low SDI | 4 | 2013-2016 | -5.517(-5.838 to -5.012) | 0 |
| DALYs | Low SDI | 5 | 2016-2021 | -2.379(-2.611 to -2.136) | 0 |
| DALYs | Low-middle SDI | 0 | 1990-1997 | -0.575(-1.091 to -0.346) | 0.014 |
| DALYs | Low-middle SDI | 1 | 1997-2005 | 0.107(-0.137 to 0.602) | 0.266 |
| DALYs | Low-middle SDI | 2 | 2005-2012 | -1.193(-1.415 to -0.007) | 0.05 |
| DALYs | Low-middle SDI | 3 | 2012-2015 | -3.665(-4.003 to -1.191) | 0 |
| DALYs | Low-middle SDI | 4 | 2015-2019 | -2.331(-3.083 to -1.864) | 0 |
| DALYs | Low-middle SDI | 5 | 2019-2021 | 0.414(-0.588 to 1.081) | 0.34 |
| DALYs | Middle SDI | 0 | 1990-1999 | -3.458(-3.705 to -3.245) | 0 |
| DALYs | Middle SDI | 1 | 1999-2004 | -4.417(-4.954 to -3.241) | 0 |
| DALYs | Middle SDI | 2 | 2004-2008 | -2.388(-4.545 to -1.748) | 0 |
| DALYs | Middle SDI | 3 | 2008-2013 | 0.124(-2.476 to 0.682) | 0.454 |
| DALYs | Middle SDI | 4 | 2013-2016 | -2.427(-2.759 to 0.395) | 0.148 |
| DALYs | Middle SDI | 5 | 2016-2019 | -3.755(-4.252 to -3.039) | 0 |
| DALYs | Middle SDI | 6 | 2019-2021 | 2.073(1.027 to 2.894) | 0.001 |
| Deaths | Global | 0 | 1990-2000 | -1.049(-1.155 to -0.949) | 0 |
| Deaths | Global | 1 | 2000-2004 | -2.922(-3.268 to -1.073) | 0 |
| Deaths | Global | 2 | 2004-2009 | -1.975(-2.761 to -1.648) | 0 |
| Deaths | Global | 3 | 2009-2013 | -0.974(-1.823 to -0.571) | 0 |
| Deaths | Global | 4 | 2013-2016 | -2.979(-3.273 to -1.13) | 0 |
| Deaths | Global | 5 | 2016-2019 | -1.719(-2.478 to -1.417) | 0 |
| Deaths | Global | 6 | 2019-2021 | 0.932(0.312 to 1.446) | 0.009 |
| Deaths | High SDI | 0 | 1990-1993 | -6.037(-10.041 to -3.97) | 0 |
| Deaths | High SDI | 1 | 1993-1996 | 5.95(4.362 to 7.361) | 0 |
| Deaths | High SDI | 2 | 1996-1999 | 1.806(0.711 to 2.685) | 0.039 |
| Deaths | High SDI | 3 | 1999-2002 | -6.88(-8.259 to -6.261) | 0 |
| Deaths | High SDI | 4 | 2002-2007 | -12.147(-12.536 to -11.72) | 0 |
| Deaths | High SDI | 5 | 2007-2012 | -6.515(-7.339 to -5.257) | 0.001 |
| Deaths | High SDI | 6 | 2012-2021 | -1.813(-2.268 to -1.257) | 0.002 |
| Deaths | High-middle SDI | 0 | 1990-1993 | -1.413(-2.613 to 0.687) | 0.13 |
| Deaths | High-middle SDI | 1 | 1993-2000 | -3.213(-4.446 to -2.976) | 0 |
| Deaths | High-middle SDI | 2 | 2000-2005 | -9.055(-9.36 to -8.657) | 0 |
| Deaths | High-middle SDI | 3 | 2005-2016 | -3.497(-3.674 to -3.273) | 0 |
| Deaths | High-middle SDI | 4 | 2016-2019 | -7.348(-8.289 to -5.933) | 0 |
| Deaths | High-middle SDI | 5 | 2019-2021 | -1.137(-3.454 to 0.732) | 0.204 |
| Deaths | Low SDI | 0 | 1990-1996 | 0.667(0.403 to 1.013) | 0 |
| Deaths | Low SDI | 1 | 1996-2000 | -1.067(-1.903 to -0.688) | 0 |
| Deaths | Low SDI | 2 | 2000-2010 | -4.722(-4.887 to -4.631) | 0 |
| Deaths | Low SDI | 3 | 2010-2013 | -3.412(-3.889 to -3.121) | 0 |
| Deaths | Low SDI | 4 | 2013-2016 | -5.782(-6.099 to -5.357) | 0 |
| Deaths | Low SDI | 5 | 2016-2021 | -2.562(-2.784 to -2.335) | 0 |
| Deaths | Low-middle SDI | 0 | 1990-1997 | -0.73(-1.574 to -0.462) | 0.002 |
| Deaths | Low-middle SDI | 1 | 1997-2005 | -0.146(-0.383 to 0.502) | 0.52 |
| Deaths | Low-middle SDI | 2 | 2005-2012 | -1.369(-1.726 to -1.03) | 0 |
| Deaths | Low-middle SDI | 3 | 2012-2019 | -2.804(-3.254 to -2.585) | 0 |
| Deaths | Low-middle SDI | 4 | 2019-2021 | 0.716(-0.625 to 1.494) | 0.205 |
| Deaths | Middle SDI | 0 | 1990-1993 | -3.306(-3.901 to -2.371) | 0 |
| Deaths | Middle SDI | 1 | 1993-2000 | -4.351(-4.677 to -4.166) | 0 |
| Deaths | Middle SDI | 2 | 2000-2005 | -5.365(-5.869 to -5.093) | 0 |
| Deaths | Middle SDI | 3 | 2005-2009 | -1.866(-2.665 to -0.979) | 0.018 |
| Deaths | Middle SDI | 4 | 2009-2013 | 0.573(-0.064 to 1.335) | 0.061 |
| Deaths | Middle SDI | 5 | 2013-2019 | -2.977(-3.395 to -2.693) | 0 |
| Deaths | Middle SDI | 6 | 2019-2021 | 2.014(0.489 to 2.928) | 0.013 |
| Incidence | Global | 0 | 1990-2001 | -0.637(-0.687 to -0.581) | 0 |
| Incidence | Global | 1 | 2001-2010 | -1.128(-1.211 to -1.058) | 0 |
| Incidence | Global | 2 | 2010-2019 | -1.905(-1.959 to -1.817) | 0 |
| Incidence | Global | 3 | 2019-2021 | -2.916(-3.331 to -2.402) | 0 |
| Incidence | High SDI | 0 | 1990-1996 | -0.185(-0.394 to 0.109) | 0.162 |
| Incidence | High SDI | 1 | 1996-2005 | -1.166(-1.306 to -1.048) | 0 |
| Incidence | High SDI | 2 | 2005-2011 | -2.356(-2.617 to -2.032) | 0 |
| Incidence | High SDI | 3 | 2011-2019 | -3.495(-3.62 to -3.339) | 0 |
| Incidence | High SDI | 4 | 2019-2021 | -5.406(-6.095 to -4.486) | 0 |
| Incidence | High-middle SDI | 0 | 1990-2003 | -0.543(-0.886 to -0.257) | 0.048 |
| Incidence | High-middle SDI | 1 | 2003-2010 | -2.012(-2.472 to -0.247) | 0.024 |
| Incidence | High-middle SDI | 2 | 2010-2014 | -4.608(-5.381 to -1.777) | 0 |
| Incidence | High-middle SDI | 3 | 2014-2019 | -3.432(-3.971 to -2.466) | 0 |
| Incidence | High-middle SDI | 4 | 2019-2021 | -7.718(-9.457 to -6.015) | 0 |
| Incidence | Low SDI | 0 | 1990-2001 | -0.074(-0.12 to -0.019) | 0.012 |
| Incidence | Low SDI | 1 | 2001-2010 | -0.479(-0.565 to -0.407) | 0 |
| Incidence | Low SDI | 2 | 2010-2021 | -1.01(-1.063 to -0.96) | 0 |
| Incidence | Low-middle SDI | 0 | 1990-2006 | -0.431(-0.461 to -0.39) | 0 |
| Incidence | Low-middle SDI | 1 | 2006-2011 | -0.766(-0.869 to -0.4) | 0 |
| Incidence | Low-middle SDI | 2 | 2011-2016 | -1.141(-1.217 to -0.748) | 0 |
| Incidence | Low-middle SDI | 3 | 2016-2019 | -1.57(-1.681 to -1.169) | 0 |
| Incidence | Low-middle SDI | 4 | 2019-2021 | -2.285(-2.553 to -1.939) | 0 |
| Incidence | Middle SDI | 0 | 1990-2001 | -1.364(-1.821 to -1.187) | 0.002 |
| Incidence | Middle SDI | 1 | 2001-2004 | -2.139(-2.44 to -0.936) | 0 |
| Incidence | Middle SDI | 2 | 2004-2011 | -1.498(-1.931 to -0.922) | 0 |
| Incidence | Middle SDI | 3 | 2011-2017 | -2.496(-2.729 to -2.127) | 0 |
| Incidence | Middle SDI | 4 | 2017-2021 | -3.466(-4.285 to -3.126) | 0 |
| DALYs | Andean Latin America | 0 | 1990-1998 | 0.892(0.483 to 1.302) | 0 |
| DALYs | Andean Latin America | 1 | 1998-2004 | -0.085(-0.835 to 0.67) | 0.816 |
| DALYs | Andean Latin America | 2 | 2004-2007 | -4.295(-7.815 to -0.64) | 0.024 |
| DALYs | Andean Latin America | 3 | 2007-2021 | -0.873(-1.054 to -0.691) | 0 |
| DALYs | Australasia | 0 | 1990-1992 | 1.092(-2.066 to 4.352) | 0.47 |
| DALYs | Australasia | 1 | 1992-1995 | -3.596(-6.516 to -0.585) | 0.023 |
| DALYs | Australasia | 2 | 1995-1998 | 2.326(-0.835 to 5.587) | 0.136 |
| DALYs | Australasia | 3 | 1998-2001 | -0.747(-3.918 to 2.528) | 0.624 |
| DALYs | Australasia | 4 | 2001-2005 | -3.674(-5.211 to -2.112) | 0 |
| DALYs | Australasia | 5 | 2005-2018 | -0.817(-1.014 to -0.619) | 0 |
| DALYs | Australasia | 6 | 2018-2021 | -2.127(-3.836 to -0.388) | 0.021 |
| DALYs | Caribbean | 0 | 1990-1997 | 3.381(2.885 to 3.878) | 0 |
| DALYs | Caribbean | 1 | 1997-2000 | -1.098(-4.22 to 2.125) | 0.474 |
| DALYs | Caribbean | 2 | 2000-2004 | 5.584(3.769 to 7.43) | 0 |
| DALYs | Caribbean | 3 | 2004-2010 | -1.971(-2.79 to -1.145) | 0 |
| DALYs | Caribbean | 4 | 2010-2017 | -0.37(-1.009 to 0.272) | 0.238 |
| DALYs | Caribbean | 5 | 2017-2021 | 1.283(0.022 to 2.56) | 0.047 |
| DALYs | Central Asia | 0 | 1990-1996 | -5.297(-5.596 to -4.997) | 0 |
| DALYs | Central Asia | 1 | 1996-1999 | -8.565(-10.21 to -6.89) | 0 |
| DALYs | Central Asia | 2 | 1999-2003 | -4.498(-5.368 to -3.619) | 0 |
| DALYs | Central Asia | 3 | 2003-2007 | -9.965(-10.786 to -9.136) | 0 |
| DALYs | Central Asia | 4 | 2007-2012 | -4.35(-4.94 to -3.755) | 0 |
| DALYs | Central Asia | 5 | 2012-2019 | -6.378(-6.717 to -6.038) | 0 |
| DALYs | Central Asia | 6 | 2019-2021 | -2.773(-5.421 to -0.051) | 0.047 |
| DALYs | Central Europe | 0 | 1990-2001 | 1.422(1.05 to 1.795) | 0 |
| DALYs | Central Europe | 1 | 2001-2013 | -4.705(-5.034 to -4.374) | 0 |
| DALYs | Central Europe | 2 | 2013-2018 | -0.952(-2.812 to 0.945) | 0.306 |
| DALYs | Central Europe | 3 | 2018-2021 | -5.564(-8.746 to -2.271) | 0.002 |
| DALYs | Central Latin America | 0 | 1990-1995 | -0.817(-1.57 to -0.057) | 0.037 |
| DALYs | Central Latin America | 1 | 1995-1999 | 2.007(0.418 to 3.621) | 0.017 |
| DALYs | Central Latin America | 2 | 1999-2005 | 0.829(0.153 to 1.509) | 0.02 |
| DALYs | Central Latin America | 3 | 2005-2009 | 5.455(4.187 to 6.739) | 0 |
| DALYs | Central Latin America | 4 | 2009-2013 | -2.279(-3.323 to -1.224) | 0.001 |
| DALYs | Central Latin America | 5 | 2013-2018 | -4.813(-5.477 to -4.144) | 0 |
| DALYs | Central Latin America | 6 | 2018-2021 | -0.271(-1.501 to 0.974) | 0.642 |
| DALYs | Central Sub-Saharan Africa | 0 | 1990-1997 | -2.677(-3.34 to -2.01) | 0 |
| DALYs | Central Sub-Saharan Africa | 1 | 1997-2005 | -0.787(-1.44 to -0.13) | 0.022 |
| DALYs | Central Sub-Saharan Africa | 2 | 2005-2013 | -4.622(-5.169 to -4.071) | 0 |
| DALYs | Central Sub-Saharan Africa | 3 | 2013-2017 | -7.473(-9.14 to -5.775) | 0 |
| DALYs | Central Sub-Saharan Africa | 4 | 2017-2021 | -2.526(-3.593 to -1.448) | 0 |
| DALYs | East Asia | 0 | 1990-1992 | -3.456(-6.421 to -0.396) | 0.029 |
| DALYs | East Asia | 1 | 1992-2001 | -6.343(-6.607 to -6.079) | 0 |
| DALYs | East Asia | 2 | 2001-2005 | -14.224(-15.276 to -13.158) | 0 |
| DALYs | East Asia | 3 | 2005-2014 | -3.385(-3.698 to -3.072) | 0 |
| DALYs | East Asia | 4 | 2014-2021 | -4.486(-4.983 to -3.988) | 0 |
| DALYs | Eastern Europe | 0 | 1990-2000 | 1.597(1.091 to 2.106) | 0 |
| DALYs | Eastern Europe | 1 | 2000-2008 | -4.528(-5.383 to -3.665) | 0 |
| DALYs | Eastern Europe | 2 | 2008-2017 | -9.518(-10.5 to -8.525) | 0 |
| DALYs | Eastern Europe | 3 | 2017-2021 | -4.717(-8.92 to -0.319) | 0.037 |
| DALYs | Eastern Sub-Saharan Africa | 0 | 1990-1995 | 1.622(1.439 to 1.805) | 0 |
| DALYs | Eastern Sub-Saharan Africa | 1 | 1995-1999 | -1.727(-2.101 to -1.352) | 0 |
| DALYs | Eastern Sub-Saharan Africa | 2 | 1999-2009 | -3.964(-4.026 to -3.903) | 0 |
| DALYs | Eastern Sub-Saharan Africa | 3 | 2009-2014 | -3.413(-3.609 to -3.217) | 0 |
| DALYs | Eastern Sub-Saharan Africa | 4 | 2014-2017 | -4.331(-4.951 to -3.708) | 0 |
| DALYs | Eastern Sub-Saharan Africa | 5 | 2017-2021 | -1.937(-2.143 to -1.73) | 0 |
| DALYs | High-income Asia Pacific | 0 | 1990-1993 | -1.947(-3.97 to 0.12) | 0.062 |
| DALYs | High-income Asia Pacific | 1 | 1993-1997 | 12.073(10.364 to 13.808) | 0 |
| DALYs | High-income Asia Pacific | 2 | 1997-2000 | 1.525(-0.719 to 3.821) | 0.166 |
| DALYs | High-income Asia Pacific | 3 | 2000-2003 | -5.789(-7.925 to -3.604) | 0 |
| DALYs | High-income Asia Pacific | 4 | 2003-2007 | -13.079(-14.392 to -11.745) | 0 |
| DALYs | High-income Asia Pacific | 5 | 2007-2014 | -4.757(-5.383 to -4.127) | 0 |
| DALYs | High-income Asia Pacific | 6 | 2014-2021 | -2.444(-3.078 to -1.805) | 0 |
| DALYs | High-income North America | 0 | 1990-1993 | -1.292(-2.893 to 0.335) | 0.109 |
| DALYs | High-income North America | 1 | 1993-2000 | 1.049(0.513 to 1.587) | 0.001 |
| DALYs | High-income North America | 2 | 2000-2005 | -3.239(-4.221 to -2.246) | 0 |
| DALYs | High-income North America | 3 | 2005-2008 | -7.604(-10.919 to -4.166) | 0.001 |
| DALYs | High-income North America | 4 | 2008-2013 | -3.416(-4.734 to -2.08) | 0 |
| DALYs | High-income North America | 5 | 2013-2019 | 0.022(-0.959 to 1.013) | 0.962 |
| DALYs | High-income North America | 6 | 2019-2021 | -4.277(-8.611 to 0.262) | 0.062 |
| DALYs | North Africa and Middle East | 0 | 1990-1992 | -5.377(-8.183 to -2.485) | 0.001 |
| DALYs | North Africa and Middle East | 1 | 1992-1999 | -2.239(-2.716 to -1.758) | 0 |
| DALYs | North Africa and Middle East | 2 | 1999-2010 | -4.694(-4.9 to -4.488) | 0 |
| DALYs | North Africa and Middle East | 3 | 2010-2014 | -3.53(-4.746 to -2.299) | 0 |
| DALYs | North Africa and Middle East | 4 | 2014-2017 | -5.445(-7.754 to -3.078) | 0 |
| DALYs | North Africa and Middle East | 5 | 2017-2021 | -2.06(-2.836 to -1.277) | 0 |
| DALYs | Oceania | 0 | 1990-2001 | -1.85(-2.005 to -1.694) | 0 |
| DALYs | Oceania | 1 | 2001-2009 | -3.337(-3.63 to -3.043) | 0 |
| DALYs | Oceania | 2 | 2009-2021 | -2.531(-2.687 to -2.376) | 0 |
| DALYs | South Asia | 0 | 1990-2002 | 1.617(1.329 to 1.906) | 0 |
| DALYs | South Asia | 1 | 2002-2005 | 3.285(-1.232 to 8.009) | 0.146 |
| DALYs | South Asia | 2 | 2005-2012 | 0.658(-0.033 to 1.354) | 0.061 |
| DALYs | South Asia | 3 | 2012-2019 | -2.403(-2.972 to -1.831) | 0 |
| DALYs | South Asia | 4 | 2019-2021 | 2.207(-1.072 to 5.594) | 0.177 |
| DALYs | Southeast Asia | 0 | 1990-1995 | -0.602(-0.839 to -0.366) | 0 |
| DALYs | Southeast Asia | 1 | 1995-2003 | -2.129(-2.264 to -1.994) | 0 |
| DALYs | Southeast Asia | 2 | 2003-2007 | -4.282(-4.792 to -3.769) | 0 |
| DALYs | Southeast Asia | 3 | 2007-2011 | -5.804(-6.321 to -5.285) | 0 |
| DALYs | Southeast Asia | 4 | 2011-2014 | 0.339(-0.791 to 1.482) | 0.527 |
| DALYs | Southeast Asia | 5 | 2014-2017 | -4.592(-5.765 to -3.404) | 0 |
| DALYs | Southeast Asia | 6 | 2017-2021 | -2.264(-2.666 to -1.86) | 0 |
| DALYs | Southern Latin America | 0 | 1990-1994 | -0.983(-2.475 to 0.532) | 0.186 |
| DALYs | Southern Latin America | 1 | 1994-1997 | 5.771(1.663 to 10.046) | 0.009 |
| DALYs | Southern Latin America | 2 | 1997-2002 | -1.032(-2.1 to 0.049) | 0.06 |
| DALYs | Southern Latin America | 3 | 2002-2008 | -6.434(-7.146 to -5.716) | 0 |
| DALYs | Southern Latin America | 4 | 2008-2019 | -1.24(-1.549 to -0.93) | 0 |
| DALYs | Southern Latin America | 5 | 2019-2021 | -3.998(-8.476 to 0.7) | 0.089 |
| DALYs | Southern Sub-Saharan Africa | 0 | 1990-1992 | -2.648(-7.445 to 2.398) | 0.27 |
| DALYs | Southern Sub-Saharan Africa | 1 | 1992-1996 | -11.299(-13.376 to -9.173) | 0 |
| DALYs | Southern Sub-Saharan Africa | 2 | 1996-1999 | 0.048(-4.539 to 4.855) | 0.983 |
| DALYs | Southern Sub-Saharan Africa | 3 | 1999-2004 | 9.345(7.722 to 10.993) | 0 |
| DALYs | Southern Sub-Saharan Africa | 4 | 2004-2009 | -4.129(-5.535 to -2.702) | 0 |
| DALYs | Southern Sub-Saharan Africa | 5 | 2009-2017 | -6.614(-7.263 to -5.96) | 0 |
| DALYs | Southern Sub-Saharan Africa | 6 | 2017-2021 | -1.969(-3.605 to -0.304) | 0.024 |
| DALYs | Tropical Latin America | 0 | 1990-1993 | 2.72(0.285 to 5.214) | 0.031 |
| DALYs | Tropical Latin America | 1 | 1993-1998 | 5.889(4.674 to 7.119) | 0 |
| DALYs | Tropical Latin America | 2 | 1998-2003 | -3.282(-4.299 to -2.254) | 0 |
| DALYs | Tropical Latin America | 3 | 2003-2008 | 0.996(-0.052 to 2.055) | 0.061 |
| DALYs | Tropical Latin America | 4 | 2008-2018 | -5.77(-6.072 to -5.468) | 0 |
| DALYs | Tropical Latin America | 5 | 2018-2021 | -0.333(-2.453 to 1.833) | 0.745 |
| DALYs | Western Europe | 0 | 1990-1993 | 1.175(0.366 to 1.99) | 0.007 |
| DALYs | Western Europe | 1 | 1993-1996 | 4.647(3.178 to 6.137) | 0 |
| DALYs | Western Europe | 2 | 1996-1999 | -1.562(-2.73 to -0.381) | 0.013 |
| DALYs | Western Europe | 3 | 1999-2010 | -2.679(-2.789 to -2.569) | 0 |
| DALYs | Western Europe | 4 | 2010-2017 | -4.616(-4.898 to -4.333) | 0 |
| DALYs | Western Europe | 5 | 2017-2021 | -2.908(-3.523 to -2.288) | 0 |
| DALYs | Western Sub-Saharan Africa | 0 | 1990-1998 | -1.249(-1.584 to -0.912) | 0 |
| DALYs | Western Sub-Saharan Africa | 1 | 1998-2001 | -3.1(-6.022 to -0.086) | 0.045 |
| DALYs | Western Sub-Saharan Africa | 2 | 2001-2013 | -7.032(-7.18 to -6.884) | 0 |
| DALYs | Western Sub-Saharan Africa | 3 | 2013-2017 | -7.63(-8.434 to -6.819) | 0 |
| DALYs | Western Sub-Saharan Africa | 4 | 2017-2021 | -4.424(-4.935 to -3.91) | 0 |
| Deaths | Andean Latin America | 0 | 1990-1995 | 0.969(-0.796 to 2.766) | 0.257 |
| Deaths | Andean Latin America | 1 | 1995-1998 | 6.098(-0.874 to 13.56) | 0.082 |
| Deaths | Andean Latin America | 2 | 1998-2001 | -2.848(-8.137 to 2.745) | 0.283 |
| Deaths | Andean Latin America | 3 | 2001-2004 | 1.573(-3.624 to 7.05) | 0.53 |
| Deaths | Andean Latin America | 4 | 2004-2007 | -11.615(-15.929 to -7.08) | 0 |
| Deaths | Andean Latin America | 5 | 2007-2011 | -0.19(-2.662 to 2.345) | 0.872 |
| Deaths | Andean Latin America | 6 | 2011-2021 | -2.222(-2.641 to -1.802) | 0 |
| Deaths | Australasia | 0 | 1990-1992 | 6.6(-20.522 to 42.977) | 0.644 |
| Deaths | Australasia | 1 | 1992-1995 | -22.434(-44.822 to 9.037) | 0.13 |
| Deaths | Australasia | 2 | 1995-1998 | 22.568(-13.388 to 73.45) | 0.226 |
| Deaths | Australasia | 3 | 1998-2001 | 0.263(-28.467 to 40.532) | 0.987 |
| Deaths | Australasia | 4 | 2001-2005 | -26.969(-39.4 to -11.988) | 0.003 |
| Deaths | Australasia | 5 | 2005-2011 | -2.346(-11.421 to 7.659) | 0.606 |
| Deaths | Australasia | 6 | 2011-2021 | 10.677(7.266 to 14.197) | 0 |
| Deaths | Caribbean | 0 | 1990-1997 | 4.223(3.416 to 5.036) | 0 |
| Deaths | Caribbean | 1 | 1997-2000 | -1.477(-6.343 to 3.641) | 0.545 |
| Deaths | Caribbean | 2 | 2000-2004 | 6.211(3.41 to 9.087) | 0 |
| Deaths | Caribbean | 3 | 2004-2012 | -2.126(-2.868 to -1.379) | 0 |
| Deaths | Caribbean | 4 | 2012-2021 | 0.599(0.03 to 1.171) | 0.04 |
| Deaths | Central Asia | 0 | 1990-1996 | -5.443(-5.772 to -5.113) | 0 |
| Deaths | Central Asia | 1 | 1996-1999 | -8.837(-10.644 to -6.993) | 0 |
| Deaths | Central Asia | 2 | 1999-2003 | -4.471(-5.408 to -3.526) | 0 |
| Deaths | Central Asia | 3 | 2003-2007 | -10.342(-11.219 to -9.456) | 0 |
| Deaths | Central Asia | 4 | 2007-2012 | -4.846(-5.474 to -4.214) | 0 |
| Deaths | Central Asia | 5 | 2012-2019 | -7.063(-7.415 to -6.71) | 0 |
| Deaths | Central Asia | 6 | 2019-2021 | -2.914(-5.667 to -0.08) | 0.045 |
| Deaths | Central Europe | 0 | 1990-1994 | 1.036(-2.838 to 5.064) | 0.576 |
| Deaths | Central Europe | 1 | 1994-2000 | 5.932(3.768 to 8.141) | 0 |
| Deaths | Central Europe | 2 | 2000-2006 | -5.585(-7.101 to -4.045) | 0 |
| Deaths | Central Europe | 3 | 2006-2009 | -9.841(-16.348 to -2.828) | 0.011 |
| Deaths | Central Europe | 4 | 2009-2013 | -5.109(-8.664 to -1.416) | 0.011 |
| Deaths | Central Europe | 5 | 2013-2018 | -0.382(-2.832 to 2.13) | 0.744 |
| Deaths | Central Europe | 6 | 2018-2021 | -6.637(-10.856 to -2.219) | 0.007 |
| Deaths | Central Latin America | 0 | 1990-1996 | 3.249(2.062 to 4.449) | 0 |
| Deaths | Central Latin America | 1 | 1996-1999 | 13.757(7.442 to 20.442) | 0 |
| Deaths | Central Latin America | 2 | 1999-2004 | 5.207(3.545 to 6.896) | 0 |
| Deaths | Central Latin America | 3 | 2004-2009 | 16.276(14.679 to 17.895) | 0 |
| Deaths | Central Latin America | 4 | 2009-2013 | -2.937(-4.891 to -0.943) | 0.008 |
| Deaths | Central Latin America | 5 | 2013-2017 | -8.308(-10.128 to -6.451) | 0 |
| Deaths | Central Latin America | 6 | 2017-2021 | 0.103(-1.544 to 1.777) | 0.895 |
| Deaths | Central Sub-Saharan Africa | 0 | 1990-1997 | -2.936(-3.637 to -2.229) | 0 |
| Deaths | Central Sub-Saharan Africa | 1 | 1997-2005 | -1.038(-1.741 to -0.33) | 0.007 |
| Deaths | Central Sub-Saharan Africa | 2 | 2005-2013 | -5.176(-5.771 to -4.576) | 0 |
| Deaths | Central Sub-Saharan Africa | 3 | 2013-2017 | -8.84(-10.687 to -6.954) | 0 |
| Deaths | Central Sub-Saharan Africa | 4 | 2017-2021 | -3.241(-4.456 to -2.01) | 0 |
| Deaths | East Asia | 0 | 1990-2001 | -7.651(-7.988 to -7.313) | 0 |
| Deaths | East Asia | 1 | 2001-2005 | -21.334(-22.565 to -20.083) | 0 |
| Deaths | East Asia | 2 | 2005-2008 | -6.646(-8.909 to -4.327) | 0 |
| Deaths | East Asia | 3 | 2008-2014 | -4.208(-4.736 to -3.677) | 0 |
| Deaths | East Asia | 4 | 2014-2021 | -7.823(-8.283 to -7.361) | 0 |
| Deaths | Eastern Europe | 0 | 1990-2000 | 1.645(0.943 to 2.353) | 0 |
| Deaths | Eastern Europe | 1 | 2000-2008 | -4.626(-5.825 to -3.411) | 0 |
| Deaths | Eastern Europe | 2 | 2008-2018 | -11.757(-12.784 to -10.718) | 0 |
| Deaths | Eastern Europe | 3 | 2018-2021 | -3.589(-12.255 to 5.932) | 0.429 |
| Deaths | Eastern Sub-Saharan Africa | 0 | 1990-1995 | 1.57(1.37 to 1.77) | 0 |
| Deaths | Eastern Sub-Saharan Africa | 1 | 1995-1998 | -1.565(-2.388 to -0.735) | 0.001 |
| Deaths | Eastern Sub-Saharan Africa | 2 | 1998-2001 | -3.546(-4.326 to -2.759) | 0 |
| Deaths | Eastern Sub-Saharan Africa | 3 | 2001-2009 | -4.269(-4.366 to -4.171) | 0 |
| Deaths | Eastern Sub-Saharan Africa | 4 | 2009-2014 | -3.657(-3.876 to -3.438) | 0 |
| Deaths | Eastern Sub-Saharan Africa | 5 | 2014-2017 | -4.638(-5.329 to -3.941) | 0 |
| Deaths | Eastern Sub-Saharan Africa | 6 | 2017-2021 | -1.912(-2.145 to -1.679) | 0 |
| Deaths | High-income Asia Pacific | 0 | 1990-1993 | -1.784(-14.666 to 13.042) | 0.785 |
| Deaths | High-income Asia Pacific | 1 | 1993-1997 | 27.063(19.22 to 35.422) | 0 |
| Deaths | High-income Asia Pacific | 2 | 1997-2001 | 0.671(-2.605 to 4.058) | 0.667 |
| Deaths | High-income Asia Pacific | 3 | 2001-2004 | -13.15(-18.358 to -7.61) | 0 |
| Deaths | High-income Asia Pacific | 4 | 2004-2007 | -26.934(-31.724 to -21.809) | 0 |
| Deaths | High-income Asia Pacific | 5 | 2007-2014 | -11.514(-12.59 to -10.424) | 0 |
| Deaths | High-income Asia Pacific | 6 | 2014-2021 | -4(-4.975 to -3.015) | 0 |
| Deaths | High-income North America | 0 | 1990-1993 | -2.386(-6.4 to 1.801) | 0.239 |
| Deaths | High-income North America | 1 | 1993-2000 | 2.107(0.798 to 3.433) | 0.004 |
| Deaths | High-income North America | 2 | 2000-2005 | -5.344(-7.485 to -3.153) | 0 |
| Deaths | High-income North America | 3 | 2005-2008 | -14.532(-19.846 to -8.865) | 0 |
| Deaths | High-income North America | 4 | 2008-2012 | -9.228(-12.188 to -6.167) | 0 |
| Deaths | High-income North America | 5 | 2012-2021 | 2.663(1.877 to 3.456) | 0 |
| Deaths | North Africa and Middle East | 0 | 1990-1992 | -5.591(-8.648 to -2.433) | 0.002 |
| Deaths | North Africa and Middle East | 1 | 1992-1999 | -2.478(-2.998 to -1.956) | 0 |
| Deaths | North Africa and Middle East | 2 | 1999-2009 | -5.161(-5.427 to -4.894) | 0 |
| Deaths | North Africa and Middle East | 3 | 2009-2014 | -3.783(-4.637 to -2.922) | 0 |
| Deaths | North Africa and Middle East | 4 | 2014-2017 | -5.675(-8.262 to -3.015) | 0 |
| Deaths | North Africa and Middle East | 5 | 2017-2021 | -2.281(-3.157 to -1.397) | 0 |
| Deaths | Oceania | 0 | 1990-1996 | -3.169(-3.593 to -2.743) | 0 |
| Deaths | Oceania | 1 | 1996-2002 | -4.386(-4.928 to -3.841) | 0 |
| Deaths | Oceania | 2 | 2002-2009 | -6.863(-7.279 to -6.445) | 0 |
| Deaths | Oceania | 3 | 2009-2015 | -4.182(-4.761 to -3.599) | 0 |
| Deaths | Oceania | 4 | 2015-2018 | -9.106(-11.775 to -6.357) | 0 |
| Deaths | Oceania | 5 | 2018-2021 | -2.779(-4.326 to -1.208) | 0.002 |
| Deaths | South Asia | 0 | 1990-2007 | 1.733(1.554 to 1.913) | 0 |
| Deaths | South Asia | 1 | 2007-2012 | 0.792(-0.524 to 2.126) | 0.225 |
| Deaths | South Asia | 2 | 2012-2019 | -2.07(-2.666 to -1.471) | 0 |
| Deaths | South Asia | 3 | 2019-2021 | 1.863(-1.567 to 5.412) | 0.275 |
| Deaths | Southeast Asia | 0 | 1990-1995 | -0.72(-0.986 to -0.454) | 0 |
| Deaths | Southeast Asia | 1 | 1995-2003 | -2.53(-2.685 to -2.375) | 0 |
| Deaths | Southeast Asia | 2 | 2003-2007 | -4.781(-5.365 to -4.193) | 0 |
| Deaths | Southeast Asia | 3 | 2007-2011 | -6.878(-7.49 to -6.261) | 0 |
| Deaths | Southeast Asia | 4 | 2011-2014 | 1.05(-0.384 to 2.505) | 0.137 |
| Deaths | Southeast Asia | 5 | 2014-2017 | -5.254(-6.712 to -3.773) | 0 |
| Deaths | Southeast Asia | 6 | 2017-2021 | -2.274(-2.783 to -1.761) | 0 |
| Deaths | Southern Latin America | 0 | 1990-1994 | -2.15(-6.527 to 2.433) | 0.328 |
| Deaths | Southern Latin America | 1 | 1994-1997 | 10.588(-0.48 to 22.887) | 0.06 |
| Deaths | Southern Latin America | 2 | 1997-2003 | -3.101(-4.771 to -1.402) | 0.002 |
| Deaths | Southern Latin America | 3 | 2003-2009 | -14.334(-15.693 to -12.952) | 0 |
| Deaths | Southern Latin America | 4 | 2009-2012 | 1.379(-5.483 to 8.739) | 0.683 |
| Deaths | Southern Latin America | 5 | 2012-2021 | -4.745(-5.435 to -4.05) | 0 |
| Deaths | Southern Sub-Saharan Africa | 0 | 1990-1992 | -3.789(-10.908 to 3.898) | 0.301 |
| Deaths | Southern Sub-Saharan Africa | 1 | 1992-1997 | -12.753(-14.69 to -10.771) | 0 |
| Deaths | Southern Sub-Saharan Africa | 2 | 1997-2004 | 9.735(8.452 to 11.034) | 0 |
| Deaths | Southern Sub-Saharan Africa | 3 | 2004-2009 | -3.779(-5.879 to -1.633) | 0.002 |
| Deaths | Southern Sub-Saharan Africa | 4 | 2009-2017 | -7.965(-8.893 to -7.027) | 0 |
| Deaths | Southern Sub-Saharan Africa | 5 | 2017-2021 | -2.396(-4.765 to 0.032) | 0.053 |
| Deaths | Tropical Latin America | 0 | 1990-1998 | 10.385(9.035 to 11.752) | 0 |
| Deaths | Tropical Latin America | 1 | 1998-2003 | -4.166(-6.914 to -1.337) | 0.007 |
| Deaths | Tropical Latin America | 2 | 2003-2008 | 2.506(0.014 to 5.06) | 0.049 |
| Deaths | Tropical Latin America | 3 | 2008-2018 | -7.038(-7.616 to -6.457) | 0 |
| Deaths | Tropical Latin America | 4 | 2018-2021 | 0.172(-3.813 to 4.323) | 0.93 |
| Deaths | Western Europe | 0 | 1990-1997 | 7.903(6.471 to 9.354) | 0 |
| Deaths | Western Europe | 1 | 1997-2011 | -5.273(-5.6 to -4.945) | 0 |
| Deaths | Western Europe | 2 | 2011-2016 | -11.757(-13.545 to -9.931) | 0 |
| Deaths | Western Europe | 3 | 2016-2021 | -5.568(-7.101 to -4.009) | 0 |
| Deaths | Western Sub-Saharan Africa | 0 | 1990-1998 | -1.387(-1.727 to -1.045) | 0 |
| Deaths | Western Sub-Saharan Africa | 1 | 1998-2001 | -3.1(-6.087 to -0.017) | 0.049 |
| Deaths | Western Sub-Saharan Africa | 2 | 2001-2012 | -7.473(-7.657 to -7.288) | 0 |
| Deaths | Western Sub-Saharan Africa | 3 | 2012-2017 | -8.45(-9.013 to -7.884) | 0 |
| Deaths | Western Sub-Saharan Africa | 4 | 2017-2021 | -5.262(-5.82 to -4.701) | 0 |
| Incidence | Andean Latin America | 0 | 1990-1995 | 0.195(-0.006 to 0.397) | 0.056 |
| Incidence | Andean Latin America | 1 | 1995-2000 | -0.574(-0.835 to -0.313) | 0 |
| Incidence | Andean Latin America | 2 | 2000-2005 | 0.178(-0.089 to 0.445) | 0.176 |
| Incidence | Andean Latin America | 3 | 2005-2009 | -1.414(-1.819 to -1.008) | 0 |
| Incidence | Andean Latin America | 4 | 2009-2016 | -0.423(-0.581 to -0.263) | 0 |
| Incidence | Andean Latin America | 5 | 2016-2021 | -2.029(-2.224 to -1.834) | 0 |
| Incidence | Australasia | 0 | 1990-1995 | -0.061(-0.233 to 0.11) | 0.463 |
| Incidence | Australasia | 1 | 1995-2010 | -0.831(-0.865 to -0.797) | 0 |
| Incidence | Australasia | 2 | 2010-2016 | -2.926(-3.095 to -2.758) | 0 |
| Incidence | Australasia | 3 | 2016-2019 | -5.345(-6 to -4.685) | 0 |
| Incidence | Australasia | 4 | 2019-2021 | -1.382(-2.252 to -0.504) | 0.004 |
| Incidence | Caribbean | 0 | 1990-1994 | -1.313(-1.522 to -1.104) | 0 |
| Incidence | Caribbean | 1 | 1994-2005 | -0.124(-0.175 to -0.073) | 0 |
| Incidence | Caribbean | 2 | 2005-2015 | -0.826(-0.883 to -0.77) | 0 |
| Incidence | Caribbean | 3 | 2015-2019 | -2.239(-2.545 to -1.933) | 0 |
| Incidence | Caribbean | 4 | 2019-2021 | -0.628(-1.374 to 0.123) | 0.096 |
| Incidence | Central Asia | 0 | 1990-1996 | 0.841(0.454 to 1.229) | 0 |
| Incidence | Central Asia | 1 | 1996-2005 | -0.781(-0.995 to -0.566) | 0 |
| Incidence | Central Asia | 2 | 2005-2010 | 0.551(-0.125 to 1.232) | 0.104 |
| Incidence | Central Asia | 3 | 2010-2015 | -0.79(-1.44 to -0.135) | 0.021 |
| Incidence | Central Asia | 4 | 2015-2021 | -3.224(-3.589 to -2.857) | 0 |
| Incidence | Central Europe | 0 | 1990-1996 | -0.926(-1.094 to -0.757) | 0 |
| Incidence | Central Europe | 1 | 1996-2006 | -1.805(-1.886 to -1.724) | 0 |
| Incidence | Central Europe | 2 | 2006-2011 | -2.565(-2.867 to -2.261) | 0 |
| Incidence | Central Europe | 3 | 2011-2014 | -4.416(-5.246 to -3.579) | 0 |
| Incidence | Central Europe | 4 | 2014-2019 | -3.164(-3.473 to -2.855) | 0 |
| Incidence | Central Europe | 5 | 2019-2021 | -8.274(-9.292 to -7.244) | 0 |
| Incidence | Central Latin America | 0 | 1990-1993 | -1.753(-2.702 to -0.795) | 0.001 |
| Incidence | Central Latin America | 1 | 1993-2005 | -0.585(-0.71 to -0.461) | 0 |
| Incidence | Central Latin America | 2 | 2005-2016 | -2.816(-2.96 to -2.671) | 0 |
| Incidence | Central Latin America | 3 | 2016-2019 | -5.613(-7.286 to -3.91) | 0 |
| Incidence | Central Latin America | 4 | 2019-2021 | -2.134(-4.226 to 0.005) | 0.05 |
| Incidence | Central Sub-Saharan Africa | 0 | 1990-2004 | 0.122(0.027 to 0.218) | 0.014 |
| Incidence | Central Sub-Saharan Africa | 1 | 2004-2018 | -0.664(-0.77 to -0.558) | 0 |
| Incidence | Central Sub-Saharan Africa | 2 | 2018-2021 | 0.403(-0.707 to 1.526) | 0.462 |
| Incidence | East Asia | 0 | 1990-2001 | -1.173(-1.302 to -1.044) | 0 |
| Incidence | East Asia | 1 | 2001-2010 | -2.068(-2.257 to -1.878) | 0 |
| Incidence | East Asia | 2 | 2010-2019 | -3.18(-3.375 to -2.985) | 0 |
| Incidence | East Asia | 3 | 2019-2021 | -7.177(-9.282 to -5.024) | 0 |
| Incidence | Eastern Europe | 0 | 1990-1995 | -0.395(-0.698 to -0.092) | 0.014 |
| Incidence | Eastern Europe | 1 | 1995-2000 | 2.453(2.042 to 2.865) | 0 |
| Incidence | Eastern Europe | 2 | 2000-2008 | -0.602(-0.767 to -0.438) | 0 |
| Incidence | Eastern Europe | 3 | 2008-2011 | -1.276(-2.637 to 0.103) | 0.067 |
| Incidence | Eastern Europe | 4 | 2011-2019 | -4.013(-4.173 to -3.853) | 0 |
| Incidence | Eastern Europe | 5 | 2019-2021 | -7.383(-8.797 to -5.947) | 0 |
| Incidence | Eastern Sub-Saharan Africa | 0 | 1990-2000 | -0.148(-0.28 to -0.016) | 0.029 |
| Incidence | Eastern Sub-Saharan Africa | 1 | 2000-2015 | -0.8(-0.875 to -0.725) | 0 |
| Incidence | Eastern Sub-Saharan Africa | 2 | 2015-2021 | -2.278(-2.571 to -1.984) | 0 |
| Incidence | High-income Asia Pacific | 0 | 1990-1995 | -0.372(-0.783 to 0.04) | 0.075 |
| Incidence | High-income Asia Pacific | 1 | 1995-2002 | -1.707(-1.993 to -1.421) | 0 |
| Incidence | High-income Asia Pacific | 2 | 2002-2019 | -2.338(-2.401 to -2.275) | 0 |
| Incidence | High-income Asia Pacific | 3 | 2019-2021 | -0.487(-2.472 to 1.539) | 0.62 |
| Incidence | High-income North America | 0 | 1990-2005 | -0.174(-0.262 to -0.086) | 0 |
| Incidence | High-income North America | 1 | 2005-2011 | -1.901(-2.393 to -1.408) | 0 |
| Incidence | High-income North America | 2 | 2011-2019 | -3.431(-3.686 to -3.177) | 0 |
| Incidence | High-income North America | 3 | 2019-2021 | -6.945(-9.231 to -4.602) | 0 |
| Incidence | North Africa and Middle East | 0 | 1990-1995 | -0.587(-0.833 to -0.34) | 0 |
| Incidence | North Africa and Middle East | 1 | 1995-2006 | -1.091(-1.171 to -1.011) | 0 |
| Incidence | North Africa and Middle East | 2 | 2006-2011 | -1.406(-1.747 to -1.064) | 0 |
| Incidence | North Africa and Middle East | 3 | 2011-2021 | -2.931(-3.013 to -2.849) | 0 |
| Incidence | Oceania | 0 | 1990-2004 | -0.157(-0.289 to -0.025) | 0.021 |
| Incidence | Oceania | 1 | 2004-2021 | -2.002(-2.107 to -1.897) | 0 |
| Incidence | South Asia | 0 | 1990-1996 | -0.4(-0.42 to -0.38) | 0 |
| Incidence | South Asia | 1 | 1996-1999 | -1.12(-1.214 to -1.026) | 0 |
| Incidence | South Asia | 2 | 1999-2006 | -0.504(-0.524 to -0.484) | 0 |
| Incidence | South Asia | 3 | 2006-2009 | -1.172(-1.27 to -1.074) | 0 |
| Incidence | South Asia | 4 | 2009-2016 | -0.452(-0.471 to -0.433) | 0 |
| Incidence | South Asia | 5 | 2016-2019 | -0.968(-1.066 to -0.87) | 0 |
| Incidence | South Asia | 6 | 2019-2021 | -1.495(-1.614 to -1.375) | 0 |
| Incidence | Southeast Asia | 0 | 1990-1994 | -0.521(-0.713 to -0.329) | 0 |
| Incidence | Southeast Asia | 1 | 1994-2000 | 0.214(0.078 to 0.35) | 0.005 |
| Incidence | Southeast Asia | 2 | 2000-2006 | -0.847(-0.981 to -0.713) | 0 |
| Incidence | Southeast Asia | 3 | 2006-2011 | -1.091(-1.282 to -0.9) | 0 |
| Incidence | Southeast Asia | 4 | 2011-2016 | -2.263(-2.447 to -2.079) | 0 |
| Incidence | Southeast Asia | 5 | 2016-2019 | -3.492(-4.022 to -2.959) | 0 |
| Incidence | Southeast Asia | 6 | 2019-2021 | -2.167(-2.841 to -1.488) | 0 |
| Incidence | Southern Latin America | 0 | 1990-2000 | 0.464(0.378 to 0.55) | 0 |
| Incidence | Southern Latin America | 1 | 2000-2019 | -0.587(-0.621 to -0.553) | 0 |
| Incidence | Southern Latin America | 2 | 2019-2021 | -7.305(-8.515 to -6.079) | 0 |
| Incidence | Southern Sub-Saharan Africa | 0 | 1990-1995 | 0.163(-0.302 to 0.631) | 0.474 |
| Incidence | Southern Sub-Saharan Africa | 1 | 1995-2000 | -2.199(-2.792 to -1.603) | 0 |
| Incidence | Southern Sub-Saharan Africa | 2 | 2000-2010 | -0.702(-0.874 to -0.529) | 0 |
| Incidence | Southern Sub-Saharan Africa | 3 | 2010-2021 | -2.51(-2.65 to -2.37) | 0 |
| Incidence | Tropical Latin America | 0 | 1990-2005 | -1.255(-1.285 to -1.225) | 0 |
| Incidence | Tropical Latin America | 1 | 2005-2008 | -2.349(-3.065 to -1.628) | 0 |
| Incidence | Tropical Latin America | 2 | 2008-2011 | -3.257(-4.042 to -2.466) | 0 |
| Incidence | Tropical Latin America | 3 | 2011-2014 | -6.125(-6.789 to -5.456) | 0 |
| Incidence | Tropical Latin America | 4 | 2014-2019 | -4.619(-4.866 to -4.372) | 0 |
| Incidence | Tropical Latin America | 5 | 2019-2021 | -3.286(-4.167 to -2.398) | 0 |
| Incidence | Western Europe | 0 | 1990-2005 | -0.832(-0.887 to -0.778) | 0 |
| Incidence | Western Europe | 1 | 2005-2012 | -1.978(-2.202 to -1.754) | 0 |
| Incidence | Western Europe | 2 | 2012-2021 | -3.035(-3.157 to -2.914) | 0 |
| Incidence | Western Sub-Saharan Africa | 0 | 1990-1994 | -0.516(-0.61 to -0.422) | 0 |
| Incidence | Western Sub-Saharan Africa | 1 | 1994-2000 | -0.106(-0.174 to -0.038) | 0.005 |
| Incidence | Western Sub-Saharan Africa | 2 | 2000-2005 | -0.651(-0.741 to -0.56) | 0 |
| Incidence | Western Sub-Saharan Africa | 3 | 2005-2010 | -0.118(-0.209 to -0.027) | 0.014 |
| Incidence | Western Sub-Saharan Africa | 4 | 2010-2014 | -1.374(-1.51 to -1.238) | 0 |
| Incidence | Western Sub-Saharan Africa | 5 | 2014-2021 | -1.098(-1.141 to -1.055) | 0 |
| (AHB: Acute hepatitis B, WCBA: Women of childbearing age, DALYs: disability-adjusted life years, SDI: Socio-Demographic Index, APC: Annual Percent Change, CI: Confidence Interval) | | | | | |
